# In Silico, In Vitro, and Ex Vivo Biological Activity of Some Novel Mebeverine Precursors

**DOI:** 10.3390/biomedicines11020605

**Published:** 2023-02-17

**Authors:** Miglena Milusheva, Vera Gledacheva, Iliyana Stefanova, Mina Pencheva, Rositsa Mihaylova, Yulian Tumbarski, Paraskev Nedialkov, Emiliya Cherneva, Mina Todorova, Stoyanka Nikolova

**Affiliations:** 1 Department of Bioorganic Chemistry, Faculty of Pharmacy, Medical University of Plovdiv, 4002 Plovdiv, Bulgaria; 2 Department of Medical Physics and Biophysics, Faculty of Pharmacy, Medical University of Plovdiv, 4002 Plovdiv, Bulgaria; 3 Laboratory of Experimental Chemotherapy, Department “Pharmacology, Pharmacotherapy and Toxicology”, Faculty of Pharmacy, Medical University, 1431 Sofia, Bulgaria; 4 Department of Microbiology, Technological Faculty, University of Food Technologies, 4002 Plovdiv, Bulgaria; 5 Department of Pharmacognosy, Faculty of Pharmacy, Medical University of Sofia, 1000 Sofia, Bulgaria; 6 Department of Chemistry, Faculty of Pharmacy, Medical University of Sofia, 2 Dunav Str., 1000 Sofia, Bulgaria; 7 Department of Organic Chemistry, Faculty of Chemistry, University of Plovdiv, 4000 Plovdiv, Bulgaria

**Keywords:** synthesis, mebeverine precursor, IBS, in silico, bioelectrical activity, antispasmodics, 5-HT-3 receptor antagonist, antiproliferative, antimicrobial

## Abstract

Irritable bowel syndrome (IBS) is a functional gastroenterological disorder with complex pathogenesis and multifaceted therapy approaches, aimed at alleviating clinical symptoms and improving the life quality of patients. Its treatment includes dietary changes and drugs from various pharmacological groups such as antidiarrheals, anticholinergics, serotonin receptor antagonists, targeting chloride ion channels, etc. The present article is focused on the synthesis and biological evaluation of some mebeverine precursors as potential antispasmodics. Methods: In silico analysis aimed at predicting the pharmacodynamic profile of the compounds was performed. Based on these predictions, ex vivo bioelectrical activity (BEA) and immunohistochemical effects of the compounds were established. A thorough biological evaluation of the compounds was conducted assessing their in vitro antimicrobial and cytotoxic activity. Results: All the newly synthesized compounds exerted drug-like properties, whereby 3-methyl-1-phenylbutan-2-amine **3** showed a significant change in BEA due to Ca^2+^ channel regulation, Ca^2+^ influx modulation, and a subsequent change in smooth muscle cell response. The immunohistochemical studies showed a good correlation with the obtained data on the BEA, defining amine **3** as a leader structure. No cytotoxicity to human malignant leukemic cell lines (LAMA-84, K-562) was observed for all tested compounds. Conclusion: Based on the experimental results, we outlined 3-methyl-1-phenylbutan-2-amine **3** as a potential effective choice for orally active long-term therapy of IBS.

## 1. Introduction

**Figure 1:**
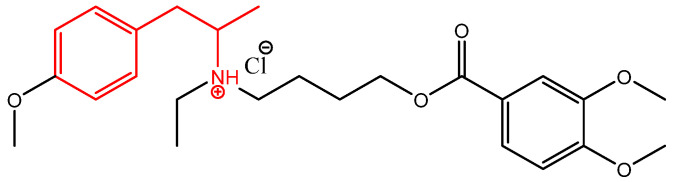
Structure of mebeverine hydrochloride; target compounds common structure in red.

Irritable bowel syndrome (IBS) is a complex syndrome, characterized by abdominal pain and impaired defecation. The exact pathophysiology of IBS is not completely understood [1]. The symptomatic treatment is patient specific; however, antispasmodic drugs are widely used to treat abdominal cramping [2]. Mebeverine is an anticholinergic spasmolytic drug commonly used in the management of IBS for many years (Figure 1). It also acts as a calcium channel blocker, and musculotropic agent and exerts antispasmodic activity and regulatory effects on bowel function [3]. The compound is a second-generation papaverine analog endowed with direct myolytic activity by reducing the excessive contractility of smooth muscle (SM) cells [4].

Despite mebeverine being used to treat IBS, it does not have a statistically significant effect on IBS symptoms [5].

**Figure 2:**
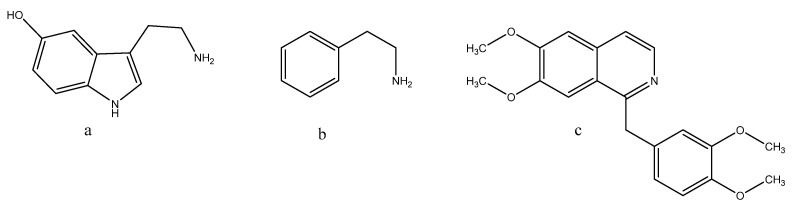
Structures of serotonin (5-HT) (**a**), 2-phenylethylamine (**b**), papaverine (**c**).

Recently, we described the synthesis of 3-methyl-1-phenylbutan-2-amides, their SM relaxant activity, and influence on cognitive functions [6]. The compounds were purposely synthesized as precursors of papaverine, which belongs to the isoquinoline class and isoquinolines can be obtained from phenylethylamines [7] and are known as serotonin (5-HT) agonists (Figure 2).

**Figure 3:**
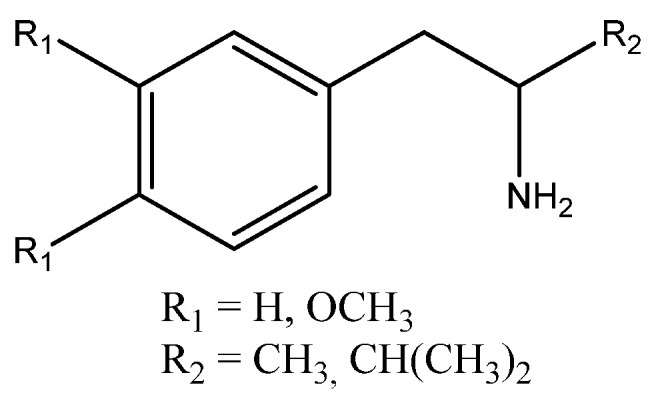
General structure of previously described (R_1_ = OCH_3_, R_2_ = CH_3_) and newly synthesized (R_1_ = H, R_2_ = CH(CH_3_)_2_) 3-substituted 2-phenylethylamines.

Based on their pharmacological activities, we considered the synthesis of 3-methyl-1-phenylbutan-2-amine **3** and its amides. Our choice was also driven by the structural resemblance to mebeverine, which is considered a second-generation papaverine analogue, and its known pharmacological application as an IBS medication. This prompted us to synthesize a number of mebeverine precursors with a C-3 substituent, as well as to investigate their biological activity (Figure 3).

The intrinsic biological activity of compounds can be studied both in vivo and ex vivo using functionally active isolated tissues. The ex vivo method is performed on isolated tissues capable of responding to physiological stimuli and is routinely applied to verify a potential biological action of newly synthesized experimental molecules and validated medicinal agents [8]. In our particular study design, we were inclined to use SM cells as a platform for ex vivo contractility evaluation because they retain the ability to develop active tension even when isolated from the body [9,10]. SM tissue is a main structural element of a number of internal organs. It is related to the motor activity of the stomach and is a complex superposition of bioelectrical (slow wave with its characteristic frequency and amplitude) and contractile activity (CA) (tone, frequency, and amplitude of spontaneous or induced muscle contractions) which can be registered isometrically in isolated tissues [11,12,13]. The bioelectrical activity (BEA) of SM isolated from different segments of the gastrointestinal tract (GIT) varies. In the absence of stimuli of a neuronal nature or endogenous substances, the value of their membrane potential constantly fluctuates. These cyclic changes of depolarization and repolarization are known as slow waves [14]. Slow waves consist of a depolarization, a plateau phase, and a repolarization phase typical for the respective area. It is considered that their frequency is consistent with the maximum possibility of SM shortening, as well as with the physiological purpose of the region. The frequency of the slow wave is relatively constant and typical for each segment of the GIT having 3–10 waves/min [15]. Unlike frequency, the amplitude and duration of slow waves can change under the influence of various humoral factors, as well as in response to drugs and other biologically active substances. Local SM reactivity can also be modulated by neuronal signals. Neurotransmitters change input impedance and activate conductance that generates rhythmically alternating currents in and out of the Cajal cells [16]. Thus, the electrical and mechanical behavior of SM from the GIT, including the stomach, are a composite of the individual electrical activities of pacemaker cells, GM cells, and enteric motor neurons, as well as the quality of coupling between them [17].

The 5-HT_3_ receptor, on the other hand, belongs to the Cys-loop superfamily of ligand-gated ion channels. This ion channel is cation selective for sodium (Na^+^), potassium (K^+^), and calcium (Ca^2+^) ions, and mediates neuronal depolarization and excitation in the central and peripheral nervous systems [18]. Binding of the neurotransmitter 5-hydroxytryptamine (serotonin) to 5-HT_3_ receptor opens the ion channel, which in turn leads to an excitatory response in neurons. The rapidly activating desensitizing inward current is carried primarily by sodium and potassium ions [19]. The 5-HT_3_ receptors are similar in structure to the nicotinic acetylcholine receptor and are also engaged in the regulation of intestinal motility, secretion, and peristalsis in the enteric nervous system and the transmission of information in the GIT [20].

The quest for new therapeutic approaches for IBS to modulate known pharmacological targets or validate new molecules with an alternative mechanism of action is still a priority. Biofilm-related infections, for example, are extremely challenging to treat and pose major concerns for public health. Biofilm formation is one of the major causes of therapeutic failure [21]. Therefore, in addition to the ex vivo spasmolytic activity of our experimental compounds, we sought to determine their in vitro antimicrobial and cytotoxic effects, using mebeverine as a reference drug.

## 2. Materials and Methods

### 2.1. Chemicals

All solvents and reagents were purchased from Merck (Merck Bulgaria EAD). Melting points were determined on a Boetius hot stage apparatus and are uncorrected. All the compounds were characterized by ^1^H NMR, ^13^CNMR, IR, and HRESIMS. The purity of these compounds was determined by TLC using several solvent systems of different polarity. TLC was carried out on precoated 0.2 mm Fluka silica gel 60 plates (Merck KGaA, Darmstadt, Germany), using chloroform: diethyl ether: n-hexane = 6:3:1 as a chromatographic system. Neutral Al_2_O_3_ was used for column chromatographic separation. The products, after evaporation of the solvent, were purified by recrystallization from diethyl ether.

IR spectra were determined on a VERTEX 70 FT-IR spectrometer (Bruker Optics, Ettlingen, Germany). ^1^H NMR and ^13^C NMR spectra were recorded on a Bruker Avance III HD 500 spectrometer (Bruker, Billerica, MA, USA) at 500 MHz (^1^H-NMR) and 125 MHz (^13^C-NMR), respectively. Chemical shifts are given in relative ppm and were referenced to tetramethylsilane (TMS) (δ = 0.00 ppm) as an internal standard; the coupling constants are indicated in Hz. The NMR spectra were recorded at room temperature (ac. 295 K). HRESIMS spectra were acquired in positive mode on Q Exactive Plus (ThermoFisher Scientific, Inc., Bremen, Germany) mass spectrometer, equipped with a heated HESI-II source. Operating conditions for the HESI source used in a positive ionization mode were: +3.5 kV spray voltage, 320 °C capillary and probe heater temperature, sheath gas flow rate 36 a.u., auxiliary gas flow rate 11 a.u., spare gas flow rate 1 a.u. (a.u. refer to arbitrary values set by the Exactive Tune software) and S-Lens RF level 50.00. Nitrogen was used for sample nebulization and collision gas in the HCD cell. The aliquots of 1 µL of the solutions of the samples (ca. 20 µg mL^−1^) were introduced into the mass spectrometer through LC system Thermo Scientific Dionex Ultimate 3000 RSLC (Germering, Germany) consisting of 6-channel degasser SRD-3600, high-pressure gradient pump HPG-3400RS, autosampler WPS-3000TRS, and column compartment TCC-3000RS equipped with narrow bore Hypersil GOLD™ C18 (2.1 × 50 mm, 1.9 μm) column. Each chromatographic run was carried out isocratically with a mobile phase consisting of water-acetonitrile-methanol-acetic acid (25:50:25:0.2). The solvent flow rate was 300 μL min^−1^. Full MS—SIM was used as MS experiment in negative and positive mode, where resolution, automatic gain control (AGC) target, maximum injection time (IT), and mass range were 70,000 (at *m/z* 200), 3e6, 100 ms, and *m/z* 100–500, respectively. Xcalibur (Thermo Fisher Scientific, Waltham, MA, USA) ver. 4.0 was used for data acquisition and processing.

### 2.2. Synthetic Methods Experimental Protocols and Spectral Data

#### 2.2.1. Synthesis of 3-Methyl-1-phenylbutan-2-amine **3**

To a solution of 5 mmol of the starting ketone 3-methyl-1-phenylbutan-2-one **1** in 25 mL formamide, a catalytic amount of methanoic acid was added. The mixture was refluxed for 2 h at 180 °C, then poured in water and extracted with CH_2_Cl_2_ (3 × 20 mL). The combined extracts were washed with Na_2_CO_3_ solution, water, and dried using anhydrous Na_2_SO_4_, filtered on the short column filled with neutral Al_2_O_3,_ and then concentrated. The obtained formamide was directly hydrolyzed with 50 mL 5N H_2_SO_4_ and 1 h reflux at 100 °C to 3-methyl-1-phenylbutan-2-amine **3**. The mixture then was poured in water and extracted with CH_2_Cl_2_ (2 × 20 mL). The water layer was alkalized with NH_4_OH and extracted with CH_2_Cl_2_ (3 × 20 mL). The combined extracts were dried using anhydrous Na_2_SO_4_, filtered on the short column filled with basic Al_2_O_3,_ and then concentrated. Spectral data confirmed the structure of the amine **3** (Supplementary Materials Figures S1–S4).

3-Methyl-1-phenylbutan-2-amine (**3**) ^1^H-NMR: 0.98 (dd, *J* = 9.8, 6.8, 6H, 2xCH_3_), 1.35 (broad s, 2H, NH_2_), 1.67 (dq, *J* = 10.8, 6.8, 1H, CH(CH_3_)_2_), 2.40–2.43 (m, 1H, CHNH_2_), 2.81–2.86 (m,2H, CH_2_), 7.19–7.22 (m, 3H, Ar), 7.28–7.31 (m, 2H, Ar); ^13^C-NMR: 140.3 (Ar), 129.2 (Ar), 128.5 (Ar), 126.1 (Ar), 58.2 (CHNH_2_), 41.3 (CH_2_), 33.1 (CH(CH_3_)_2_), 19.4 (CH(CH_3_)_2_), 17.5 (CH(CH_3_)_2_). IR(KBr) ν_max_, cm^−1^: 3419 ν(N–H), 3071, 3061, 3030 ν(C-H, Ph), 2960 ν^as^(C–H, CH_3_), 2938 ν^as^(C–H, CH_2_), 2898 ν^s^(C–H, CH_3_), 1627 δ(NH_2_), 1590, 1571 ν(C=C, Ph), 1494 δ(CH_2_), 1464 δ^as^(CH_3_), 1375 δ(CH_3_, i-Pr); HRMS (ESI) *m/z* 164.14323.

#### 2.2.2. Preparation of 3-Methyl-1-phenylbutan-2-amides **4**; Typical Procedure

To a solution of 3 mmol 3-methyl-1-phenylbutan-2-amine **3**, 3.5 mmol of the corresponding acyl chloride in dichloromethane (10 mL) was added. Then, 3.4 mmol N(C_2_H_5_)_3_ was added in 10 min. In about 30 min the reaction mixture was washed consequently with diluted HCl (1:4), Na_2_CO_3_, and H_2_O, then dried with anhydrous Na_2_SO_4_, filtered on the short column filled with neutral Al_2_O_3,_ and concentrated. Spectral data confirmed the structure of the amides **4** (Supplementary Materials Figures S5–S20).

N-(3-methyl-1-phenylbutan-2-yl)acetamide (**4a**) ^1^H-NMR: 0.93 (d, *J* = 6.8, 3H, CH_3_), 0.97 (d, *J* = 6.8, 3H, CH_3_), 1.89 (s, 3H, COCH_3_), 2.67 (dd, *J* = 13.9, 8.1, 1H, CH_2_), 2.84 (dd, *J* = 14.2, 5.9, 1H, CH_2_), 4.09–4.14 (m, 1H, CH-NH), 5.38 (broad s, 1H, NH), 7.17–7.21 (m, 3H, Ar), 7.26 = 7.29 (m, 2H, Ar); ^13^C-NMR: 169.7 (C=O), 138.5 (Ar), 129.1 (Ar), 128.4 (Ar), 126.3 (Ar), 55.1 (CHNH), 38.0 (CH_2_), 30.6 (CH(CH_3_)_2_), 23.4 (COCH_3_), 19.6 (CH(CH_3_)_2_), 17.6 (CH(CH_3_)_2_); IR(KBr) ν_max_, cm^−1^: 3322 ν(N–H, trans), 3084, 3068, 3021 ν(C-H, Ph), 2963 ν^as^(C–H, CH_3_), 2931 ν^as^(C–H, CH_2_), 2869 ν^s^(C–H, CH_3_), 1637 ν(C=O, amide I band), 1541 (δN-H + νC-N, amide II band), 1604, 1518 ν(C=C, Ph), 1469 δ(CH_2_), 1455 δ^as^(CH_3_), 1373 δ^s^(CH_3_); HRMS (ESI) *m/z* 206.15382.

N-(3-methyl-1-phenylbutan-2-yl)benzamide (**4b**) ^1^H-NMR: 1.00 (d, *J* = 6.4, 3H, CH_3_), 1.04 (d, *J* = 6.4, 3H, CH_3_), 1.86–1.92 (m, 1H, CH(CH_3_)_2_), 2.83 (dd, *J* = 14.2, 7.8, 1H, CH_2_), 2.93–2.97 (m, 1H, CH_2_), 4.32 (ddt, *J* = 9.3, 7.9, 6.1, 1H, CHNH), 5.93 (d, *J* = 8.8, 1H, NH), 7.17–7.29 (m, 5H, Ar), 7.36–7.39 (m, 2H, Ar), 7.44–7.47 (m, 1H, Ar), 7.62–7.63 (m, 2H, Ar); ^13^C-NMR: 167.3 (C=O), 138.3 (Ar), 135.1 (Ar), 131.2 (Ar), 129.2 (Ar), 128.5 (Ar), 128.47 (Ar), 126.7 (Ar), 126.4 (Ar), 55.5 (CHNH), 37.98 (CH_2_), 30.8 (CH(CH_3_)_2_), 19.7 (CH(CH_3_)_2_), 17.8 (CH(CH_3_)_2_); IR(KBr) ν_max_, cm^−1^: 3314 ν(N–H, trans), 3084, 3060, 3029 ν(C-H, Ph), 2961 ν^as^(C–H, CH_3_), 2933 ν^as^(C–H, CH_2_), 2873 ν^s^(C–H, CH_3_), 1633 ν(C=O, amide I band), 1578 (δN-H + νC-N, amide II band), 1602, 1578 ν(C=C, Ph), 1492 δ(CH_2_), 1469 δ(CH_2_), 1385 δ^s^(CH_3_); HRMS (ESI) *m/z* 268.16917.

2-Chloro-N-(3-methyl-1-phenylbutan-2-yl)benzamide (**4c**) 0.95 (d, *J* = 7.0, 3H, CH_3_), 0.99 (d, *J* = 6.5, 3H, CH_3_), 1.83 (dq, *J* = 12, 6.8, 1H, CH(CH_3_)_2_), 2.71 (dd, *J* = 14.1, 8.3, 1H, CH_2_), 2.88 (dd, *J* = 14.1, 5.9, 1H, CH_2_), 4.28–4.33 (m, 1H, CHNH), 5.85 (d, *J* = 8.8, 1H, NH), 7.14–7.19 (m, 4H, Ar), 7.21–7.24 (m, 3H, Ar), 7.27–7.28 (m, 2H, Ar); ^13^C-NMR: 166.3 (C=O), 138.4 (Ar), 135.8 (Ar), 130.99 (Ar), 130.1 (Ar), 129.8 (Ar), 129.2 (Ar), 126.99 (Ar), 126.4 (Ar), 55.66 (CHNH), 38.2 (CH_2_), 30.86 (CH(CH_3_)_2_), 19.8 (CH(CH_3_)_2_), 17.6 (CH(CH_3_)_2_); IR(KBr) ν_max_, cm^−1^: 3272 ν(N–H, trans), 3086, 3064, 3031 ν(C-H, Ph), 2958 ν^as^(C–H, CH_3_), 2925 ν^as^(C–H, CH_2_), 2874 ν^s^(C–H, CH_3_), 1689 ν(C=O, amide I band), 1545 (δN-H + νC-N, amide II band), 1496 ν(C=C, Ph), 1473 δ(CH_2_), 1469 δ(CH_2_), 1370 δ^s^(CH_3_), 748 γ(C-H, Ph); HRMS (ESI) *m/z* 302.13018.

N-(3-methyl-1-phenylbutan-2-yl)-2-phenylacetamide (**4d**): 0.79 (d, *J* = 6.8, 3H, CH_3_), 0.89 (d, *J* = 6.4, 3H, CH_3_), 1.65–1.72 (m, 1H, CH(CH_3_)_2_), 2.51 (dd, *J* = 14.2, 8.3, 1H, CH_2_), 2.76 (dd, *J* = 14.2, 5.9, 1H, CH_2_), 4.05–4.10 (m, 1H, CHNH), 5.10 (d, *J* = 8.8, 1H, NH), 7.02–7.03 (m, 2H, Ar), 7.09–7.11 (m, 2H, Ar), 7.17–7.20 (m, 1H, Ar), 7.23–7.25 (m, 2H, Ar), 7.28–7.84 (m, 3H, Ar); ^13^C-NMR: 170.4 (C=O), 138.2 (Ar), 134.95 (Ar), 129.47 (Ar), 129.1 (Ar), 128.35 (Ar), 127.3 (Ar), 126.28 (Ar), 55.05 (CHNH), 43.99 (CH_2_), 37.8 (CH_2_), 30.77 (CH(CH_3_)_2_), 19.4 (CH(CH_3_)_2_), 17.6 (CH(CH_3_)_2_); IR(KBr) ν_max_, cm^−1^: 3285 ν(N–H, trans), 3084, 3064, 3027 ν(C-H, Ph), 2963 ν^as^(C–H, CH_3_), 2910 ν^as^(C–H, CH_2_), 2869 ν^s^(C–H, CH_3_), 1645 ν(C=O, amide I band), 1545 (δN-H + νC-N, amide II band), 1572 ν(C=C, Ph), 1496 δ(CH_2_), 1385 δ^s^(CH_3_); HRMS (ESI) *m/z* 282.18485.

### 2.3. In Silico Pharmacokinetic Profiling and Toxicity Analysis

#### 2.3.1. Theoretical Prediction of Pharmacokinetic Parameters (ADME)

Physicochemical properties, drug-likeness, and pharmacokinetic parameters such as ADME (absorption, distribution, metabolism, elimination) of the synthesized prodrugs were analyzed using SwissADME. It provides a predictive model for the pharmacokinetic profiling of a drug-like compound [22].

#### 2.3.2. Theoretical Prediction of Toxicity

For predicting acute as well as organ toxicity of the compounds, the ProToxII web tool was used. It predicts various toxicity endpoints, including acute toxicity and organ toxicities such as hepatotoxicity, cytotoxicity, carcinogenicity, mutagenicity, immunotoxicity, and toxicity targets. Toxicity class and LD_50_ values were also estimated [23,24].

#### 2.3.3. PASS Online Predictions

PASS online (Prediction of Activity Spectra for Substances), a computer-based program, was used to screen biological activity of the compounds. The program predicts several thousand different biological activities based on the structural formula of a drug-like organic compound [25]. PASS has been used by many scientists for the discovery of new pharmaceutical agents in different therapeutic fields [26,27].

### 2.4. Microbiological Tests

#### 2.4.1. Tested Microorganisms

Eighteen tested microorganisms including five Gram-positive bacteria (*Bacillus subtilis* ATCC 6633, *Bacillus amyloliquefaciens* 4BCL-YT, *Staphylococcus aureus* ATCC 25923, *Listeria monocytogenes* NBIMCC 8632, *Enterococcus faecalis* ATCC 19433), five Gram-negative bacteria (*Salmonella enteritidis* ATCC 13076, *Klebsiella* sp.—clinical isolate, *Escherichia coli* ATCC 25922, *Proteus vulgaris* ATCC 6380, *Pseudomonas aeruginosa* ATCC 9027), two yeasts (*Candida albicans* NBIMCC 74, *Saccharomyces cerevisiae* ATCC 9763) and six fungi (*Aspergillus niger* ATCC 1015, *Aspergillus flavus*, *Penicillium* sp., *Rhizopus* sp., *Mucor* sp.—plant isolates, *Fusarium moniliforme* ATCC 38932) from the collection of the Department of Microbiology at the University of Food Technologies—Plovdiv, Bulgaria, were selected for the antimicrobial activity test.

#### 2.4.2. Culture Media

- Luria-Bertani agar medium supplemented with glucose (LBG agar)

LBG agar was prepared by the manufacturer’s (Laboratorios Conda S.A., Madrid, Spain) prescription: 50 g of LBG-solid substance mixture (containing 10 g tryptone, 5 g yeast extract, 10 g NaCl, 10 g glucose and 15 g agar) was dissolved in 1 L of deionized water (pH 7.5), and then the medium was autoclaved at 121 °C for 20 min.

- Malt extract agar (MEA)

MEA was prepared by the manufacturer’s (Laboratorios Conda S.A., Madrid, Spain) prescription: 50 g of the MEA-solid substance mixture (containing 30 g malt extract, 5 g mycological peptone, and 15 g agar) was dissolved in 1 L of deionized water (pH 5.4), and then the medium was autoclaved at 115 °C for 15 min.

#### 2.4.3. Antimicrobial Activity Assay

The antimicrobial activity of the samples was determined by the agar well diffusion method. The tested bacteria *B. subtilis* and *B. amyloliquefaciens* were cultured on LBG agar at 30 °C. The test bacteria *S. aureus*, *L. monocytogenes*, *E. faecalis*, *S. enteritidis*, *Klebsiella* sp., *E. coli*, *P. vulgaris* and *P. aeruginosa* were cultured on LBG agar at 37 °C for 24 h. The yeast *C. albicans* was cultured on MEA at 37 °C, while *S. cerevisiae* was cultured on MEA at 30 °C for 24 h. The fungi *A. niger*, *A. flavus*, *Penicillium* sp., *Rhizopus* sp., *Mucor* sp., and *F. moniliforme* were grown on MEA at 30 °C for 7 days or until sporulation.

The inocula of the tested bacteria/yeasts were prepared by homogenization of a small amount of biomass in 5 mL of sterile 0.5% NaCl. The inocula of tested fungi were prepared by the addition of 5 mL of sterile 0.5% NaCl into the tubes. After stirring by vortex V-1 plus (Biosan), they were filtered and replaced in other tubes before use. The number of viable cells and fungal spores was determined using a bacterial counting chamber Thoma (Poly-Optik, GmbH, Bad Blankenburg, Germany). Their final concentrations were adjusted to 10^8^ cfu/mL for bacterial/yeast cells and 10^5^ cfu/mL for fungal spores and then inoculated in preliminarily melted and tempered at 45–48 °C LBG/MEA agar media. Next, the inoculated media were transferred in a quantity of 18 mL in sterile Petri plates (d = 90 mm) (Gosselin™, Hazebrouck, France) and allowed to harden. Then, six wells (d = 6 mm) per plate were cut, and triplicates of 60 μL of each extract were pipetted into the agar wells. The Petri plates were incubated in identical conditions.

The antimicrobial activity was determined by measuring the diameter of the inhibition zones around the wells on the 24th and 48th h of incubation. Tested microorganisms with inhibition zones of 18 mm or more were considered sensitive; moderately sensitive were those in which the zones were from 12 to 18 mm; resistant were those in which the inhibition zones were up to 12 mm or completely missing [28].

### 2.5. Cytotoxic Activity

In order to evaluate the in vitro biocompatibility of the experimental compounds, a series of cell viability assays were performed against human malignant leukemic cell lines (LAMA-84, K-562) as well as normal murine fibroblast cells (CCL-1). All cell lines were purchased from the German Collection of Microorganisms and Cell Cultures (DSMZ GmbH, Braunschweig, Germany). Cell cultures were cultivated in a growth medium RPMI 1640 supplemented with 10% fetal bovine serum (FBS), 5% L-glutamine, and incubated under standard conditions of 37 °C and 5% humidified CO_2_ atmosphere.

#### Cell Viability Assay

The experimental design involved a number of cytotoxicity assays that measured cell growth inhibition by the newly synthesized compounds. Cell viability was evaluated using a standard MTT-based colorimetric assay. Exponential-phased cells were harvested and seeded (100 μL/well) in 96-well plates at the appropriate density (3 × 10^5^) for the suspension cultures (LAMA-84 and K-562) and 1.5 × 10^5^ for the adherent one (CCL-1). Cells were treated and incubated with various concentrations of the experimental compounds in the concentration range of 400-6.25 µM. After exposure time of 72 h, a filter-sterilized MTT substrate solution (5 mg/mL in PBS) was added to each well of the culture plate. A further 1–4 h incubation allowed for the formation of purple insoluble formazan precipitates. The latter were dissolved in isopropyl alcohol solution containing 5% formic acid prior to absorbance measurement at 550 nm using a microplate reader (Labexim LMR-1). Collected absorbance values were blanked against MTT and isopropanol solution and normalized to the mean value of the untreated control (100% cell viability). Semi-logarithmic “dose–response” curves were constructed and the half-inhibitory concentrations of the screened compounds against each tested cell line were calculated. Values of *p* ≤ 0.05 were considered statistically significant.

### 2.6. Bioelectrical Activity

The experiments were approved by the Ethical Committee of the Bulgarian Food Agency with No. 229/09.04.2019 and were carried out following the guidelines of the European Directive 2010/63/EU. The animals were provided by the Animal House of the Medical University of Plovdiv, Bulgaria.

Adult male Wistar rats weighing 280–300 g were used. Animals were housed in standard laboratory conditions (23 to 25 °C, 50–55% humidity, 12/12 h light/dark cycle), while food and water were provided ad libitum.

Circular SM preparations were dissected from the corpus gastric muscle (26–28 mm long and 0.8–0.9 mm wide; without mucosa). Two or three muscle strips were taken from the rat stomach. The number of SM preparations used for each data point is indicated by n. After dissection, the tissues must prevent loss of viability, so during the dissection, the tissues were systematically moistened with a solution (NaCl:KCl:CaCl_2_ in ratio 27.2:1.1:1) prepared at 4 °C.

The BEA of SM preparations was investigated by means of the single sucrose gap method with silver non-polarizable freshly chlorinated electrodes. The muscle strips were divided into 3 zones: two electrode sections were flushed with isotonic KCl solution and Krebs solution (pH 7.3), respectively, and separated by a sucrose layer of high electric resistance. Krebs contained the following (in mM): NaCl 120; KCl 5.9; CaCl_2_ 2.5; MgCl_2_ 1.2; NaH_2_PO_4_ 1.2; NaHCO_3_ 15.4; glucose 11.5. All the ingredients for this solution were of analytical grade and obtained from Merck (Darmstadt, Germany). Deionized water (18.2 mX/cm^2^) was used thoroughly. The KCl solution is similar to the Krebs solution but with an equimolar substitution of NaCl with KCl. An isotonic sucrose solution, due to its very high specific resistance (about 10^6^ Ω·cm), provides the best possible separation of the sucrose solution from the physiological solution in the “normal” pool, on the one hand, and the solution providing a zero value of the membrane potential in the pool with another recording electrode.

The inflow rate of each solution (Krebs, KCl, and isotonic sucrose solution) was controlled by a needle valve and continuously aerated with a mixture of 95% O_2_ and 5% CO_2_, and the temperature was maintained at 37 °C. A potential difference between the two end sections of the gap (control conditions) was achieved by means of silver non-polarizable, freshly chlorinated electrodes and after a 40-min period of adaptation and stabilization was accepted as the initial (control). The Krebs solution was changed several times during the adaptation period. The viability of the SM preparations was periodically tested by treatment with 1 × 10^−6^ M ACh.

The membrane potential value was visualized by means of a Linseis recorder (Selb, Germany). A pair of measuring electrodes is located on both sides of the sucrose sections. The pH (pH 7.3; 37 °C) of the Krebs bathing solution was measured before each experiment by pH-meter HI5521 (Hanna Instruments Inc., Woonsocket, RI, USA).

The CAs were recorded from the fragments of the SM preparations located in the section with Krebs solution. The mechanical changes were registered isometrically with a Microtechna amplifier (Prague, Czech Republic) and recorded by a Linseisrecorder (Selb, Germany).

The new substances were dissolved initially in dimethyl sulphoxide (DMSO) to prepare a 5 × 10^−2^ M stock solution. Further dilutions were made in distilled deionized water and were applied by adding aliquots of their concentrated solutions to the tissue bath. The final concentration of the studied substances in the tissue baths did not exceed 1:100.

### 2.7. Immunohistochemical Analysis

#### 2.7.1. Histology

After dissection, the SM strips were incubated for 20 min in the tissue bath using the same physiological conditions in which the CA recording experiments were carried out. Then, each SM strip was fixed in 10% neutral buffer formalin for 24–48 h and submitted for standard processing with hematoxylin and eosin staining.

The SM fragments from a rat’s gastric wall were fixed in a 10% neutral formalin solution and embedded in paraffin. Paraffin sections of 5 μm thickness were subjected to hematoxylin and eosin (H-E) staining for histochemical analysis.

#### 2.7.2. Hematoxylin-Eosin Staining

The sections were stained with Mayer’s hematoxylin (5 min) and differentiated in tap water (20 min). After completion of the differentiation, the sections were stained with water-soluble eosin for 5 min. The subsequent stained sections were dehydrated again in ascending grades of ethanol (70%, 95%, 100%), cleared in xylene (2 × 10 min), and covered with Canadian balm.

#### 2.7.3. Immunohistochemistry

Sections of the SM fragments from the wall of a rat’s stomach with a thickness of 5 µm were deparaffinized, then subjected to antigenic detection of the epitopes with citrate buffer, and an endogenous peroxidase blockade was made with hydrogen peroxidase 3%, a kit was used to block the endogenous biotin, and a reagent was used to block non-specific binding (Superblock, Scy Tek, Santa Cruz Biotech. Inc. Heidelberg, Germany) follows incubate for 24 h (at 4 °C) with monoclonal mouse anti-5-HT_3_, next incubated with a secondary antibody: biotinylated anti-rabbit for 10 min at room temperature. The reaction was visualized with 3,3′-diaminobenzidine tetrachloride and counterstained with Mayer’s hematoxylin. The preparations were observed with a light microscope at magnification levels of ×200.

#### 2.7.4. Quantitative Analysis of Immunohistochemical Reactions

A quantitative and statistical analysis of immunohistochemical reaction using the Olympus DP-Soft image system (version 4.1 for Windows) was carried out on a Microphot-SA (Nikon, Japan) microscope equipped with a Camedia-5050 Z digital camera (Olympus, Japan). The analysis was performed on sections from the SM strips from the stomach of Wistar rats (*n* = 6 for each group). Five sections of the SM strips were measured, and the percentage of cells expressing 5-HT_3_ in the circular and longitudinal layer of SM cells, as well as in the myenteric plexus of the stomach, was determined. Each antibody was analyzed for five fields, in each of them the average number of cells with positive unit area response at ×200 magnification.

### 2.8. Statistical Analysis

All data were expressed as mean values ± standard error of the mean (mean ± SEM); the number of muscle preparations used for each data point was indicated by n. The results were analyzed by analysis of variance (ANOVA) to find the values that showed significant difference (*p* < 0.05). INSTAT computer program (GraphPad Software, Inc., San Diego, CA, USA) was applied for the analysis of experimental data.

## 3. Results and Discussion

Investigating the biological activity of the newly synthesized compounds is a relevant scientific matter due to the interest in the drug discovery area. In our previous reports, we discussed the synthesis and SM BEA for different compounds, such as 1,2,3,4-tetrahydroisoquinolines [29], isoquinoline precursors [6], melanoidins [30,31], eucalyptol, thymol [32,33], etc.

### 3.1. In Silico Predictions and Synthesis

To be effective as a drug, a given molecule must reach its pharmacological target in the body, achieve an adequate concentration at the site of action, and persist in a bioactive form long enough for the expected biological events to occur. Many compounds failed as drugs because of their poor pharmacokinetics properties and limited bioavailability. The large number of newly synthesized experimental molecules, on one hand, and the quantitative limitations of tissue samples, together with the need to restrict animal testing, on the other, prevent systematic recourse to experiments. In this context, in silico computer models constitute a valid alternative and a useful addition to biological experiments [34]. In our calculations, the PASS Online Program (Prediction of Activity Spectra for Substances) was used. The latter predicted the potential spasmolytic activity of the tested compounds based on a papaverine-like structure, and their modulatory effect on serotonin kinetics (release and uptake of the mediator) and its receptor transmission.

**Scheme 1:**
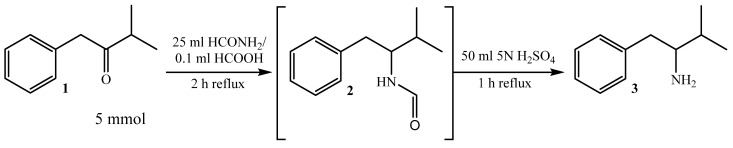
Reaction pathway for the synthesis of 3-methyl-1-phenylbutan-2-amine **3**.

Our strategy was based on the synthesis of 3-methyl-1-phenylbutan-2-amine from a starting ketone and its acylation with acid halides to prepare corresponding amides. To obtain the compounds, we successfully used a previously described protocol [6] using 3-methyl-1-phenylbutan-2-one **1** as a starting compound. The ketone applied in Leuckart reaction with HCOOH and HCONH_2_ and reflux for 2 h at 180 °C produced formamide **2** which after hydrolysis in 5N H_2_SO_4_ obtained the target amine **3** with 80% yield, according to the following Scheme 1.

Due to the importance of their synthesis as isoquinoline precursors and antispasmodics, a target amine **3** and its new amides were successfully achieved in order to investigate their biological activity. The synthesis of amides is extremely important due to the high presence of this group in biological systems, as well as its key role in medicinal chemistry, where one of the most common chemical reactions is amide synthesis. Amide groups are also synthetically versatile, capable of taking part in a wide range of different transformations. It is difficult to overstate the importance of the amide functional group in molecular sciences. This motif is of crucial importance to form the backbone of peptides, proteins, and other biomolecules [35]; the amide-forming reactions being the most commonly carried out in the pharmaceutical industry [36]. Approximately a quarter of all marketed drugs and many of all drug candidates bear an amide bond [37]. Amide interactions with biological targets are another key aspect to be considered in drug discovery [38].

**Scheme 2:**
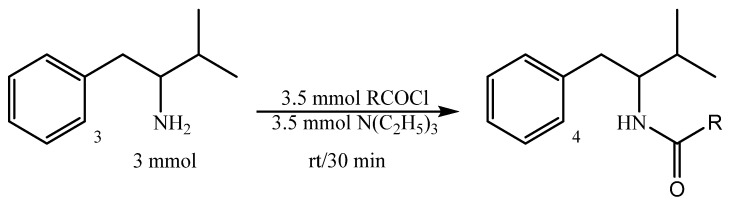
Synthesis of N-(1-(3,4-dimethoxyphenyl)propan-2-yl) amides **4**.

Based on the in silico results, we worked on a synthetic route to apply different acyl chlorides, bearing electron-donating and electron-withdrawing groups as the starting material (Scheme 2, Table 1).

In general, reactions with different acyl chlorides proceeded efficiently, furnishing the target amides **4a**–**d** in 69–83% yield (Table 1). Comparing the yield with previously reported compounds having a 3-methyl group [6], we can conclude that the yield for 3-isopropyl substituted compounds has decreased because of the lack of methoxy groups in the benzene ring. The resultant compounds are characterized by their melting point, IR, ^1^H, ^13^C-NMR, and HRESIMS spectra. Spectral data confirmed the structure of all the obtained compounds (Supplementary Materials Figures S1–S20).

The blood–brain barrier (BBB) and gastrointestinal absorption are pharmacokinetics characteristics that play an essential role in the drug discovery process. In order to elucidate the ADME profile of the compounds, free SwissADME web tools were used (Table 2).

Molecular weight (MW) and octanol/water partition coefficient (XLOGP3) are the drug-likeliness parameters, known as Lipinski’s rule of five [39]. According to the rule, at least two parameters from four basic pharmacokinetic properties (MW ≤ 500; XLOGP3 ≤ 5; the number of hydrogen bond donors ≤ 5; and hydrogen bond acceptors ≤ 10.6) should be fulfilled for drug candidates. Bioavailability (BA) [40] reported an optimal range of distinct properties involving lipophilicity (XLOGP3: −0.7 to +5.0), size (MW: 150 to 500 g/mole), polarity (TPSA: 20 to 130 Å^2^), ESOL or estimated solubility (log S: not more than 6), saturation (Fraction Csp^3^ or fraction of carbons in the sp^3^ hybridization: not less than 0.25), and flexibility (RB: no more than 9).

The ADME investigation shows that the compounds cross through the BBB and present good gastrointestinal absorption and could be considered as a drug candidate according to Lipinski’s rule of five [39], an important step in a drug discovery process. The calculated TPSA values of the compounds are in the range of 26.02–29.10 Å^2^, indicating good intestinal absorption and BBB penetration. The low number of rotatable bonds (**3**, Table 2) for **3** and **4a** compounds corresponds with sufficient oral bioavailability.

P-glycoproteins are pharmacokinetic proteinsfunctioning as xenobiotic neuroprotectors, drugs, etc. [41,42] According to the calculation, the studied compounds are not predicted as P-glycoprotein substrates. It is known that inhibition of cytochrome P450 (CYP) could lead to toxicity or lack of drug efficacy [43,44]. According to the calculations, all the compounds are expected to inhibit the CYP2D6 isoform. Compounds **4c** and **4d** with a phenyl ring can inhibit the CYP1A2 and CYP2C19, while chlorine containing **4d**—the CYP2C9. Compounds **3** and **4a** have the lowest skin permeability with log Kp −5.46 and -5.50. The log Kp coefficients of **4b** and **4c** are between −4.46 and −4.83.

The solubility parameter is very important for the pharmaceutical applications of the drug. All drugs were predicted by SwissADME to be water-soluble or moderately soluble in water. The values of the drug-likeness parameters of all compounds were found to remain within the BA, which confirmed that all the synthesized compounds behave as “drug-like” substances.

All of the compounds were calculated to have a BA score of 0.55, which demonstrates good oral bioavailability, according to Lipinski’s rule [39]. Moreover, they have good synthetic accessibility (SA) scores, which are considerable parameters during the drug discovery processes. The predicted LD_50_ for all the compounds is between 241 mg/kg and 2500 mg/kg.

The newly synthesized compounds were then subjected to an in vitro evaluation of their antimicrobial and cytotoxic activity against human malignant leukemic cell lines (LAMA-84, K-562) as well as normal murine fibroblast cells (CCL-1).

### 3.2. Antimicrobial Activity

Biofilms are aggregated bacteria attached to surfaces and embedded in a self-produced matrix of extracellular polymeric substances. The local environment within a biofilm offers protection to persister cells from the immune system [45]. These bacterial biofilms can be formed on any surface, including medical implants or water distribution systems in hospitals [46]. Bacteria, especially in biofilm, can be extremely difficult to eradicate and cause therapeutic problems, as is the case with the critical pathogen *P. aeruginosa*. Antibiotic resistance is one of the most persistent issues worldwide nowadays and many powerful antibiotics have failed in controlling infections. Recently, Krishnan and Kandasamy investigated the possibility of using the antispasmodic mebeverine against methicillin-resistant *Staphylococcus aureus.* They proved mebeverine to be favorable in the antibiotics dose reduction, and cutting down the need for additional administration of antibiotics to the patients affected with multiple complications such as gastrointestinal ulcer, spasm difficulties, and infection [47,48,49].

In our experiments, five Gram-positive bacteria, five Gram-negative bacteria, two yeasts, and six fungi were used. All the mebeverine precursors were tested in vitro for their antimicrobial activity against human pathogenic bacteria and economically relevant phytopathogenic fungi. The inhibition zones of bacterial and fungal growth caused by the novel compounds are outlined in Table 3. The methanol used as a solvent for the samples did not show any antimicrobial effect.

We observed that the amine **3** derivative exerted only modest activity against Gram-negative bacteria including the most pathogenic *Klebsiella* sp., *Pseudomonas aeruginosa*, as well as against *Saccharomyces cerevisiae*. The amide **4a,** on the other hand, inhibited only the growth of *Escherichia coli*. None of the other amides exhibited any antimicrobial activity. This trend led us to the conclusion that antibacterial properties are distinctive to the amine and less substituted amides, such as acetyl, whereas amides with bulkier phenyls or benzyl group substituents did not show any antimicrobial activity. The most important conclusion in our experiments is that mebeverine did not show any antimicrobial activity in the same concentration range. This gives us an opportunity to further investigate biological activities of the synthesized more potent mebeverine precursors.

### 3.3. Cytotoxic Activity

A series of MTT experiments were conducted against normal murine fibroblast cells and malignant human cell lines of leukemic origin to accommodate the cytotoxicity of the target compounds. The results of the antiproliferative assays are presented in Table 4. According to our study, cell growth was unaffected following 72 h exposure to various treatment concentrations of the compounds in all the screened cell lines. Calculated IC_50_ values range in the 240–360 µM for the leukemic models and lie strictly above 300 µM in the normal fibroblast cells. The compounds induced no morphological changes under microscopic examination and were practically devoid of any intrinsic cytotoxic activity in the tested treatment concentrations that many times exceed their spasmolytic (5 × 10^−5^ M) dose.

Based on the PASS online and the ProToxII in silico calculations, the evaluation of the cytotoxic activity of the compounds was the other object of our investigation. We found that the compounds induced no morphological changes under microscopic examination and were practically devoid of any intrinsic cytotoxic activity in the tested treatment concentrations. Furthermore, they showed a more favourable cytotoxicity profile as compared to the spasmolytic drug used as reference (mebevirine), whose half-inhibitory concentrations on the malignant cell lines LAMA-84 and K-562 fall just under the hundred-micromolar range (Table 4).

### 3.4. Ex Vivo Effects of 3-Methyl-1-phenylbutan-2-amine ***3*** and Its Amides ***4a***–***d*** on the Spontaneous Bioelectric Activity

Taking into account the potential effect of the compounds on muscle tonus, the spontaneous BEA on SM isolated from the rat stomach was established. The response of a living cell to various stimuli, whether of an excitation origin producing a bioelectrical response or of a chemical nature, always occurs after some latent lag period. This time interval (meantime) is relatively short in the cells of nerve and muscle tissue, which are adapted to carry out rapid responses to irritation. In SMs, the excitation process is associated with changes in BEA, because the sarcolemma of most of them has potential-dependent ion channels and, for this reason, belongs to the electro-excitable membranes. Gastric SMs are spontaneously active and generate rhythmic activities such as slow waves or action potentials, or both [50]. The membrane potential of SM cells has a relatively low value of −30 mV to −65 mV. Its absolute value with a suprathreshold electrical or chemical stimulus reaches the threshold of the opening of the potential-dependent Ca^2+^ channels and the entry of calcium into the sarcolemma [51]. The increased amount of Ca^2+^ in the cytosol causes the generation of the depolarization phase of the spike potentials in the cells, which cause phasic contractions of the muscle cells [50]. When intracellular recordings are made from the SM preparation, three types of electrical responses are found: pacemaker potentials, slow waves, and follower potentials [52]. In our experiments, the mean response time of SM cells was in the order of 10 min for the complete change in slow wave character to develop. Membrane potential value was significantly increased by 55% after the addition of **3** into the tissue bath. That probably depends on the activation of voltage-gated Ca^2+^ ion channels in which the Ca^2+^ influx is observed.

**Figure 4:**
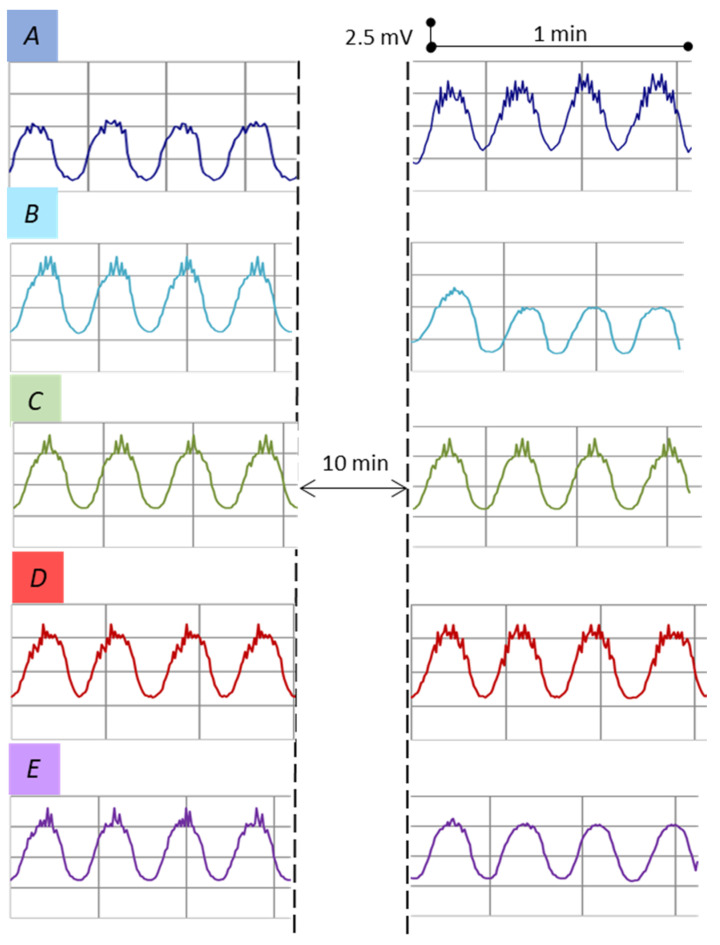
Experimental record of bioelectric activity of SM preparations, registered through sucrose gap method—control and 10 min after influence of 5 × 10^−5^ M **3** (**A**), **4a** (**B**), **4b** (**C**), **4c** (**D**), **4d** (**E**).

Affecting this functionally important process is also confirmed by the significant increase in the frequency of spike potentials (Figure 4; Table 5), responsible for SM contractions [53].

Relaxation of smooth muscle occurs by two mechanisms: a hyperpolarization of the membrane through activation of K_ATP_ channels and elevation of intracellular cGMP levels due to its role as a nitric oxide (NO) donor [54]. Hyperpolarization of the membrane has two distinct actions on slow waves: reduction in the amplitude and a shift in the threshold potential to more negative levels [55,56].

Our experiments with single sucrose gap method [57] showed a reduction in the frequency and amplitude of spike potentials generated by SM preparations in the presence of the amide **4d** (Figure 4E). Spike potentials are known to be a product of Ca^2+^ currents through L-type membrane Ca^2+^ channels and the significant reduction in their indicators under the influence of **4d** is an indication of the probable participation of this type of membrane ion channels in the bioelectric activity of the studied substances.

We found that exogenous administration of **4a** unequivocally demonstrated increased reactivity in SM rat stomach preparations. We registered a complete disappearance of the spike potentials responsible for SM contractility. The biological activity of amide **4a** is also confirmed by hyper-polarization, expressed in a reliable shift of the value of the membrane potential to more negative values (Figure 4B).

The BEA of SM preparations for substances **4b** and **4c** was also investigated. The data do not demonstrate any changes in their slow waves (Figure 4C,D). Based on the experiments, we can assume that **4b** and **4c** do not affect the BEA of the isolated tissues studied by the single sucrose gap method.

We also accepted the possibility of modulating the general bioelectrical mechanisms inducing contractility in SM. We assumed that the different nature of the responses to exogenously applied compounds **3**, **4a**, and **4d** as new molecules in their capacity as excitatory substances is due to the specificity in the type of the substituents and their position. In order to analyze the validity of this hypothesis, measurements of possible changes in the BEA of SM preparations for substances **4b** and **4c** were made. The data do not demonstrate any changes in their slow waves. Therefore, we can assume that **4b** and **4c** do not affect the BEA of the isolated tissues studied by the single sucrose gap method.

Which one of the mentioned processes and how is affected by the newly synthesized substances are our next goals to be investigated by tracking isometric changes in the contractile activity of SM preparations, based on the already proven influence on the bioelectrical changes that initiate them.

### 3.5. Immunoreactivity for the 5-HT_3_ Receptor

**Figure 5:**
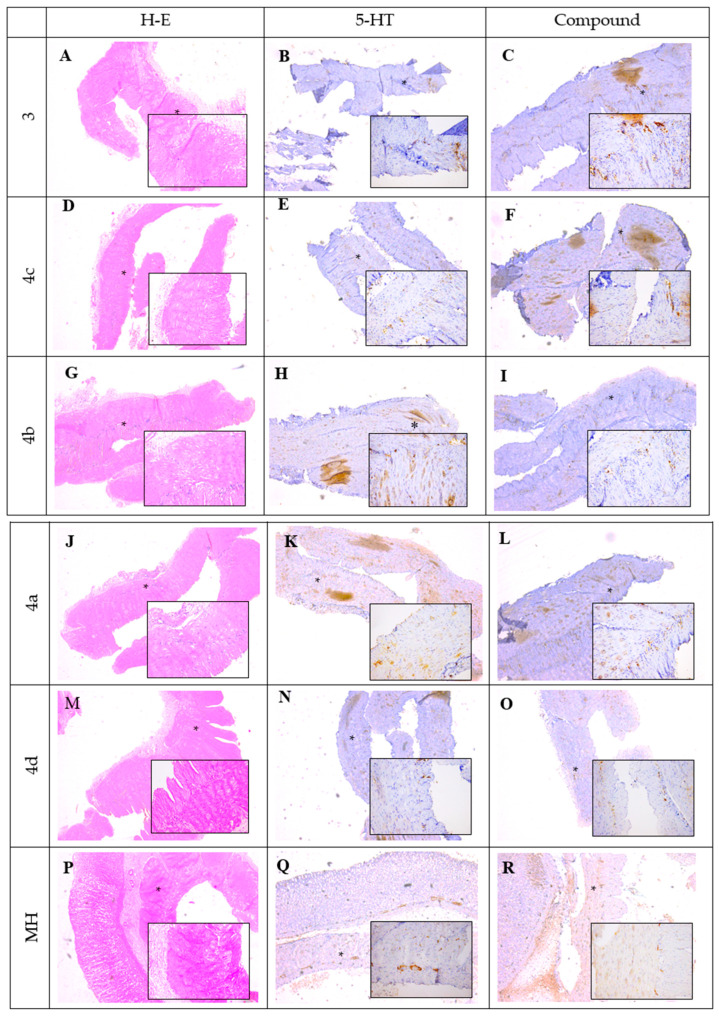
Histological and immunohistochemical staining of SM preparations after 1 h incubation period. (**A**,**D**,**G**,**J**,**M**,**P**) were incubated with **3**, **4a**–**d** and mebeverine hydrochloride (MH), H-E staining, ×5, ×20; (**B**,**E**,**H**,**K**,**N**,**Q**) were incubated with 5-HT, 5-HT3 expression observed in the myenteric plexus (black stars), ×5, ×20; (**C**) was incubated with **3**, with increased intensity in 5-HT3 expression in myenteric plexus observed (black stars), ×5, ×20; (**F**) was incubated with **4c**, with no 5-HT3 expression observed, ×5, ×20; (**I**) was incubated with **4b**, with no 5-HT3 expression observed, ×5, ×20; (**L**) incubated was incubated with **4a**, with no 5-HT3 expression observed, ×5, ×20; (**O**) was incubated with **4d**, with weak 5-HT3 expression observed (black stars), ×5, ×20; and (**R**) was incubated with MH, with weak 5-HT3 expression observed (black stars), ×5, ×20.

Finally, a semiquantitative analysis of the immunohistological appearance of the SM preparations incubated with 5-HT and the newly synthesized substances was performed (Figure 5). Analysis of the results of 5-HT_3_ receptors expression in gastric SM strips showed a higher number of 5-HT_3_R positive cells per section when treated with compound **3** or 5-HT separately. In the preparations incubated with the newly synthesized compounds **4a**, **4b**, and **4c**, no modulation on the 5-HT_3_R expression was observed. Preparations incubated with compound **4d** showed a very weak non-specific effect on the serotoninergic 5-HT_3_R.

The results of the immunohistological analysis showed that amine **3** has the ability to induce the expression of 5-HT_3_ receptors on afferent neurons and nerve fibers in the Myenteric plexus of SM preparations (Table 6). Administration of **3** activates 5-HT_3_ receptors, possibly causing rapid inward currents (or membrane depolarization) in neurons consistent with 5-HT_3_R function as a ligand-gated cation channel. This suggests that amine **3** has the potential to modulate afferent neuronal activity at distinct sites of the gastrointestinal tract and may therefore prolong or enhance local GIT signaling.

## 4. Conclusions

In conclusion, a new amine and a series of its amide derivatives were synthesized, as mebeverine precursors and antispasmodics. Generally, all the synthesized compounds showed favourable predicted in silico profiles against the majority of the selected descriptors. In silico data signified amides as potential orally active medicinal substances with reduced toxicity. Only amine **3** demonstrated modest antimicrobial activity against Gram-negative bacteria *Klebsiella* sp., *Pseudomonas aeruginosa*, and yeasts *Saccharomyces cerevisiae*. We can conclude that the antimicrobial activity of the amine was higher than the mebeverine activity in the same concentration range. Overall, no cytotoxicity to human malignant leukemic cell lines (LAMA-84, K-562) was observed for all tested compounds. We established ex vivo BEA using functionally active isolated tissues. The results showed that three of the synthesized substances **3**, **4a**, and **4d** exhibit BEA, expressed in a reliable change of the main characteristics of the slow wave—the value of the membrane potential, the frequency, and amplitude of the wave, and the spike potentials. Furthermore, the immunohistochemical analysis indicated that amine **3** has the potential to modulate afferent neuronal activity at distinct sites of the gastrointestinal tract and may therefore prolong or enhance local gastrointestinal signaling. 

The summary results of the conducted experiments distinguished the newly synthesized 3-methyl-1-phenylbutan-2-amine **3** as a leader compound with favourable antimicrobial, immunohistochemical and spasmolytic activity, and a reasonable effective choice for orally active long-term therapy of chronic IBS. Thus, further investigation into the possible medicinal applications of this compound is warranted in the future.

## Figures and Tables

**Table 1:** Synthesis of 3-methyl-1-phenylbutan-2-amine.

4	R	Yield, %	mp, °C
**a**	CH_3_	83	97–99
**b**	C_6_H_5_	79	144–145
**c**	2-Cl-C_6_H_4_	79	82–83
**d**	CH_2_-C_6_H_5_	69	86–87

**Table 2:** Calculated ADME parameters of the compounds **3** and **4a**–**d** representing drug-likeness.

Compound	MW, g/mol	XLOGP3	TPSA, Å^2^	ESOL Log S	Fraction Csp^3^	RB	BA Score	SA Score	LD_50_, mg/kg
**3**	163.26	2.59	26.02	−2.66	0.45	3	0.55	1.48	241
**4a**	205.30	2.89	29.10	−2.90	0.46	5	0.55	1.74	899
**4b**	267.37	4.55	29.10	−4.41	0.28	6	0.55	2.08	2000
**4c**	301.81	5.18	29.10	−5.00	0.28	6	0.55	2.38	2500
**4d**	281.39	4.49	29.10	−4.37	0.32	7	0.55	2.29	825

MW: Molecular weight, XLOGP3: Octanol/water partition coefficient, TPSA: Topological polar surface area, ESOL LogS: Estimated aqueous solubility, Fraction Csp^3^: Ratio of sp^3^ hybridized carbons over the total carbon count, RB: Rotatable bonds, BA: Bioavailability, SA: Synthetic accessibility, LD_50_—predicted lethal dose to 50% of animals.

**Table 3:** Antimicrobial activity of the tested compounds.

	Inhibition Zones, mm						
Tested Microorganism/Compound	3	4a	4b	4c	4d	Mebeverine	Methanol
*Klebsiella* sp.	8	-	-	-	-	-	-
*Escherichia coli* ATCC 25922	-	8	-	-	-	-	-
*Pseudomonas aeruginosa* ATCC 9027	9	-	-	-	-	-	-
*Saccharomyces cerevisiae*	8	-	-	-	-	-	-

Dose 0.6 mg/disk, dwell = 6 mm.

**Table 4:** In vitro cytotoxicity of the tested compounds [µM ± SD] against cell lines of different origin.

Compound/Cell Line	LAMA-84 ^a^	K-562 ^b^	CCL-1 ^c^
**3**	239.4 ± 18.2	249.9 ± 10.5	>300
**4a**	359.8 ± 11.0	276.3 ± 19.7	>300
**4b**	265.4 ± 20.2	328.8 ± 20.4	>300
**4c**	265.9 ± 16.8	366.6 ± 8.4	>300
**4d**	328.3 ± 15.9	243.7 ± 18.5	>300
Mebeverine hydrochloride	72 ± 6.2	85.4 ± 8.3	>300

^a^ CML in blast crisis (bcr-abl+); ^b^ Blast phase CML (bcr-abl+); ^c^ normal murine fibroblast cells.

**Table 5:** Changes of some bioelectric parameters of SM preparations registered through sucrose gap method after influence of the newly synthesized mebeverine precursors 5 × 10^−5^ M (n = 7). The asterisk indicates the values that differ significantly (* *p* < 0.05) compared to the respective control parameters; base activity is taken as 100%, ↑ show depolarization; ↓—hyperpolarization.

Bioelectric Activity Parameters	Synthesized Mebeverine Precursors 3 and 4a–d					
		3	4a	4b	4c	4d
Amplitude of slow waves, mV	Autocontrol value	5.15 ± 0.07	4.92 ± 0.18	5.05 ± 0.26	5.23 ± 0.17	5.13 ± 0.28
	Measured value	5.28 ± 0.33	4.32 ± 0.38	5.09 ± 0.10	5.34 ± 0.20	4.97 ± 0.29
Number of slow waves, min^−1^	Autocontrol value	4.33 ± 0.11	4.20 ± 0.09	4.03 ± 0.08	4.12 ± 0.10	3.93 ± 0.07
	Measured value	4.90 ± 0.09	4.72 ± 0.55	4.50 ± 0.49	4.42 ± 0.41	4.82 ± 0.24
Amplitude of spike potentials, mV	Autocontrol value	0.95 ± 0.36	1.3 ± 0.25	1.1 ± 0.20	1.0 ± 0.12	1.2 ± 0.16
	Measured value	2.61 ± 0.16 *	0 *	1.40 ± 0.12	1.56 ± 0.17	0 *
Number of spike potentials min^−1^	Autocontrol value	21.43 ± 0.20	27.12 ± 0.23	20.17 ± 0.50	25.44 ± 0.14	26.79 ± 0.55
	Measured value	48.22 ± 0.39 *	0 *	22.28 ± 0.18	26.16 ± 0.26	0 *
Changes in the membrane potential, %	-	55 ↑	41 ↓	0	0	0

**Table 6:** 5-HT expression and response to compounds **3**, **4a**–**d** compared to mebeverine hydrochloride (MH).

Compound	5-HT	Response to Compound
**3**	++	+++
**4a**	++	−
**4b**	++	−
**4c**	++	−
**4d**	++	+
MH	++	++

The expression of 5-HT_3_ was characterized in a panel of SMs and neurons in the myenteric plexus. The expression level of 5-HT_3_ protein was determined by assessing the antigen expression in tissue sections by immunohistochemical staining, such as: −, absent; +, low; ++, moderate; +++, strong.

## Supplementary Materials

The following supporting information can be downloaded at: https://www.mdpi.com/article/10.3390/biomedicines11020605/s1, Figure S1: ^1^H-NMR spectrum of compound **3**, Figure S2: ^13^C-NMR spectrum of compound **3**, Figure S3: DEPT spectrum of compound **3,** Figure S4: IR spectrum of compound **3**, Figure S5: ^1^H-NMR spectrum of compound **4a**, Figure S6: ^13^C-NMR spectrum of compound **4a**, Figure S7: DEPT spectrum of compound **4a,** Figure S8: IR spectrum of compound **4a**, Figure S9: ^1^H-NMR spectrum of compound **4b**, Figure S10: ^13^C-NMR spectrum of compound **4b**, Figure S11: DEPT spectrum of compound **4b,** Figure S12: IR-spectrum of compound **4b**, Figure S13: ^1^H-NMR spectrum of compound **4c**, Figure S14: ^13^C-NMR spectrum of compound **4c**, Figure S15: DEPT spectrum of compound **4c,** Figure S16: IR spectrum of compound **4c**, Figure S17: ^1^H-NMR spectrum of compound **4d**, Figure S18: ^13^C-NMR spectrum of compound **4d**, Figure S19: DEPT spectrum of compound **4d,** Figure S20: IR spectrum of compound **4d.**
