# Correction: Milusheva et al. In Silico, In Vitro, and Ex Vivo Biological Activity of Some Novel Mebeverine Precursors. *Biomedicines* 2023, *11*, 605

**DOI:** 10.3390/biomedicines13102564

**Published:** 2025-10-21

**Authors:** Miglena Milusheva, Vera Gledacheva, Iliyana Stefanova, Mina Pencheva, Rositsa Mihaylova, Yulian Tumbarski, Paraskev Nedialkov, Emiliya Cherneva, Mina Todorova, Stoyanka Nikolova

**Affiliations:** 1 Department of Bioorganic Chemistry, Faculty of Pharmacy, Medical University of Plovdiv, 4002 Plovdiv, Bulgaria; 2 Department of Medical Physics and Biophysics, Faculty of Pharmacy, Medical University of Plovdiv, 4002 Plovdiv, Bulgaria; 3 Laboratory of Experimental Chemotherapy, Department “Pharmacology, Pharmacotherapy and Toxicology”, Faculty of Pharmacy, Medical University, 1431 Sofia, Bulgaria; 4 Department of Microbiology, Technological Faculty, University of Food Technologies, 4002 Plovdiv, Bulgaria; 5 Department of Pharmacognosy, Faculty of Pharmacy, Medical University of Sofia, 1000 Sofia, Bulgaria; 6 Department of Chemistry, Faculty of Pharmacy, Medical University of Sofia, 2 Dunav Str., 1000 Sofia, Bulgaria; 7 Department of Organic Chemistry, Faculty of Chemistry, University of Plovdiv, 4000 Plovdiv, Bulgaria

## Error in Figure

In the original publication [1], there was a mistake in “**Figure 5**. Histological and immunohistochemical staining of SM preparations after 1 h incubation period. (**A**,**D**,**G**,**J**,**M**,**P**) were incubated with **3**, **4a**–**d** and mebeverine hydrochloride (MH), H-E staining, ×20; (**B**,**E**,**H**,**K**,**N**,**Q**) were incubated with 5-HT, 5-HT3 expression in myenteric plexus observed (black arrows), ×20; (**C**) was incubated with **3**, increased intensity in 5-HT3 expression in myenteric plexus observed (black arrows), ×20; (**F**) was incubated with **4c**, no 5-HT3 expression observed, ×20; (**I**) incubated with **4b**, no 5-HT3 expression observed, ×20; (**L**) incubated with **4a**, no 5-HT3 expression observed, ×20; (**O**) incubated with **4d**, weak 5-HT3 expression observed (black arrows), ×20; (**R**) was incubated with MH, weak 5-HT3 expression observed (black arrows), ×20.” as published. “Some of the image files were inadvertently mislabeled or misplaced during the reassembly of the figure, which caused the observed overlap between panels A, E, J, and K.”

## The Corrected Figure 5

Figure 5 illustrates the histological and immunohistochemical findings obtained from smooth muscle (SM) preparations following a 1 h incubation period with the tested compounds. Hematoxylin and eosin (H&E) staining (A,D,G,J,M,P) was used to evaluate the general tissue morphology after incubation with compounds **3**, **4a**–**d**, and mebeverine hydrochloride (MH). The sections showed preserved structural integrity of the smooth muscle tissue without visible signs of degeneration or necrosis, confirming that the experimental conditions and tested substances did not adversely affect tissue morphology. Images were taken at magnifications ×5 and ×20 (Supplementary Materials).

Immunohistochemical staining was performed to assess the expression and localization of the 5-HT3 receptor within the myenteric plexus. In the samples incubated with 5-HT (B,E,H,K,N,Q), a strong immunopositive reaction was detected, with pronounced 5-HT3 receptor expression clearly visible in the myenteric plexus (indicated by black stars), confirming the sensitivity and reliability of the staining procedure.

In samples treated with compound **3** (C), a marked increase in 5-HT3 receptor expression was observed, comparable to that seen in the 5-HT-treated control, suggesting a potential stimulatory effect of compound **3** on receptor expression. Samples incubated with compounds **4a**, **4b**, and **4c** exhibited either absent or very weak 5-HT3 immunoreactivity, as reflected by the pale staining intensity in the myenteric plexus, indicating minimal or undetectable receptor expression under these conditions. Similarly, treatment with compound **4d** (O) resulted in weak immunopositive staining within the myenteric plexus, suggesting a limited stimulatory effect on 5-HT3 receptor expression. Incubation with mebeverine hydrochloride (MH) (R) also led to weak 5-HT3 expression, comparable to that of compound **4d**.

Overall, the histological evaluation confirmed tissue preservation across all experimental conditions, while the immunohistochemical analysis revealed distinct patterns of 5-HT3 receptor expression depending on the compound used, with compound **3** demonstrating the most pronounced stimulatory effect.

The authors state that the scientific conclusions are unaffected. This correction was approved by the Academic Editor. The original publication has also been updated.

## Figures and Tables

**Figure 5:**
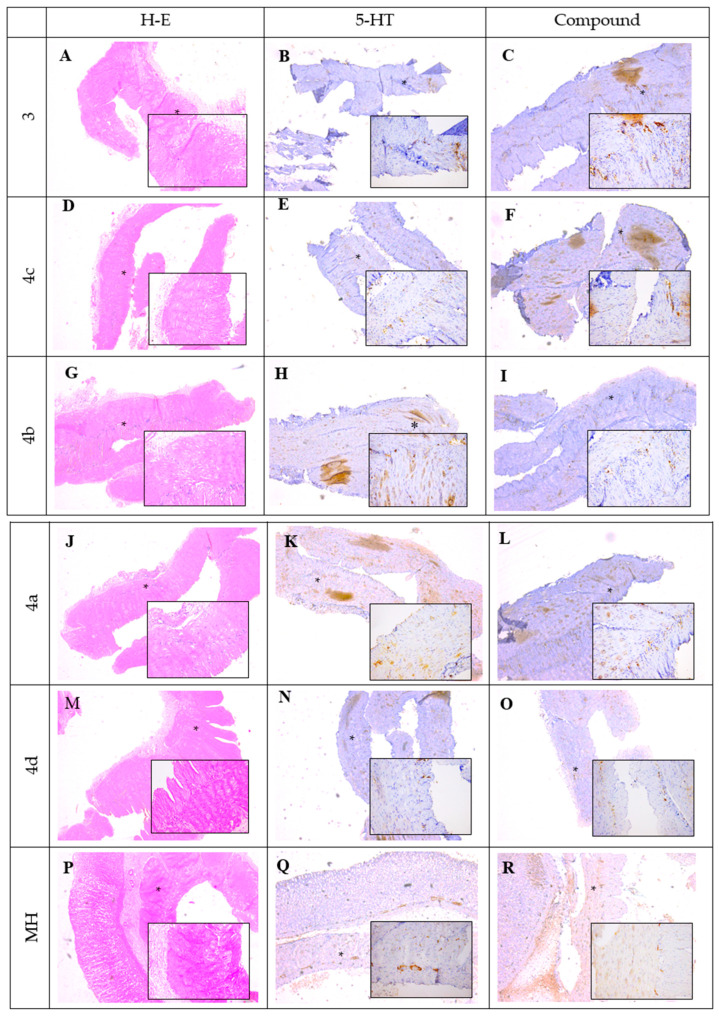
Histological and immunohistochemical staining of SM preparations after 1 h incubation period. (**A**,**D**,**G**,**J**,**M**,**P**) were incubated with **3**, **4a**–**d** and mebeverine hydrochloride (MH), H-E staining, ×5, ×20; (**B**,**E**,**H**,**K**,**N**,**Q**) were incubated with 5-HT, 5-HT3 expression observed in the myenteric plexus (black stars), ×5, ×20; (**C**) was incubated with **3**, with increased intensity in 5-HT3 expression in myenteric plexus observed (black stars), ×5, ×20; (**F**) was incubated with **4c**, with no 5-HT3 expression observed, ×5, ×20; (**I**) was incubated with **4b**, with no 5-HT3 expression observed, ×5, ×20; (**L**) incubated was incubated with **4a**, with no 5-HT3 expression observed, ×5, ×20; (**O**) was incubated with **4d**, with weak 5-HT3 expression observed (black stars), ×5, ×20; and (**R**) was incubated with MH, with weak 5-HT3 expression observed (black stars), ×5, ×20.

## Supplementary Materials

The following supporting information can be downloaded at: https://www.mdpi.com/article/10.3390/biomedicines13102564/s1.
