# Supplementary material for: In Silico, In Vitro, and Ex Vivo Biological Activity of Some Novel Mebeverine Precursors
Source: Biomedicines. 2023 Feb 17;11(2):605. doi: 10.3390/biomedicines11020605 (PMC9953468; doi:10.3390/biomedicines11020605)

In silico, in vitro, and *ex vivo* biological activity of some novel mebeverine precursors

Miglena Milusheva, Vera Gledacheva, Iliyana Stefanova, Mina Pencheva, Rositsa Mihaylova, Yulian Tumbarski, Paraskev Nedialkov, Emiliya Cherneva, Mina Todorova, Stoyanka Nikolova

Table of Contents:

Figure S1: ^{1}H-NMR spectrum of compound **3**, page 2

Figure S2: ^{13}C-NMR spectrum of compound **3**, page 3

Figure S3: DEPT spectrum of compound **3**, page 4

Figure S4: IR spectrum of compound **3**, page 5

Figure S5: ^{1}H-NMR spectrum of compound **4a**, page 6

Figure S6: ^{13}C-NMR spectrum of compound **4a**, page 7

Figure S7: DEPT spectrum of compound **4a**, page 8

Figure S8: IR spectrum of compound **4a**, page 9

Figure S9: ^{1}H-NMR spectrum of compound **4b**, page 10

Figure S10: ^{13}C-NMR spectrum of compound **4b**, page 11

Figure S11: DEPT spectrum of compound **4b**, page 12

Figure S12: IR spectrum of compound **4b**, page 13

Figure S13: ^{1}H-NMR spectrum of compound **4c**, page 14

Figure S14: ^{13}C-NMR spectrum of compound **4c**, page 15

Figure S15: DEPT spectrum of compound **4c**, page 16

Figure S16: IR spectrum of compound **4c**, page 17

Figure S17: ^{1}H-NMR spectrum of compound **4d**, page 18

Figure S18: ^{13}C-NMR spectrum of compound **4d**, page 19

Figure S19: DEPT spectrum of compound **4d**, page 20

Figure S20: IR spectrum of compound **4d**, page 21

Figure S1: ^{1}H-NMR spectrum of compound **3**

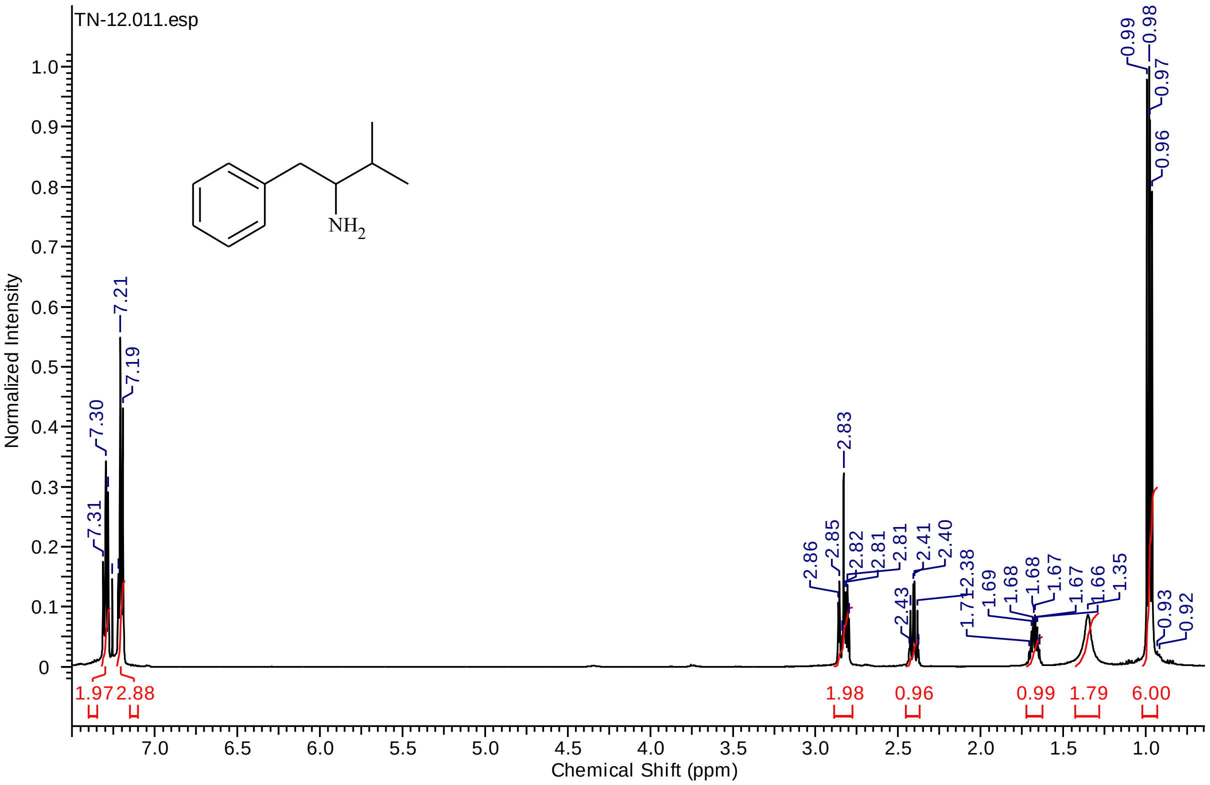

Figure S2: ^{13}C-NMR spectrum of compound **3**

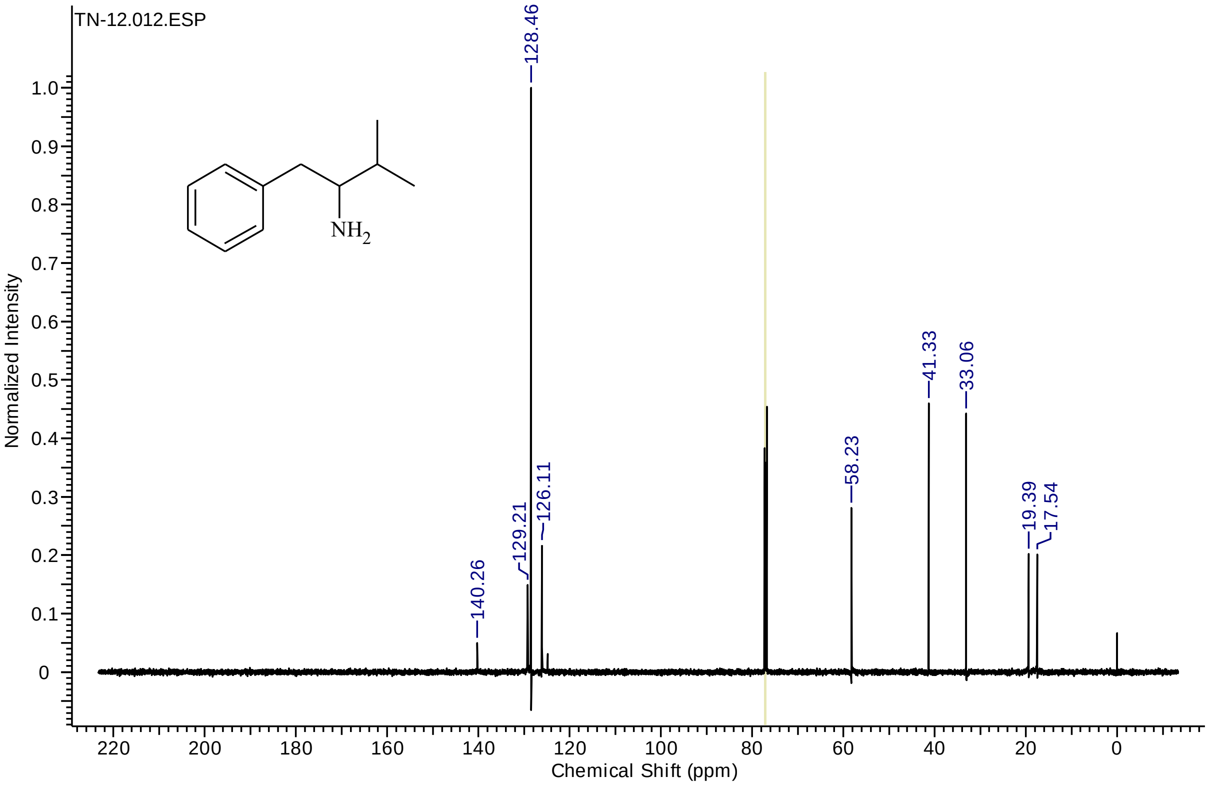

Figure S3: DEPT spectrum of compound **3**

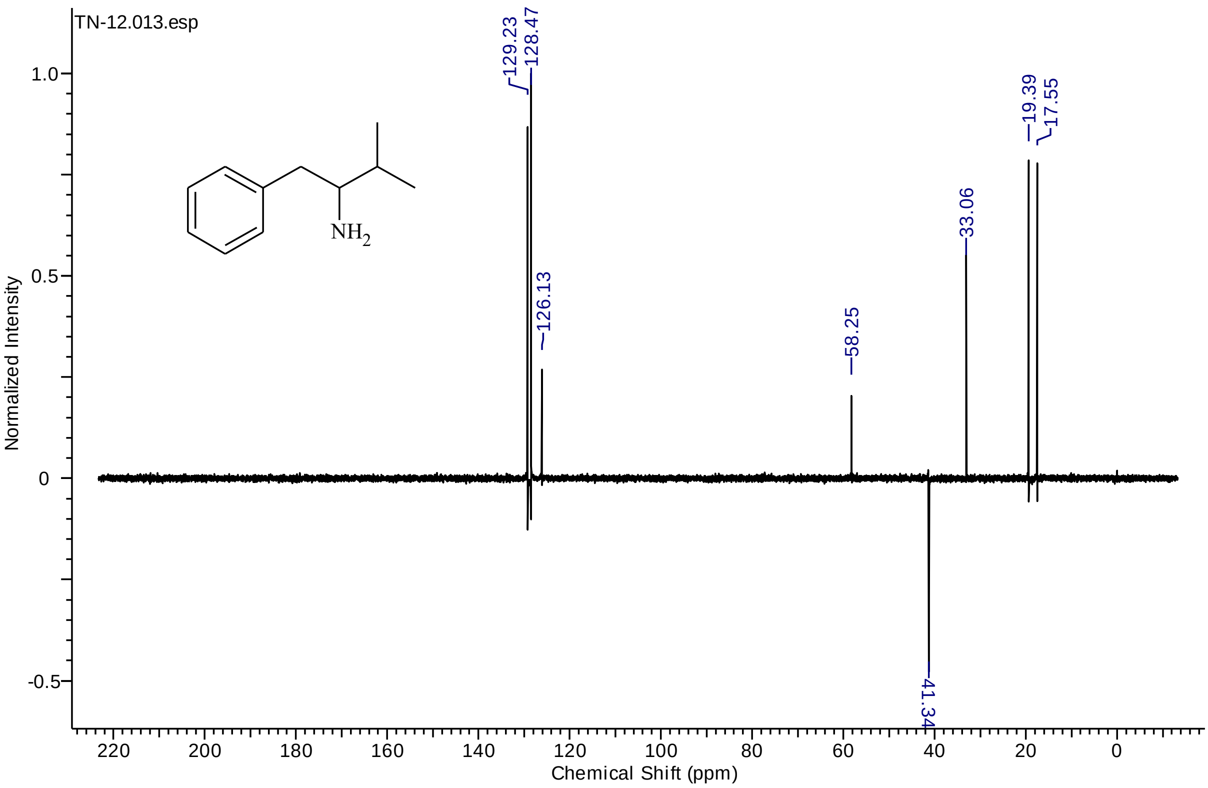

Figure S4: IR spectrum of compound **3**

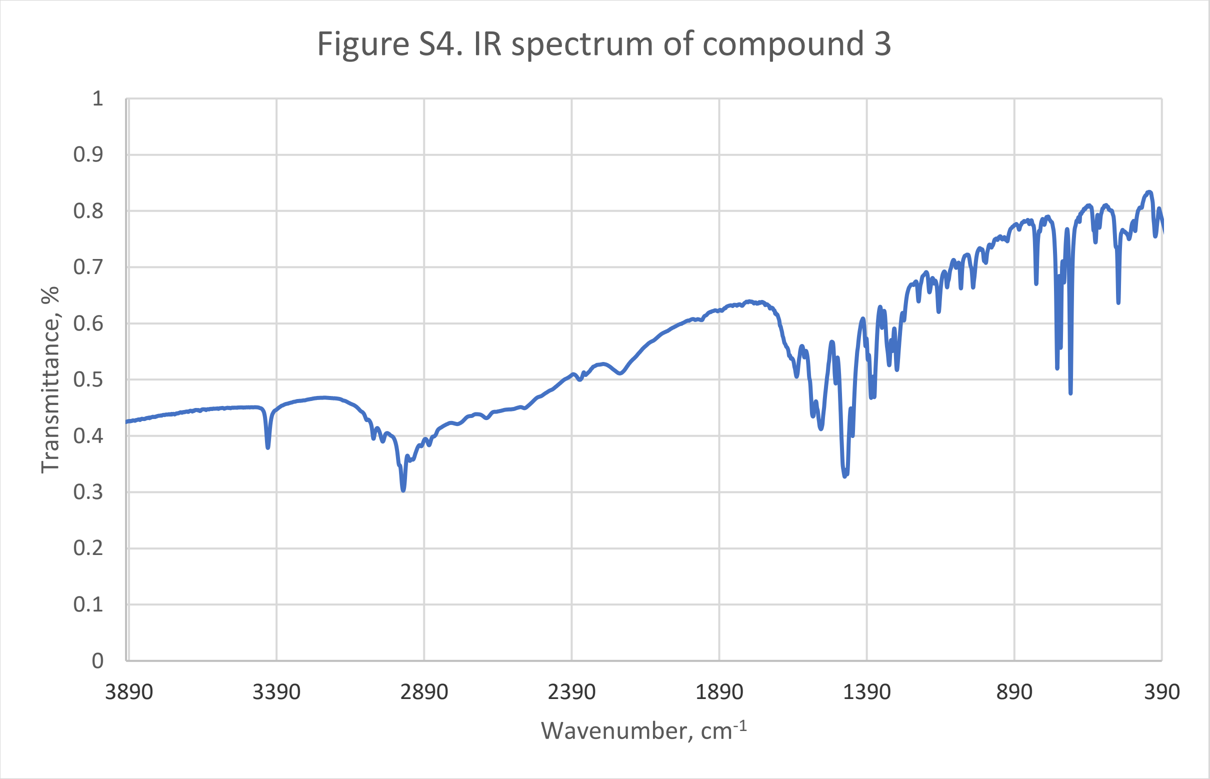

Figure S5: ^{1}H-NMR spectrum of compound **4a**

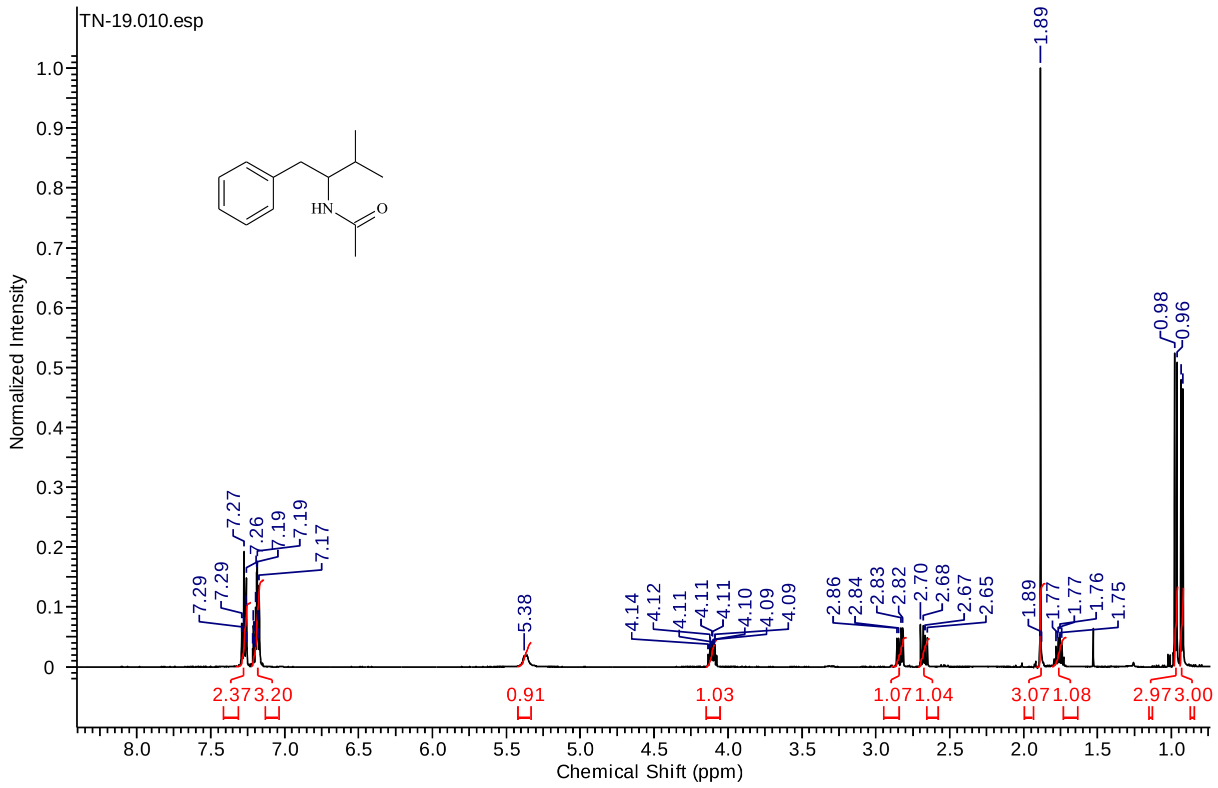

Figure S6: ^{13}C-NMR spectrum of compound **4a**

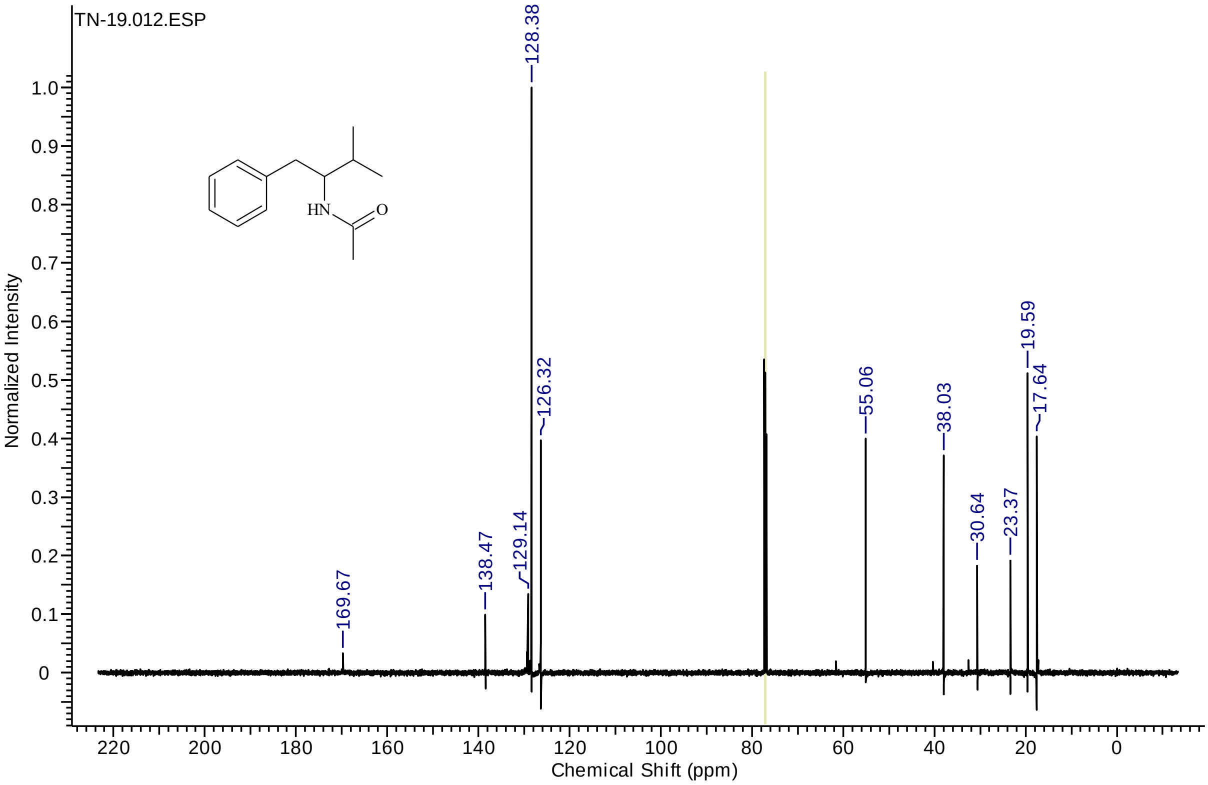

Figure S7: DEPT spectrum of compound **4a**

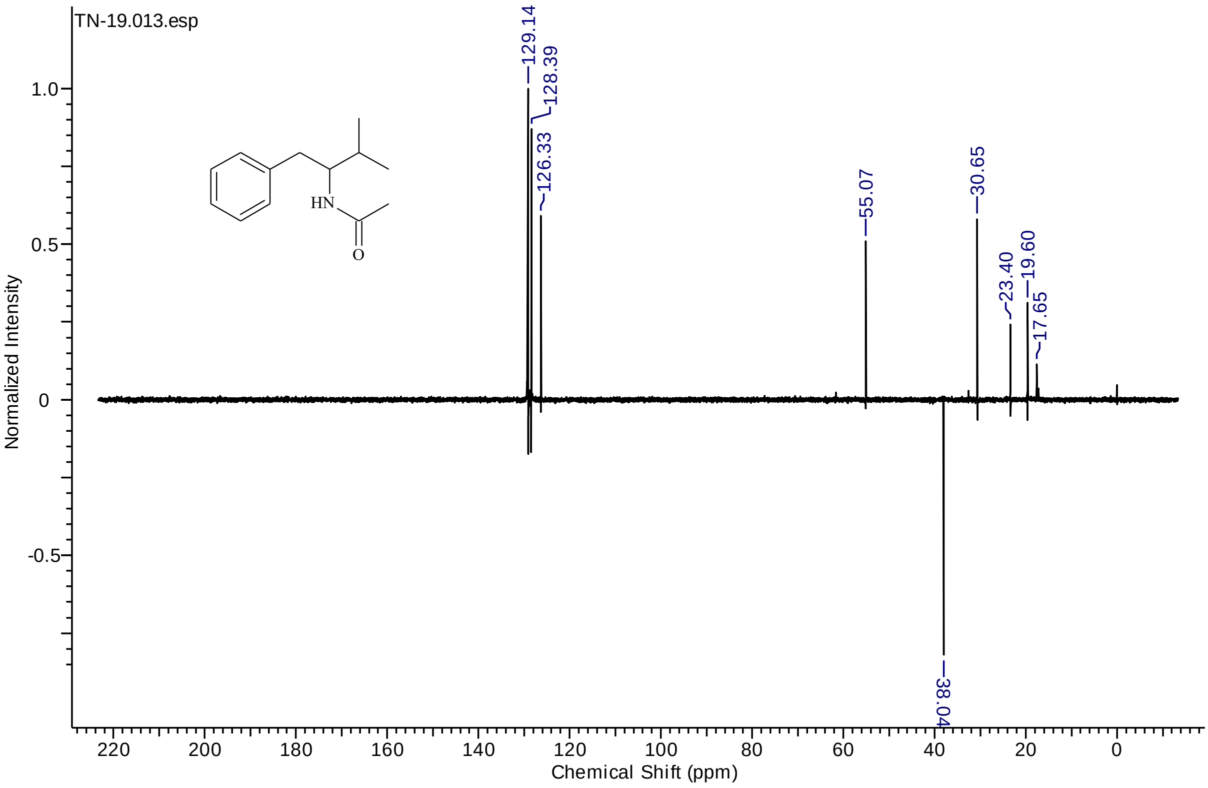

Figure S8: IR spectrum of compound **4a**

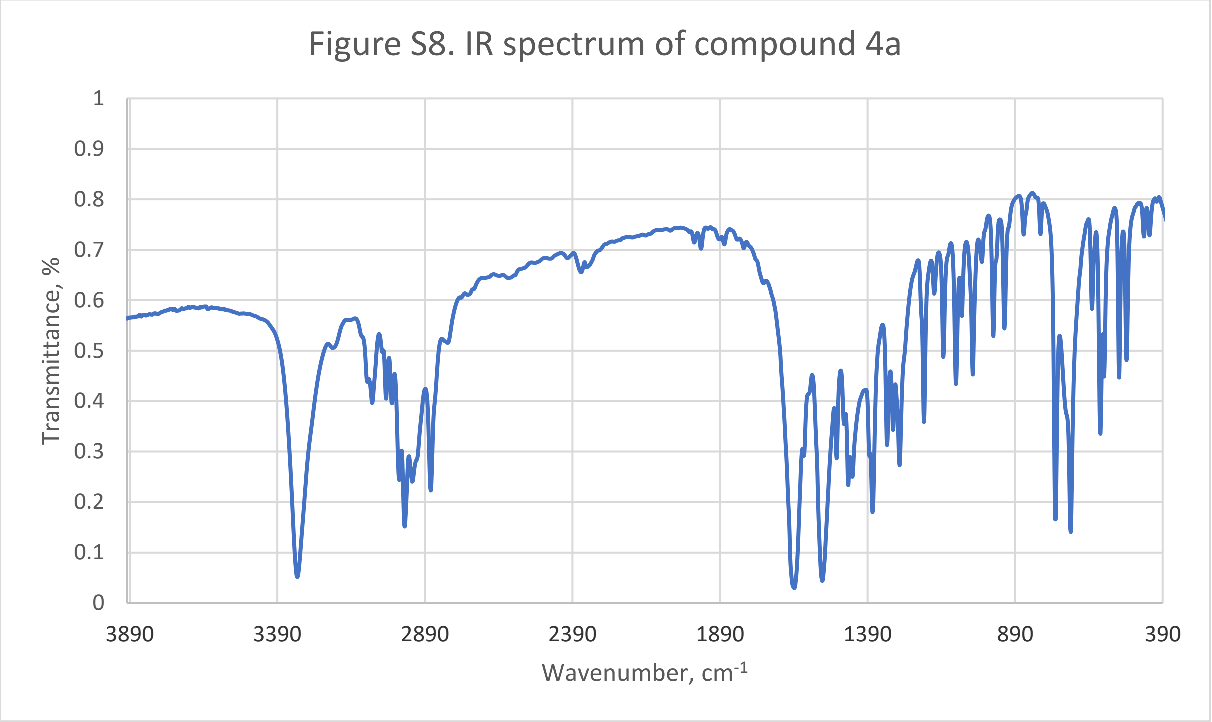

Figure S9: ^{1}H-NMR spectrum of compound **4b**

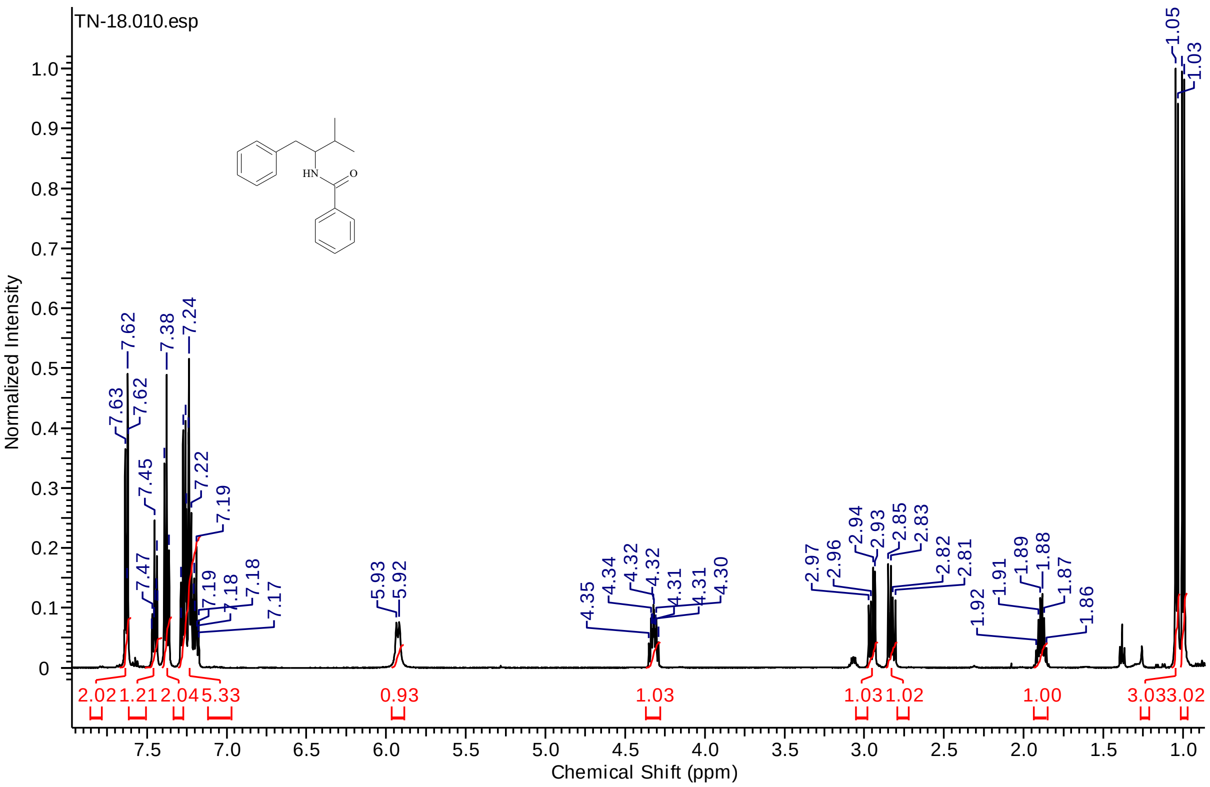

Figure S10: ^{13}C-NMR spectrum of compound **4b**

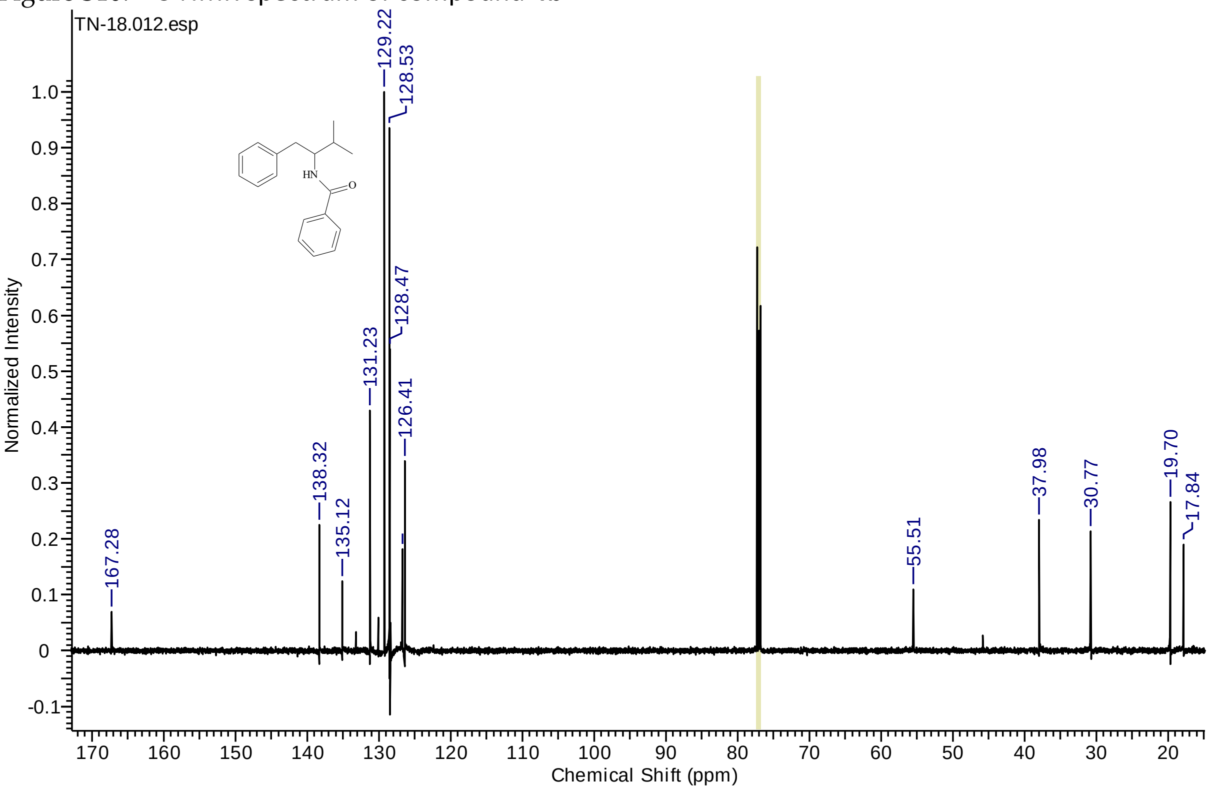

Figure S11: DEPT spectrum of the compound **4b**

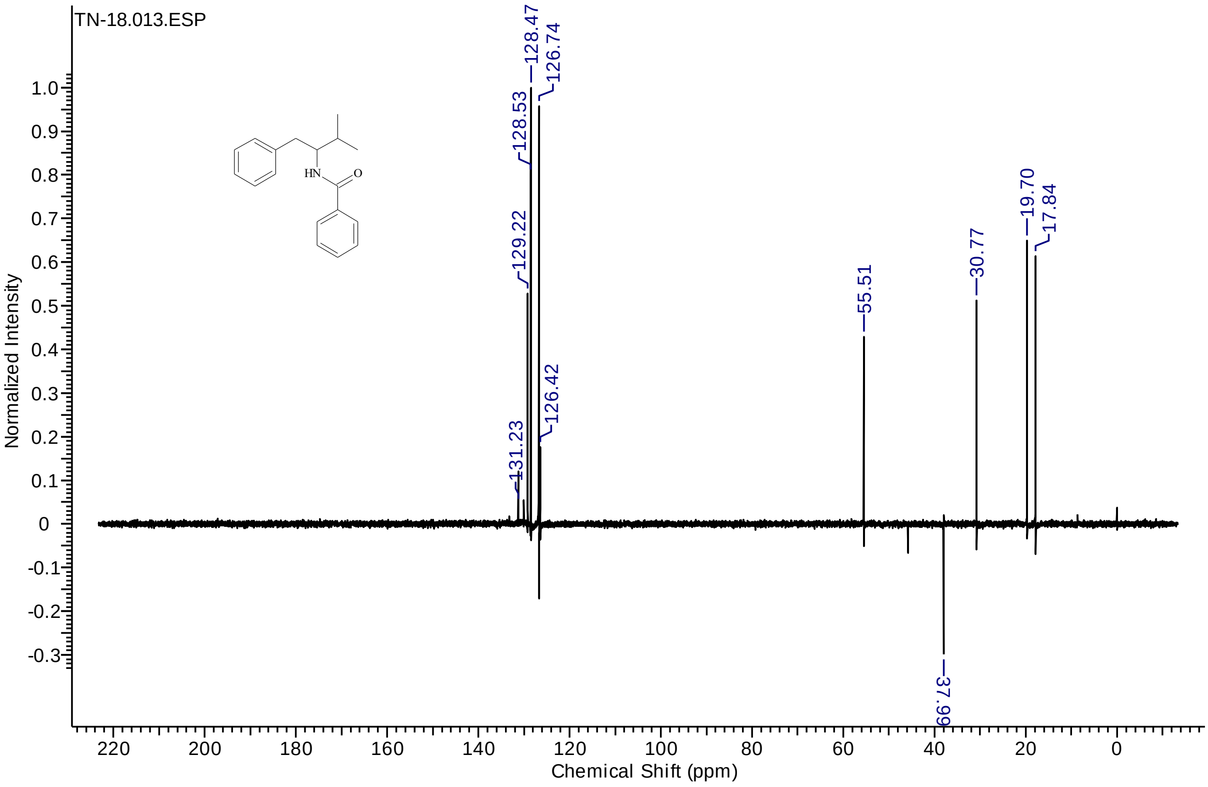

Figure S12: IR spectrum of compound **4b**

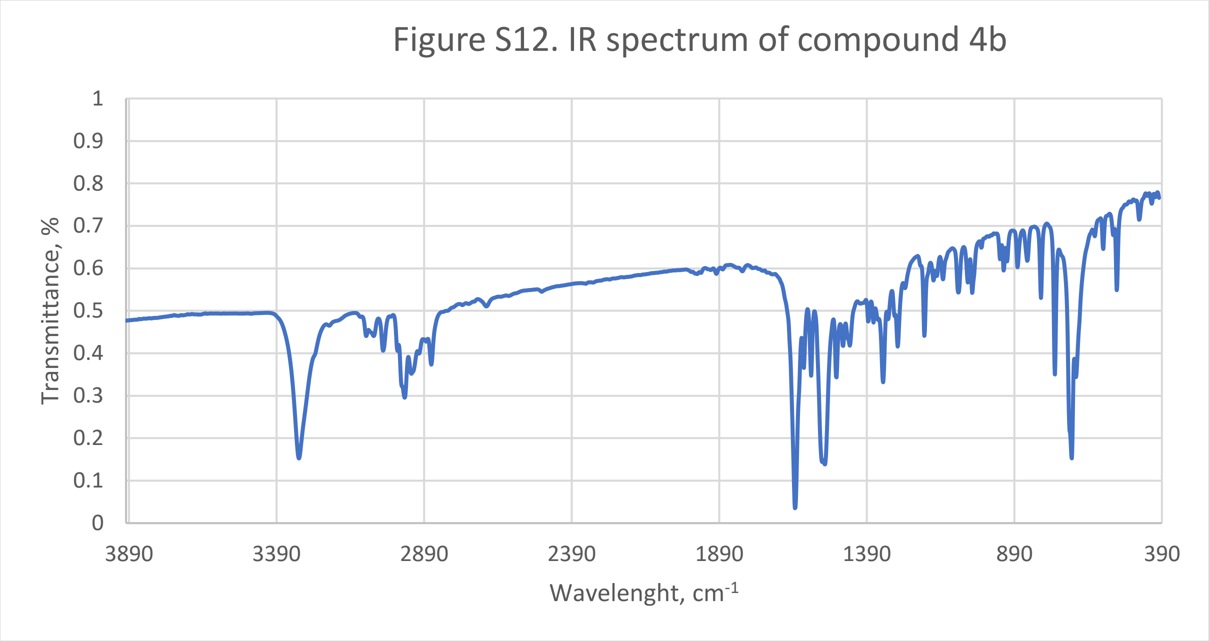

Figure S13: ^{1}H-NMR spectrum of compound **4c**

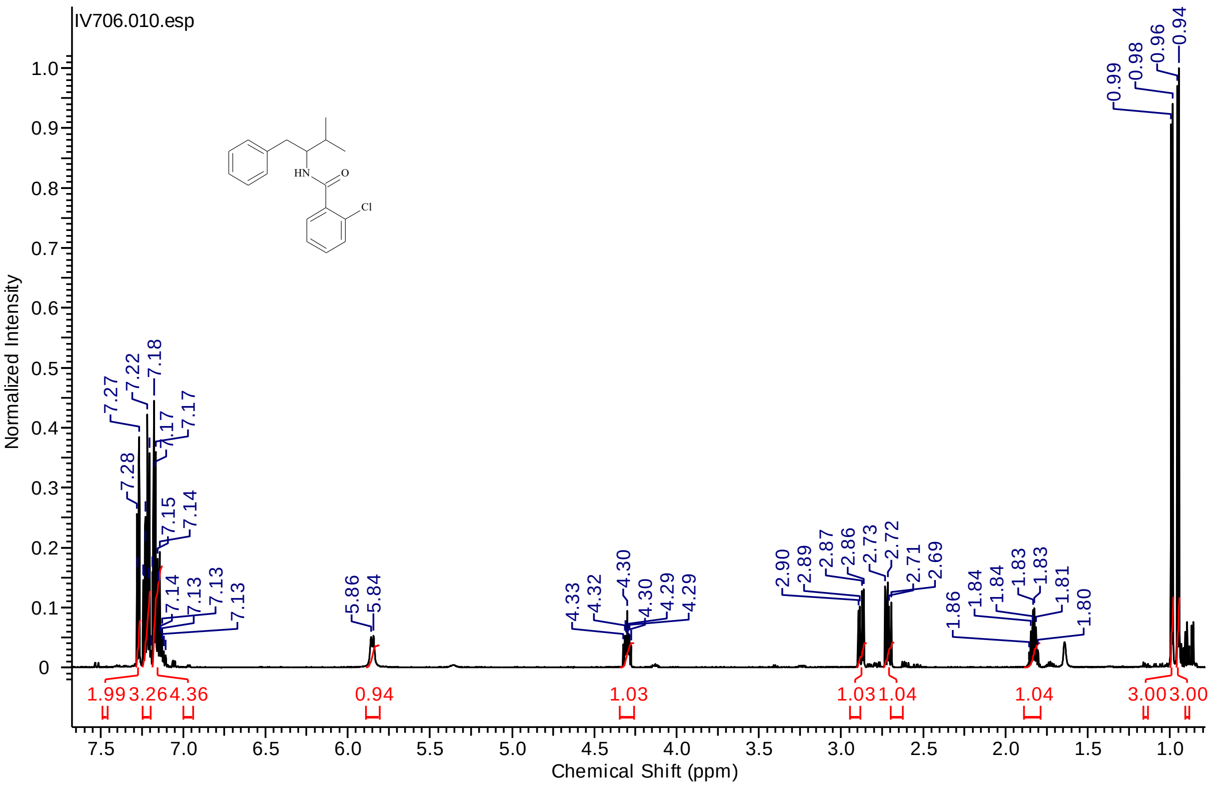

Figure S14: ^{13}C-NMR spectrum of compound **4c**

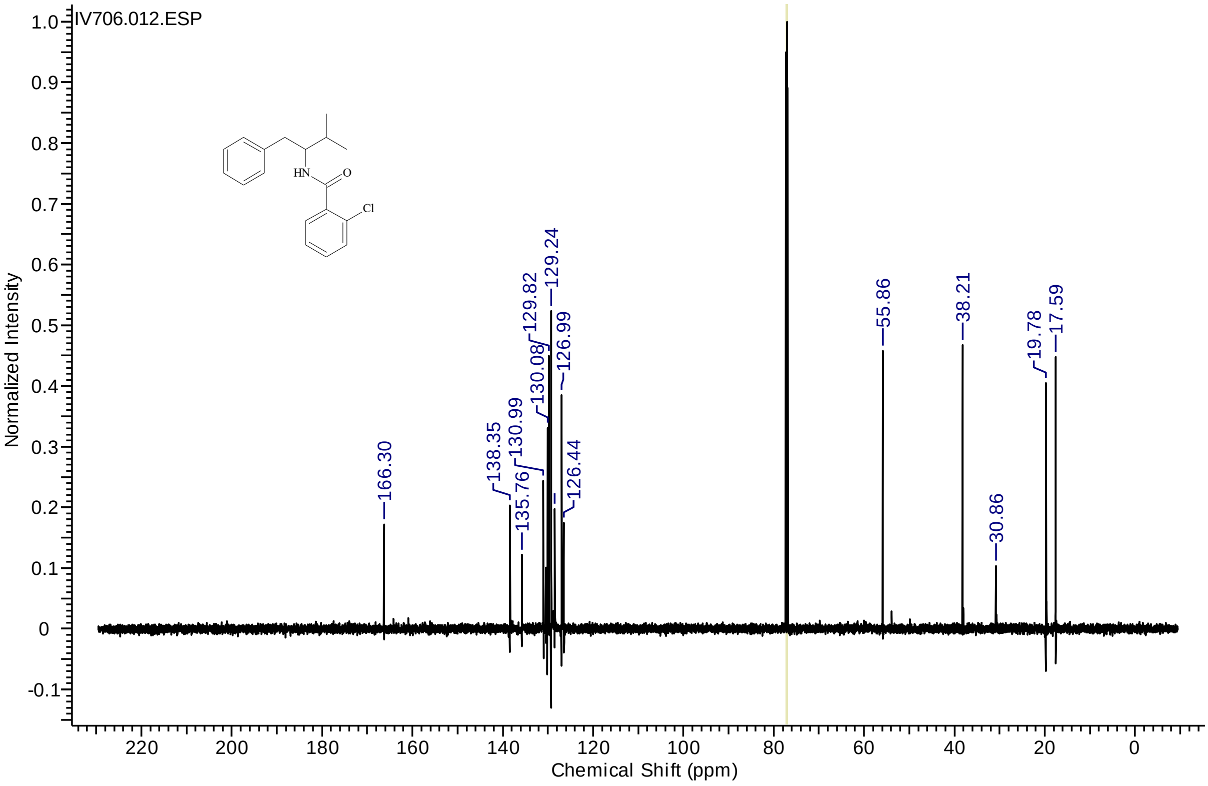

Figure S15: DEPT spectrum of the compound **4c**

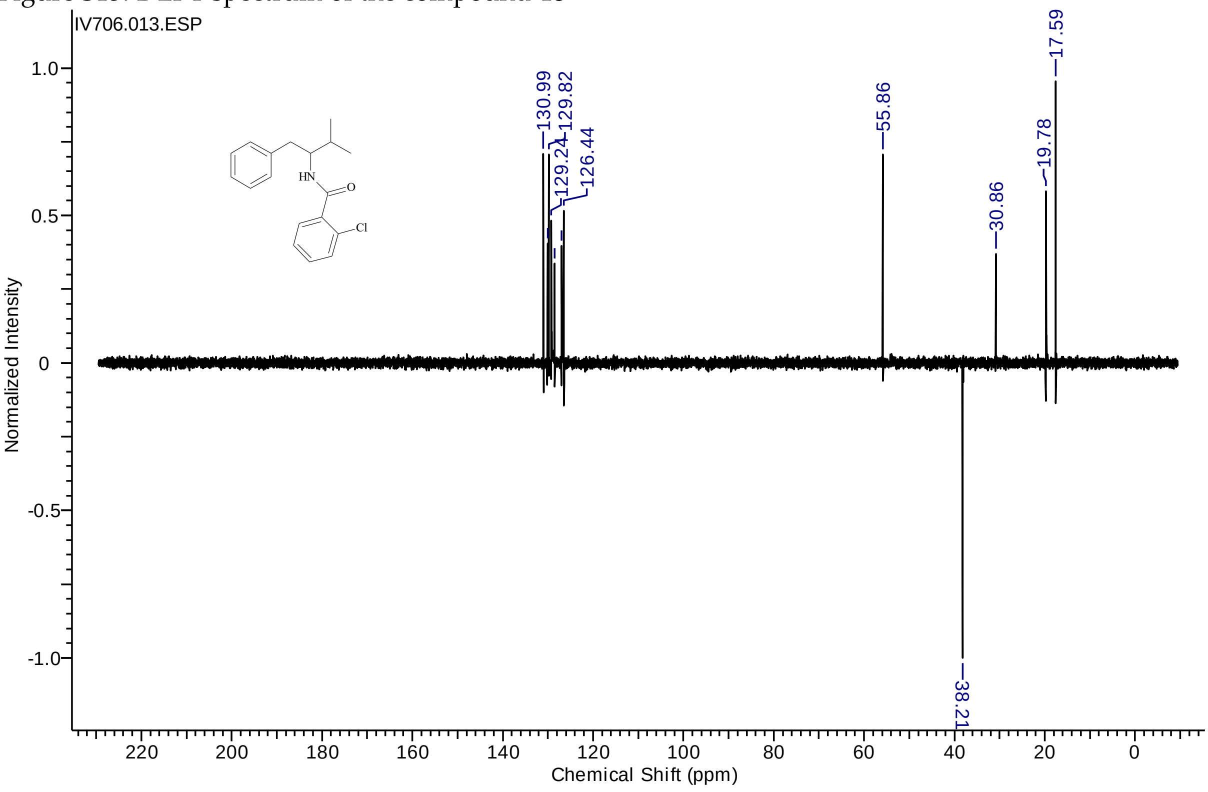

Figure S16: IR spectrum of compound **4c**

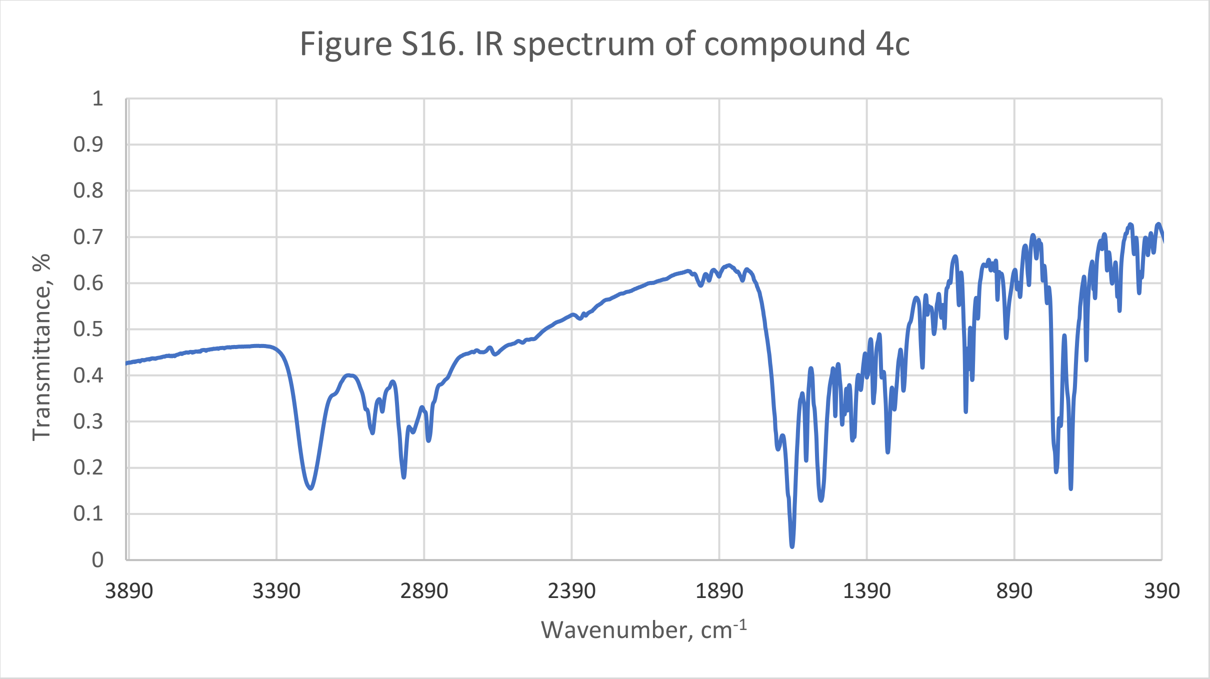

Figure S17: ^{1}H-NMR spectrum of compound **4d**

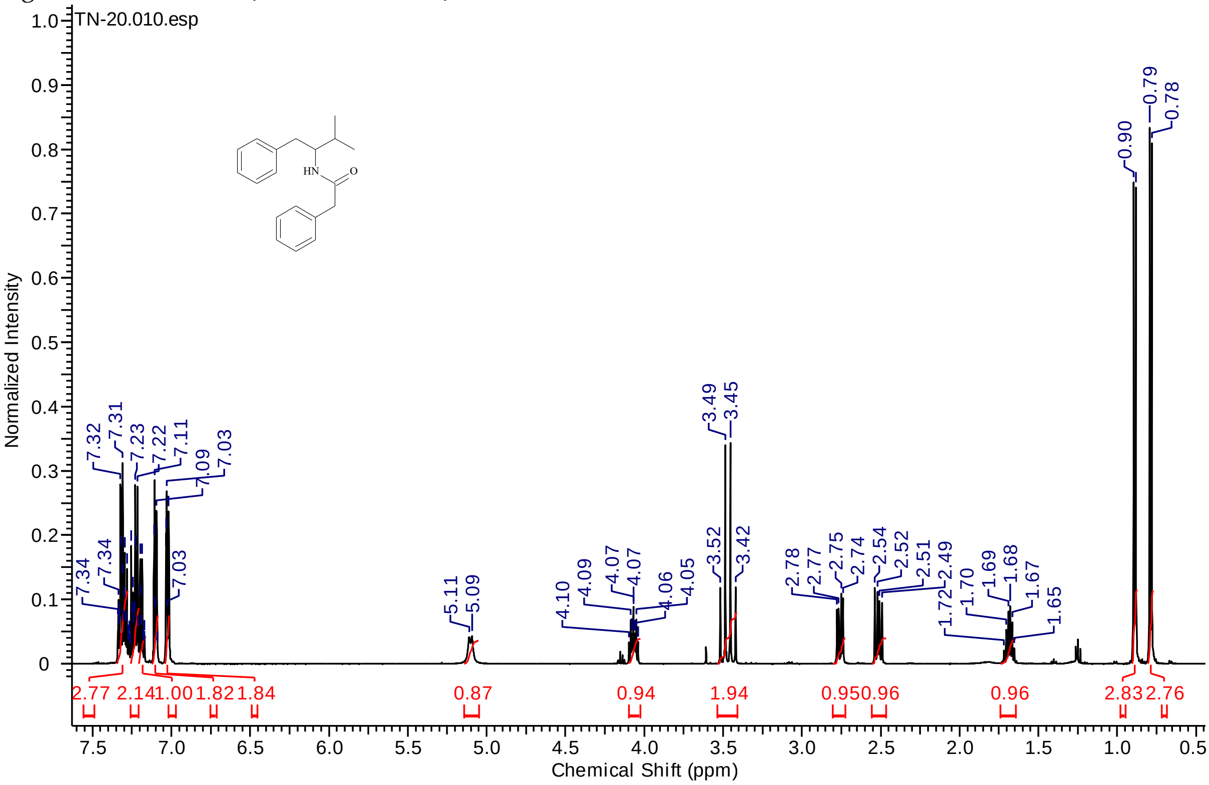

Figure S18: ^{13}C-NMR spectrum of compound **4d**

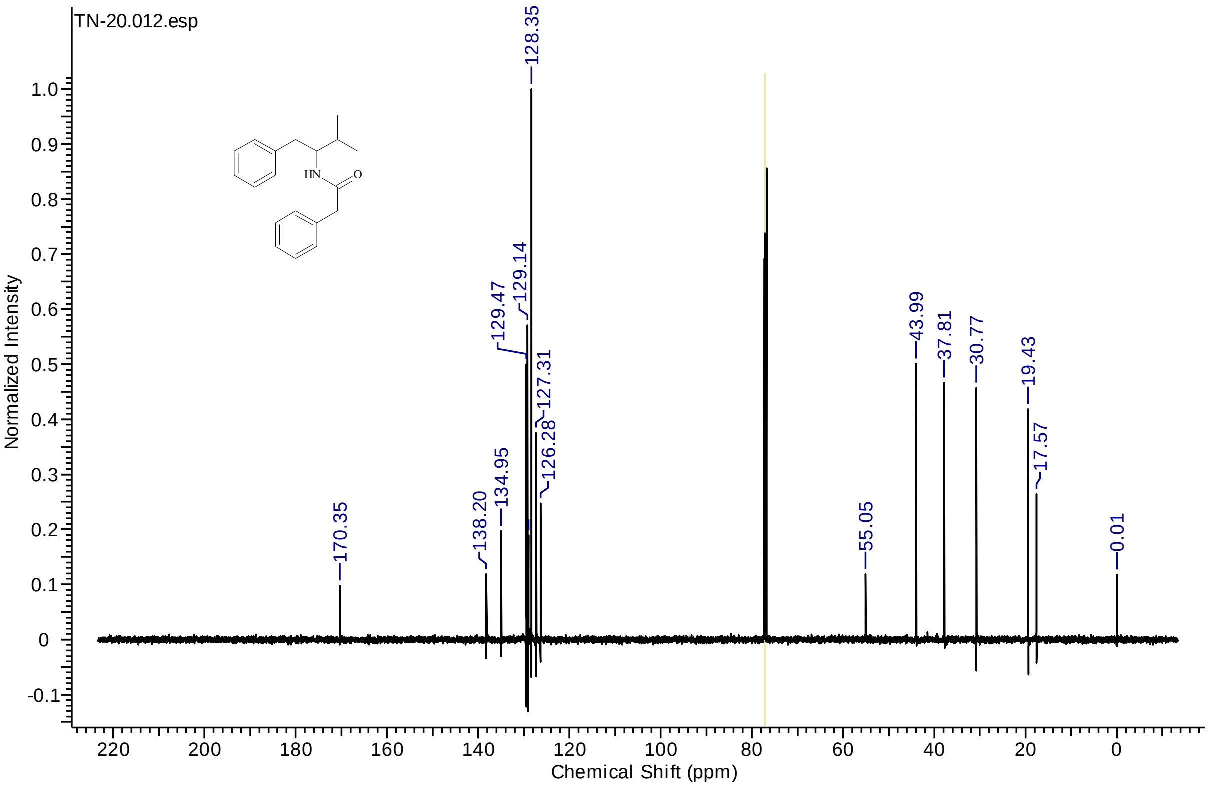

Figure S19: DEPT spectrum of the compound **4d**

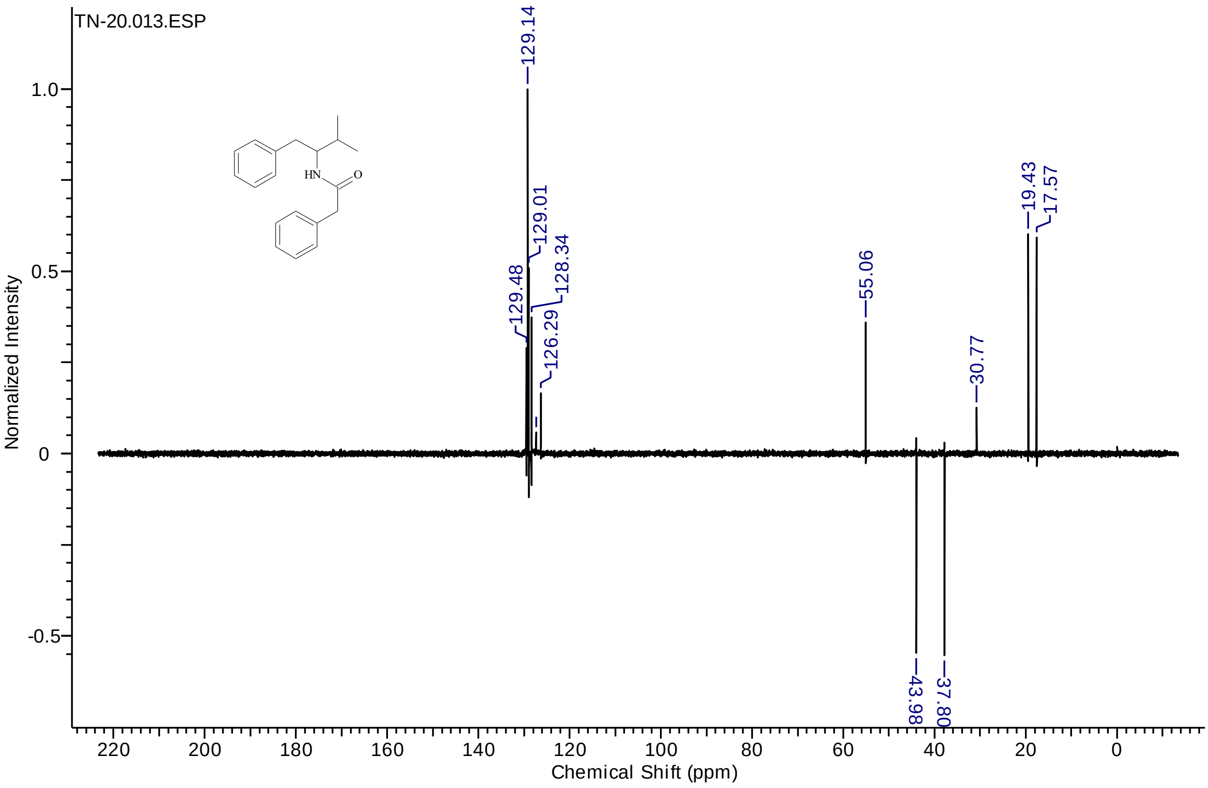

Figure S20: IR spectrum of compound **4d**

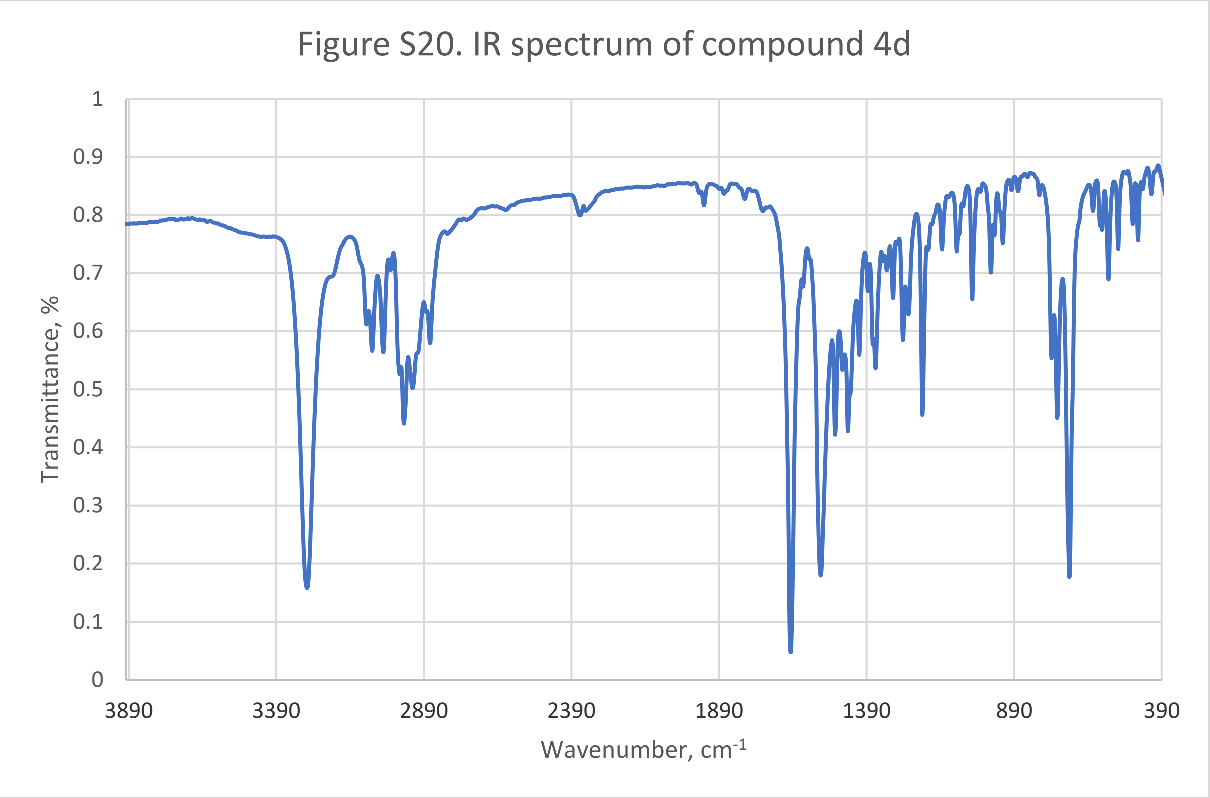

Supplement: Supplementary file 1 [file biomedicines-11-00605-s001.zip › biomedicines-2206819-supplementary.pdf]
