# Supplementary figures and images for: Correction: Milusheva et al. In Silico, In Vitro, and Ex Vivo Biological Activity of Some Novel Mebeverine Precursors. Biomedicines 2023, 11, 605
Source: Biomedicines. 2025 Oct 21;13(10):2564. doi: 10.3390/biomedicines13102564 (PMC12562134; doi:10.3390/biomedicines13102564)

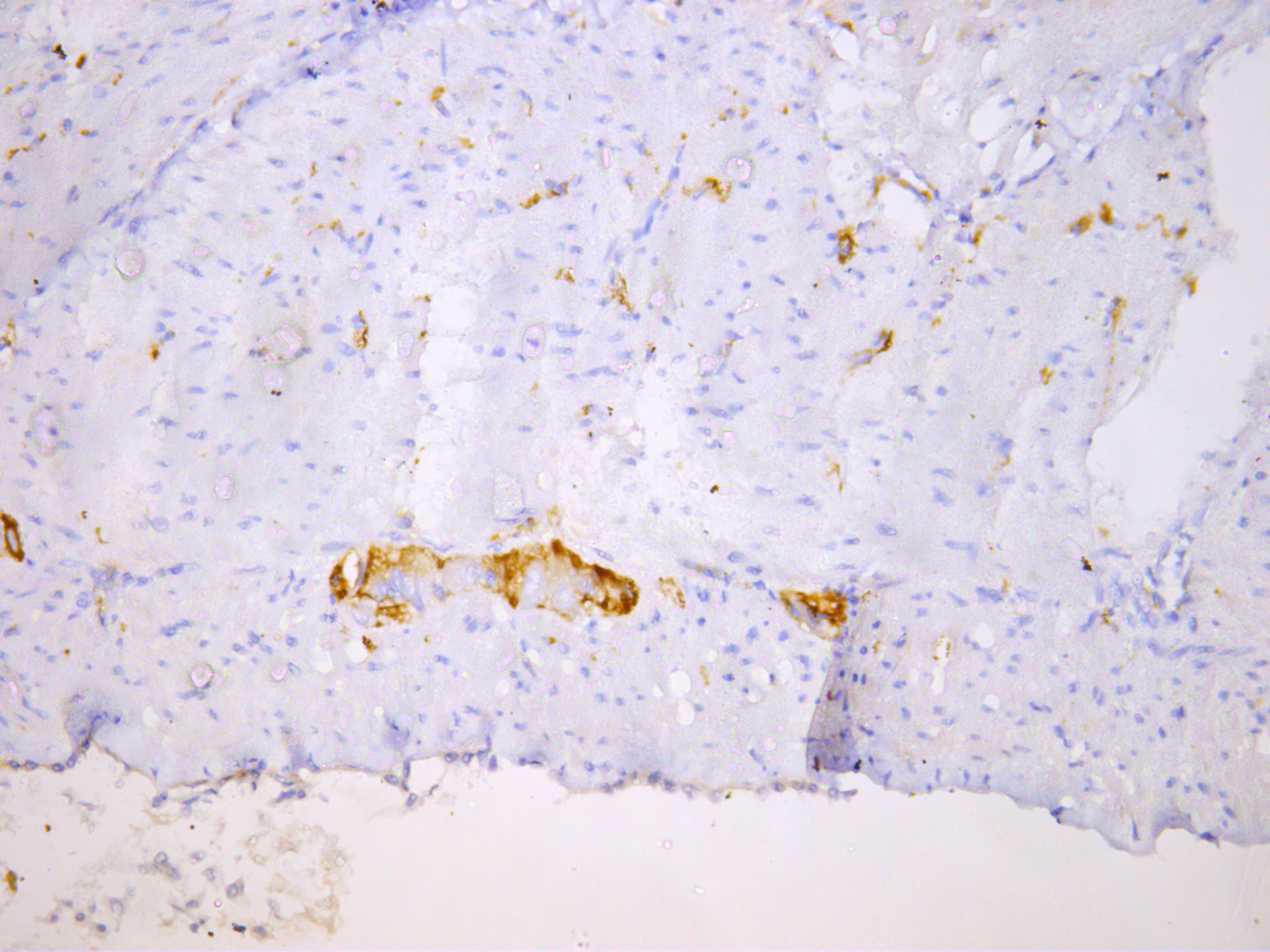

Supplement: Supplementary file 1 [file biomedicines-13-02564-s001.zip › Fig 5 Q x20 .tif]

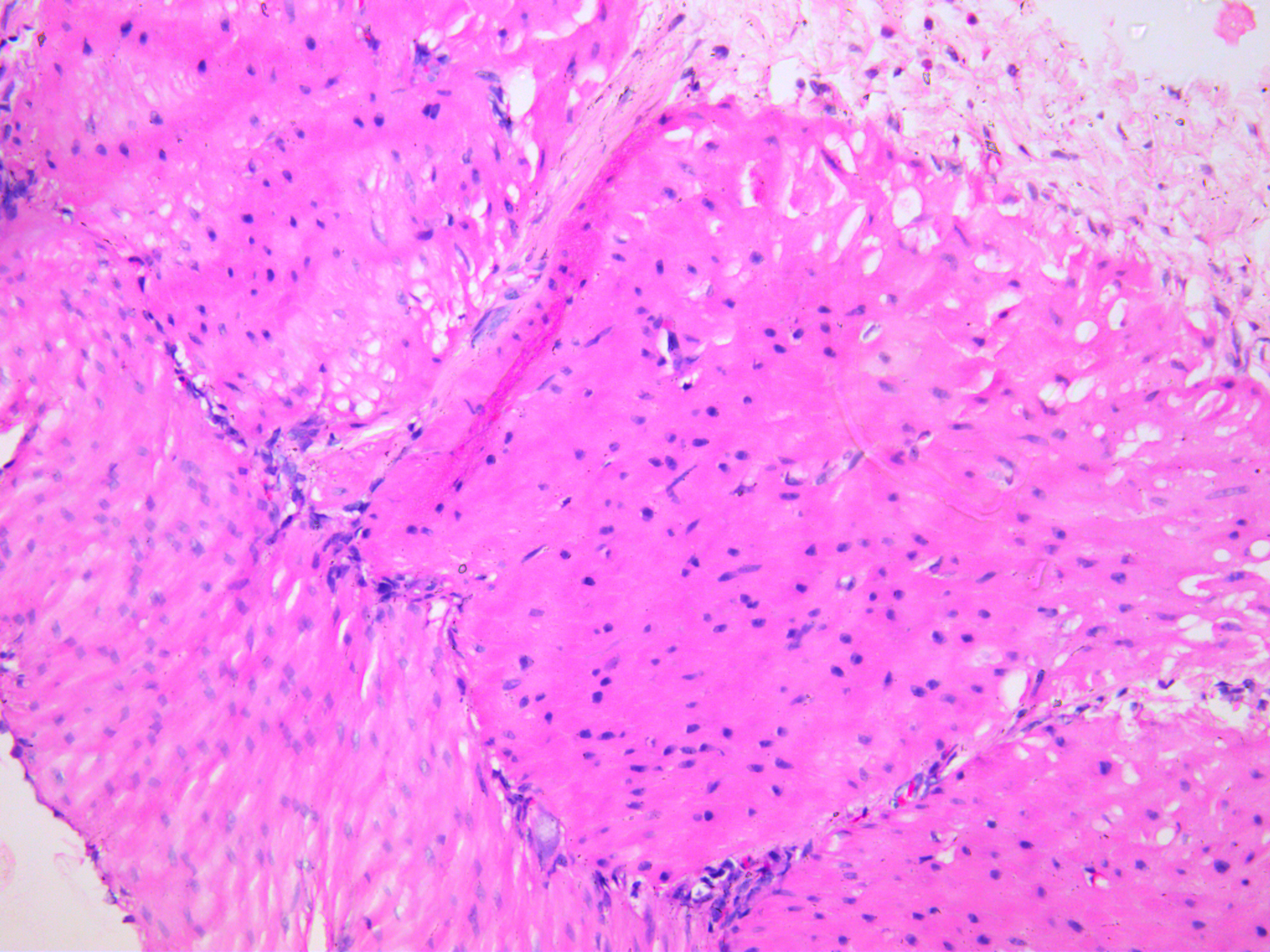

Supplement: Supplementary file 1 [file biomedicines-13-02564-s001.zip › Fig 5 A x20 .tif]

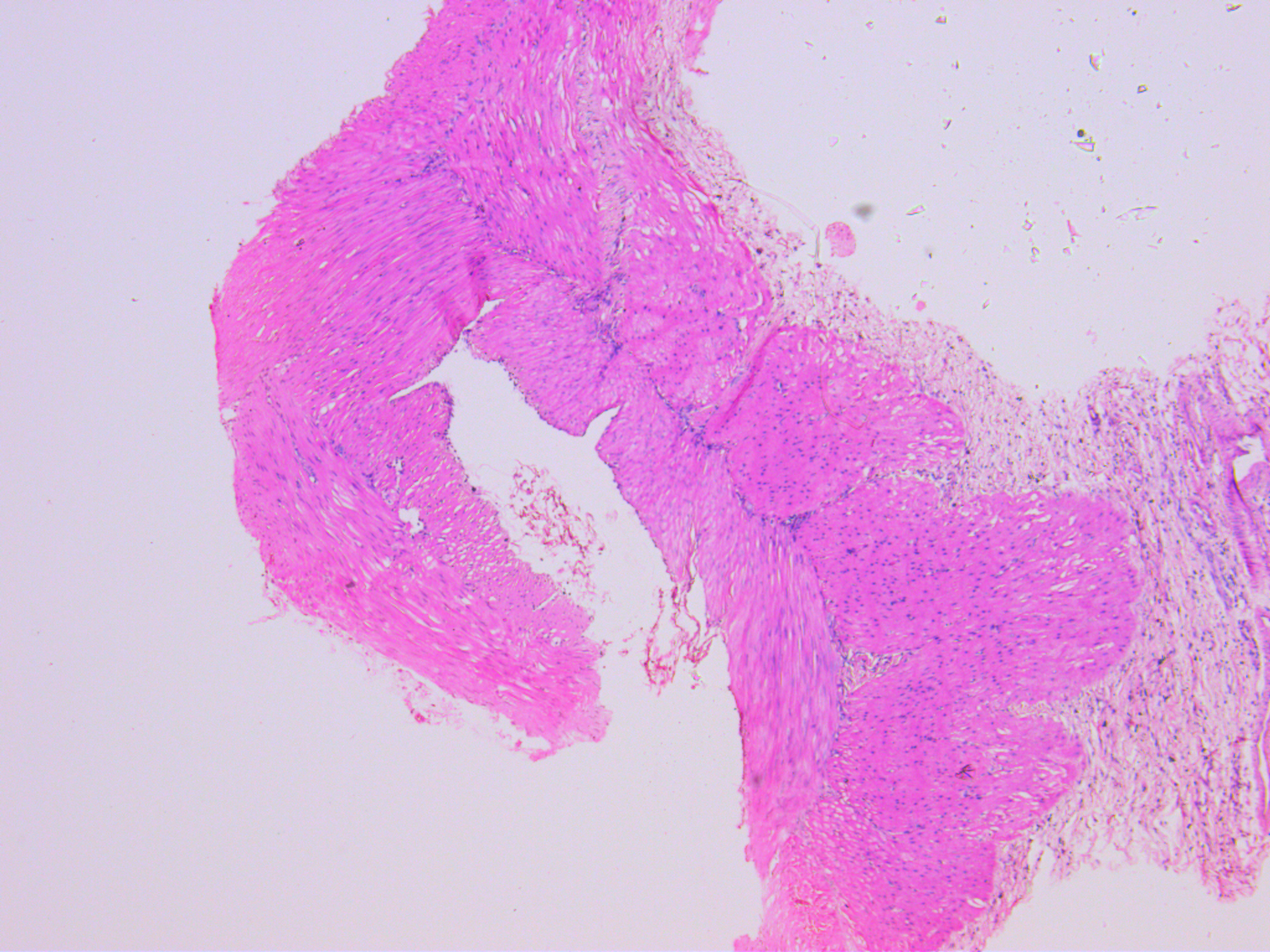

Supplement: Supplementary file 1 [file biomedicines-13-02564-s001.zip › Fig 5 A x5 .tif]

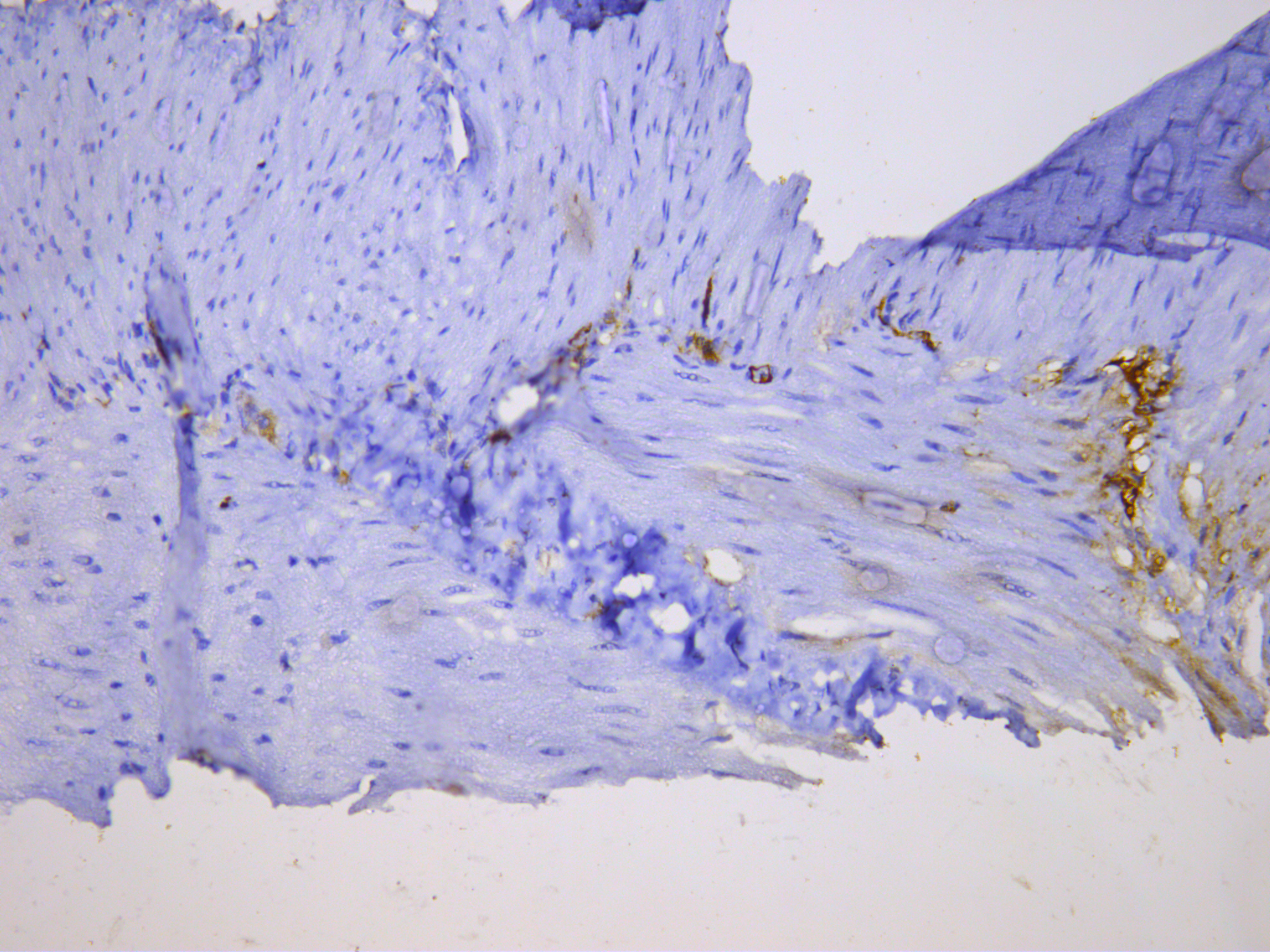

Supplement: Supplementary file 1 [file biomedicines-13-02564-s001.zip › Fig 5 B x20 .tif]

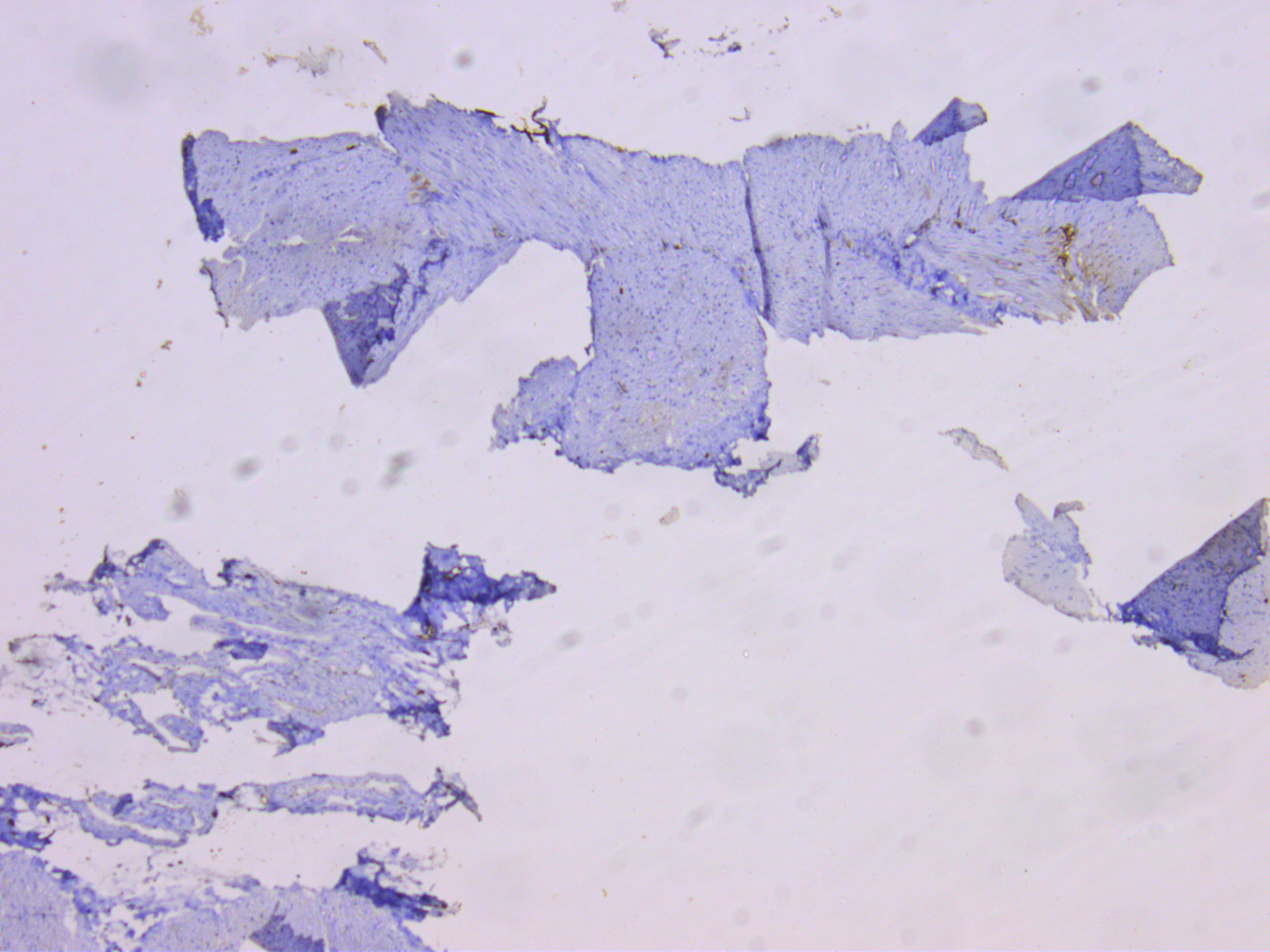

Supplement: Supplementary file 1 [file biomedicines-13-02564-s001.zip › Fig 5 B x5 .tif]

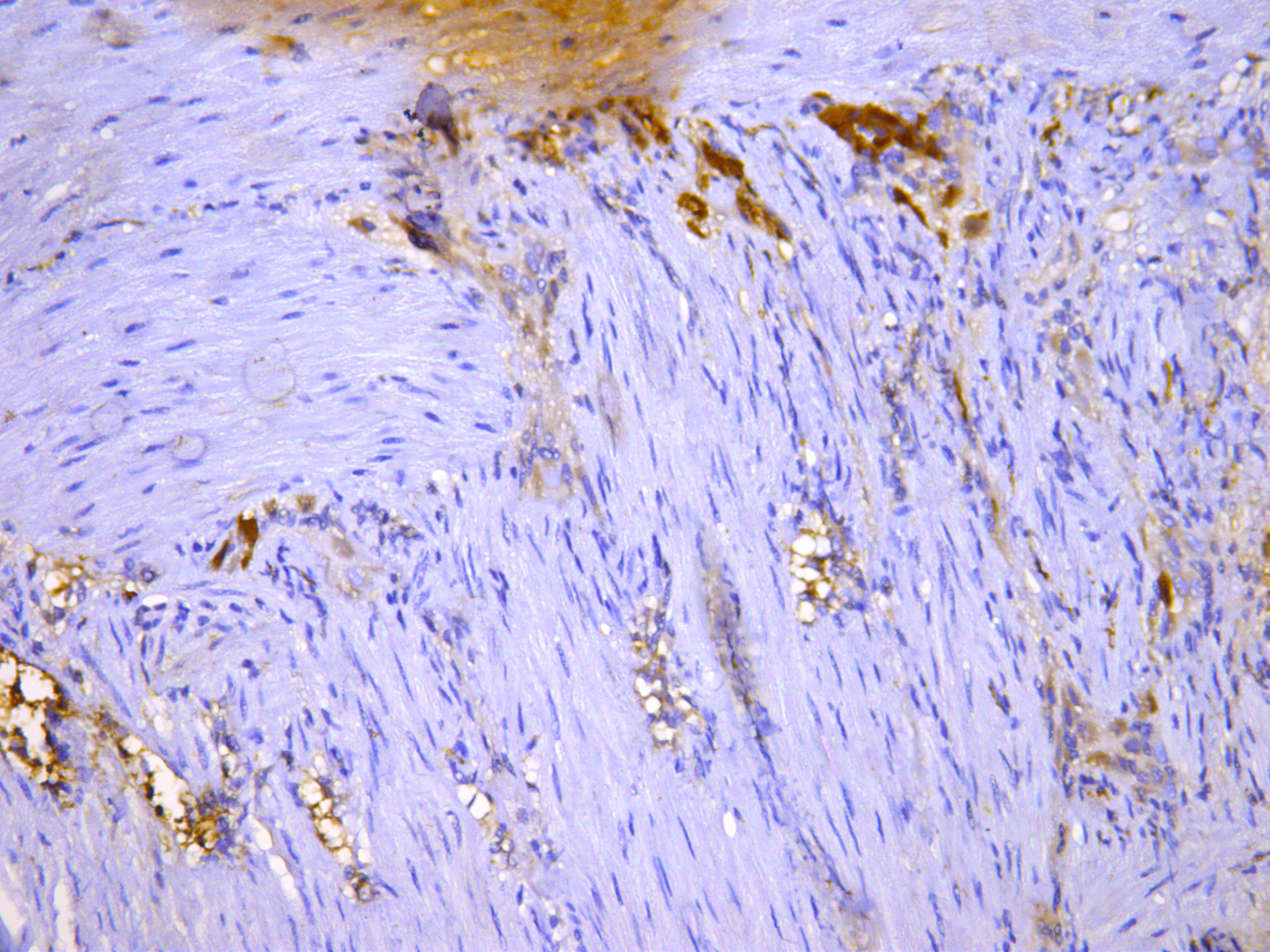

Supplement: Supplementary file 1 [file biomedicines-13-02564-s001.zip › Fig 5 C x20 .tif]

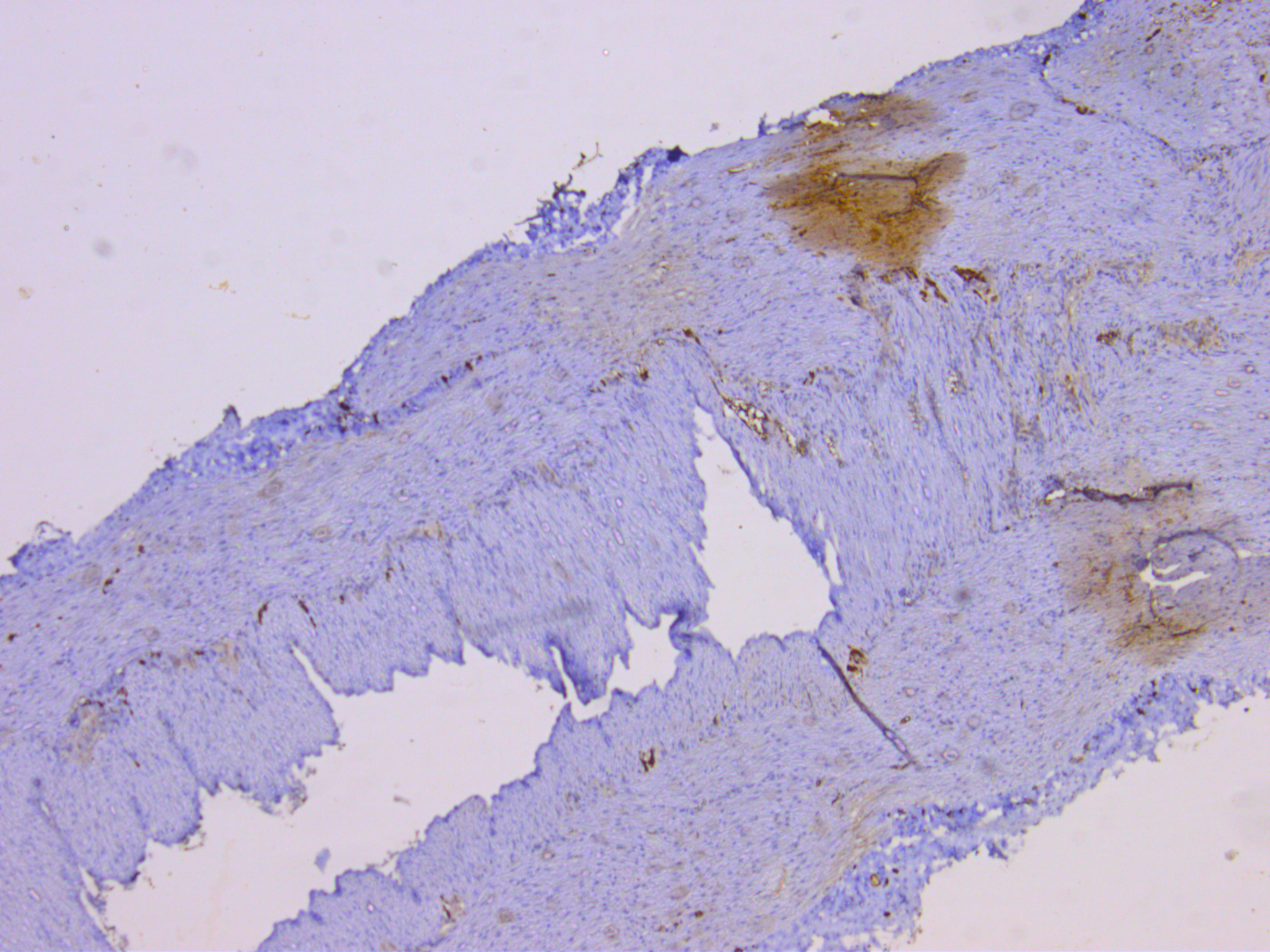

Supplement: Supplementary file 1 [file biomedicines-13-02564-s001.zip › Fig 5 C x5 .tif]

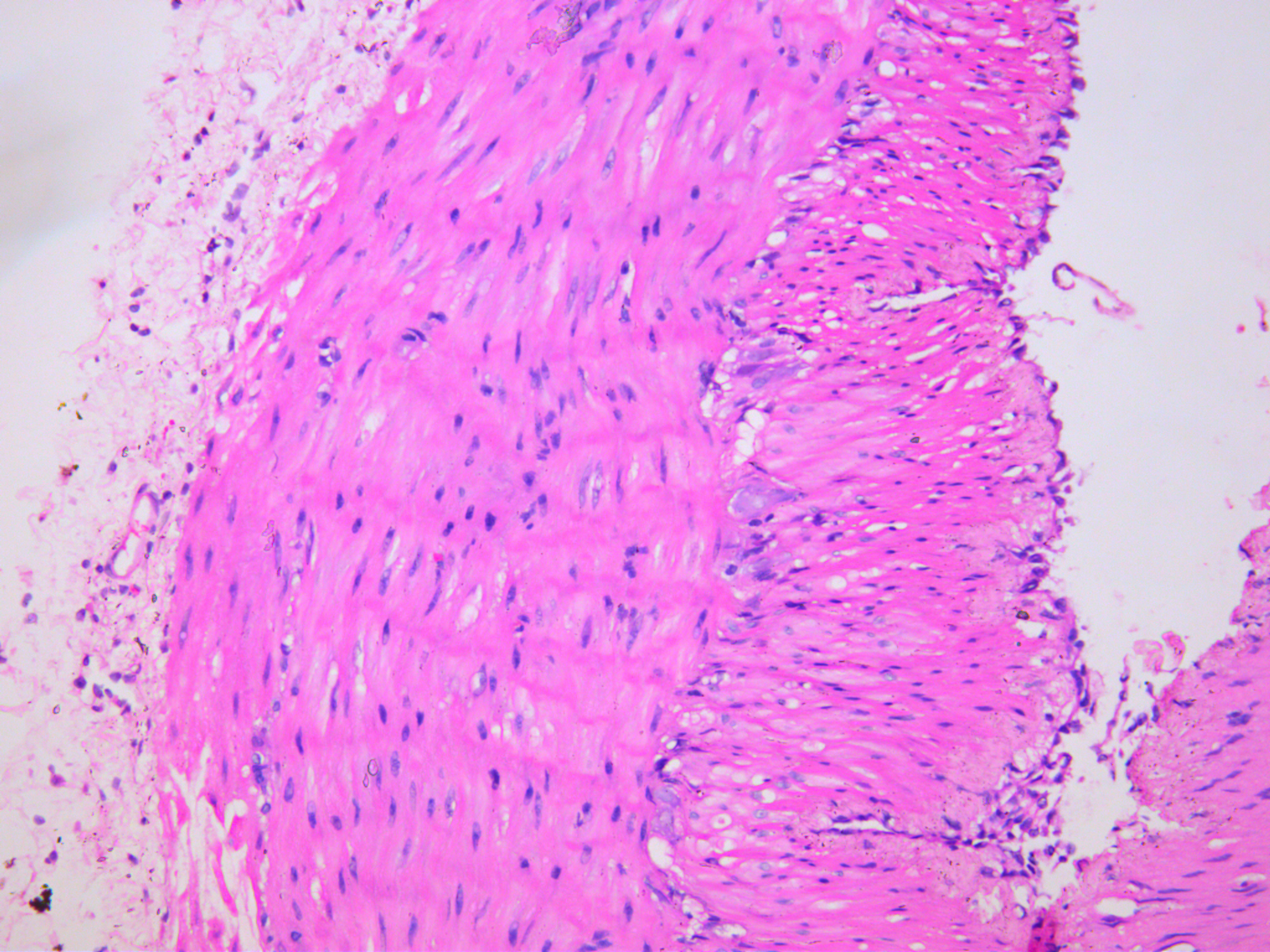

Supplement: Supplementary file 1 [file biomedicines-13-02564-s001.zip › Fig 5 D x20 .tif]

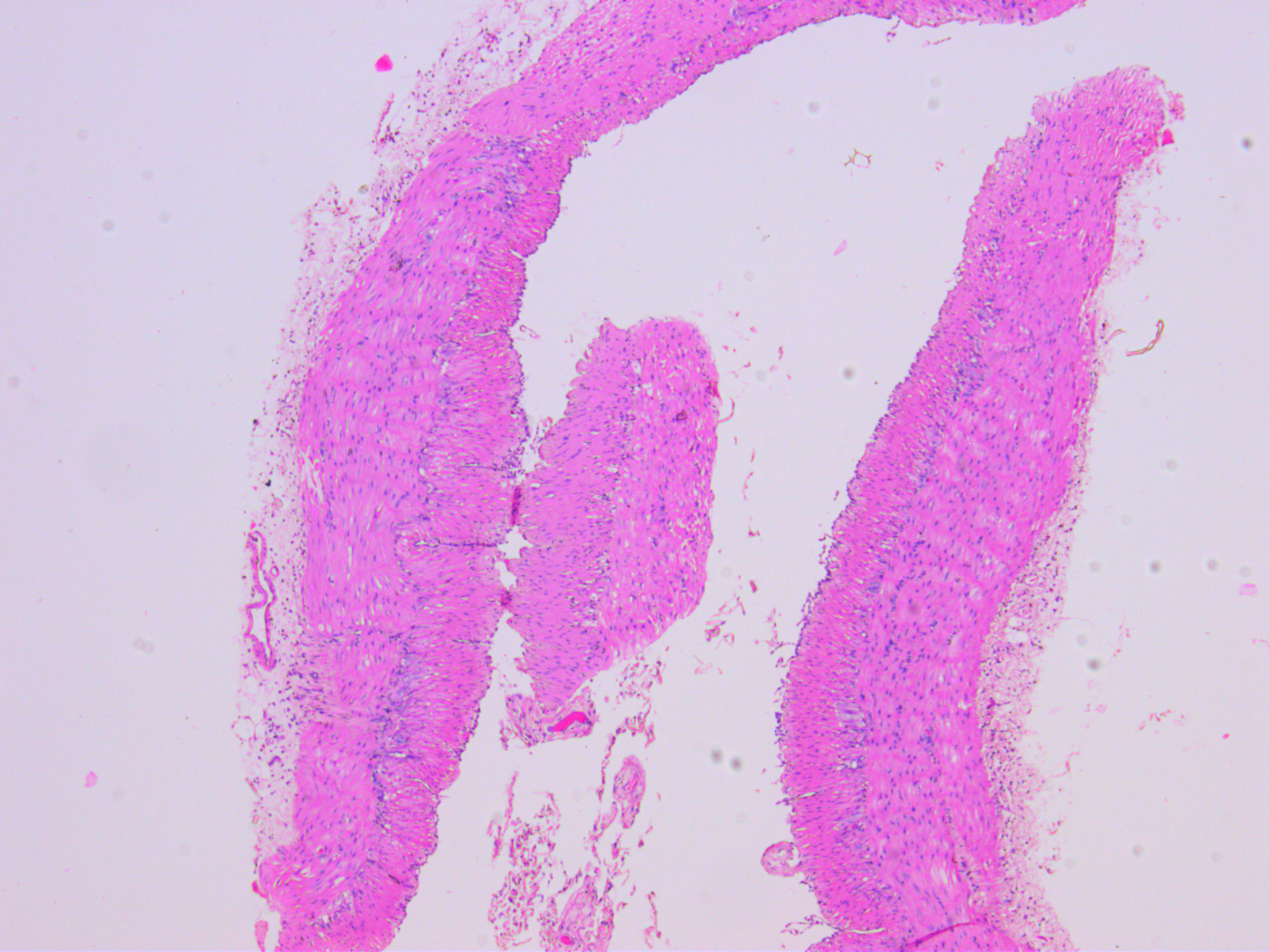

Supplement: Supplementary file 1 [file biomedicines-13-02564-s001.zip › Fig 5 D x5 .tif]

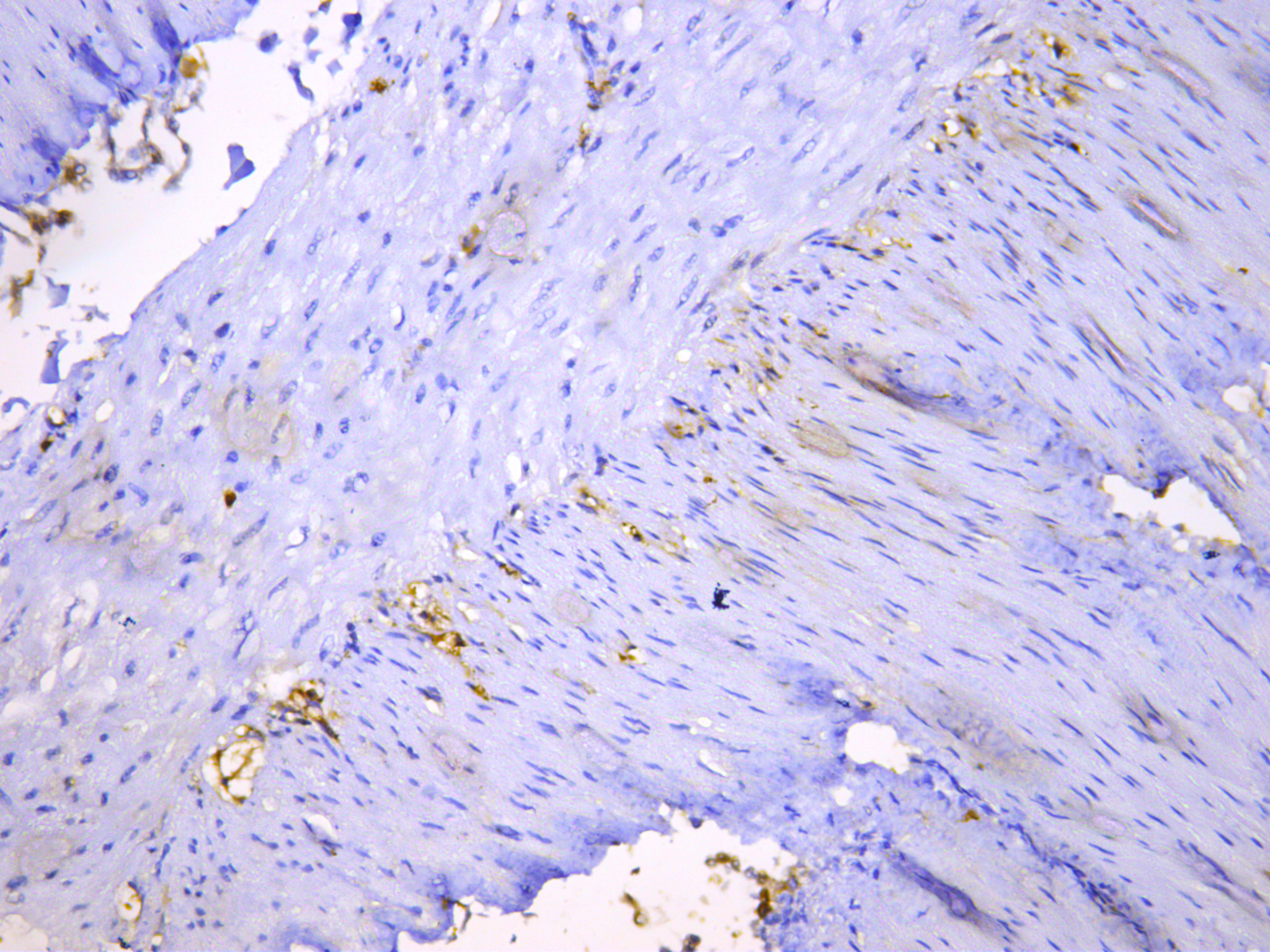

Supplement: Supplementary file 1 [file biomedicines-13-02564-s001.zip › Fig 5 E x20 .tif]

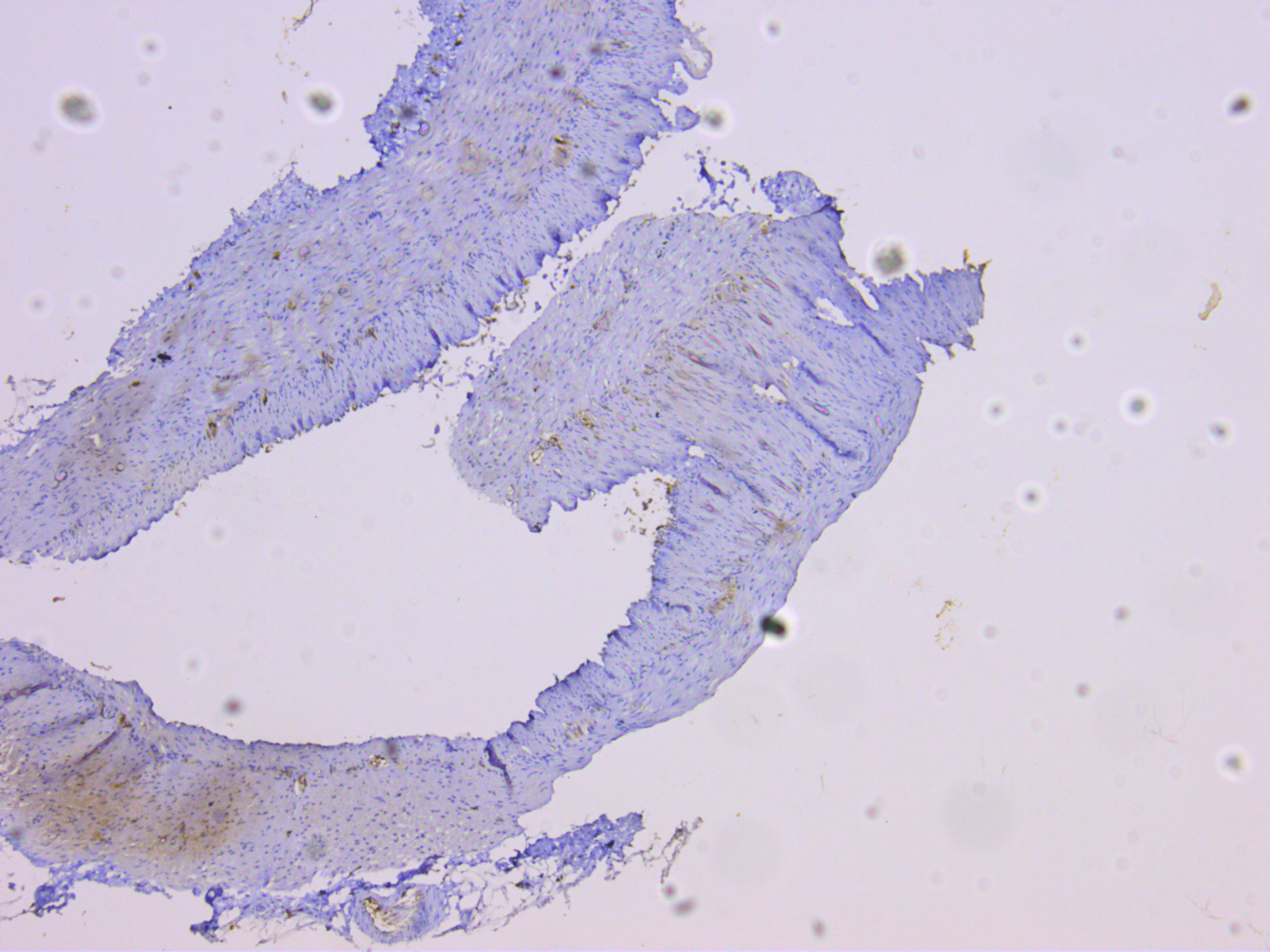

Supplement: Supplementary file 1 [file biomedicines-13-02564-s001.zip › Fig 5 E x5 .tif]

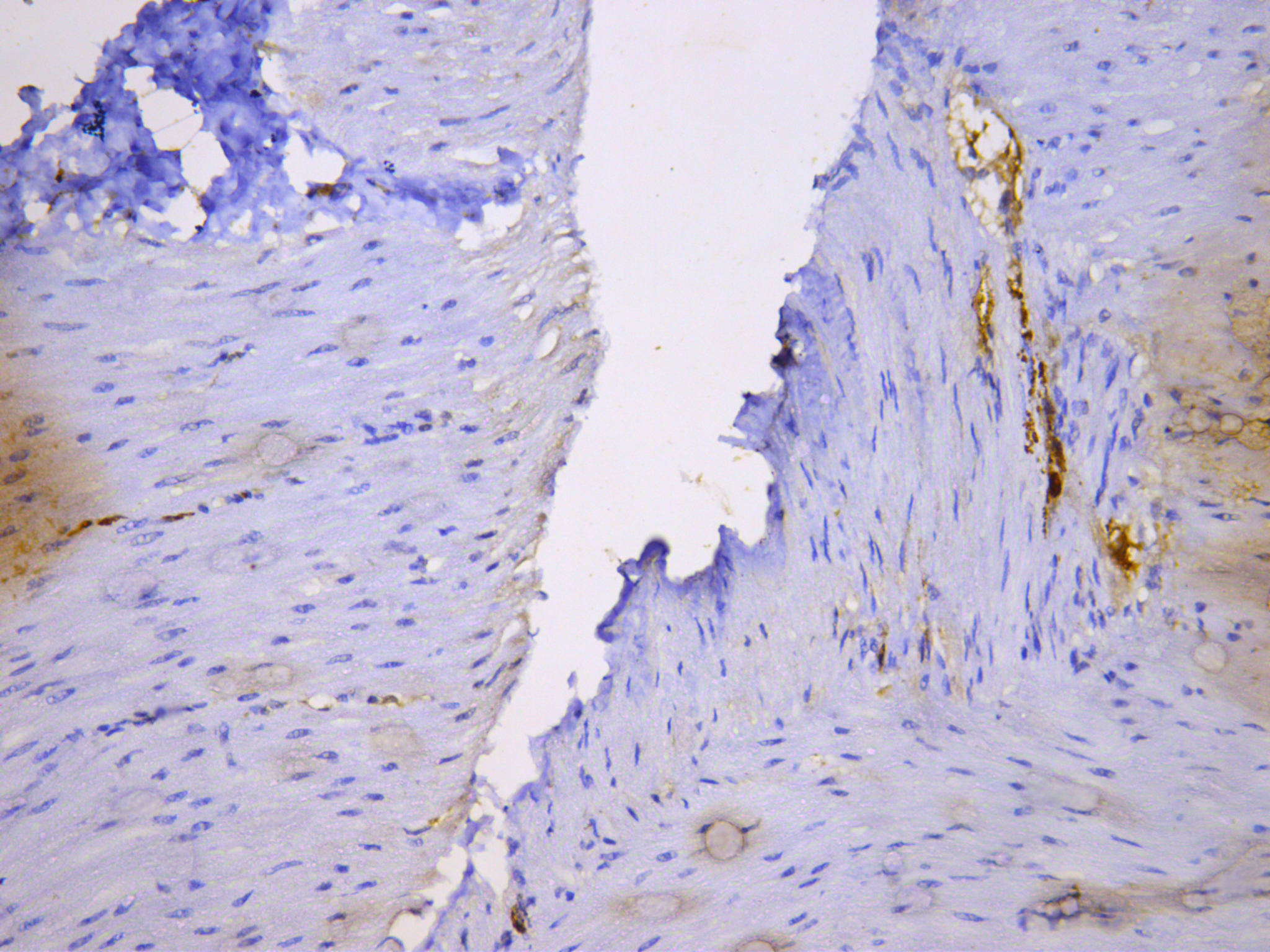

Supplement: Supplementary file 1 [file biomedicines-13-02564-s001.zip › Fig 5 F x20 .tif]

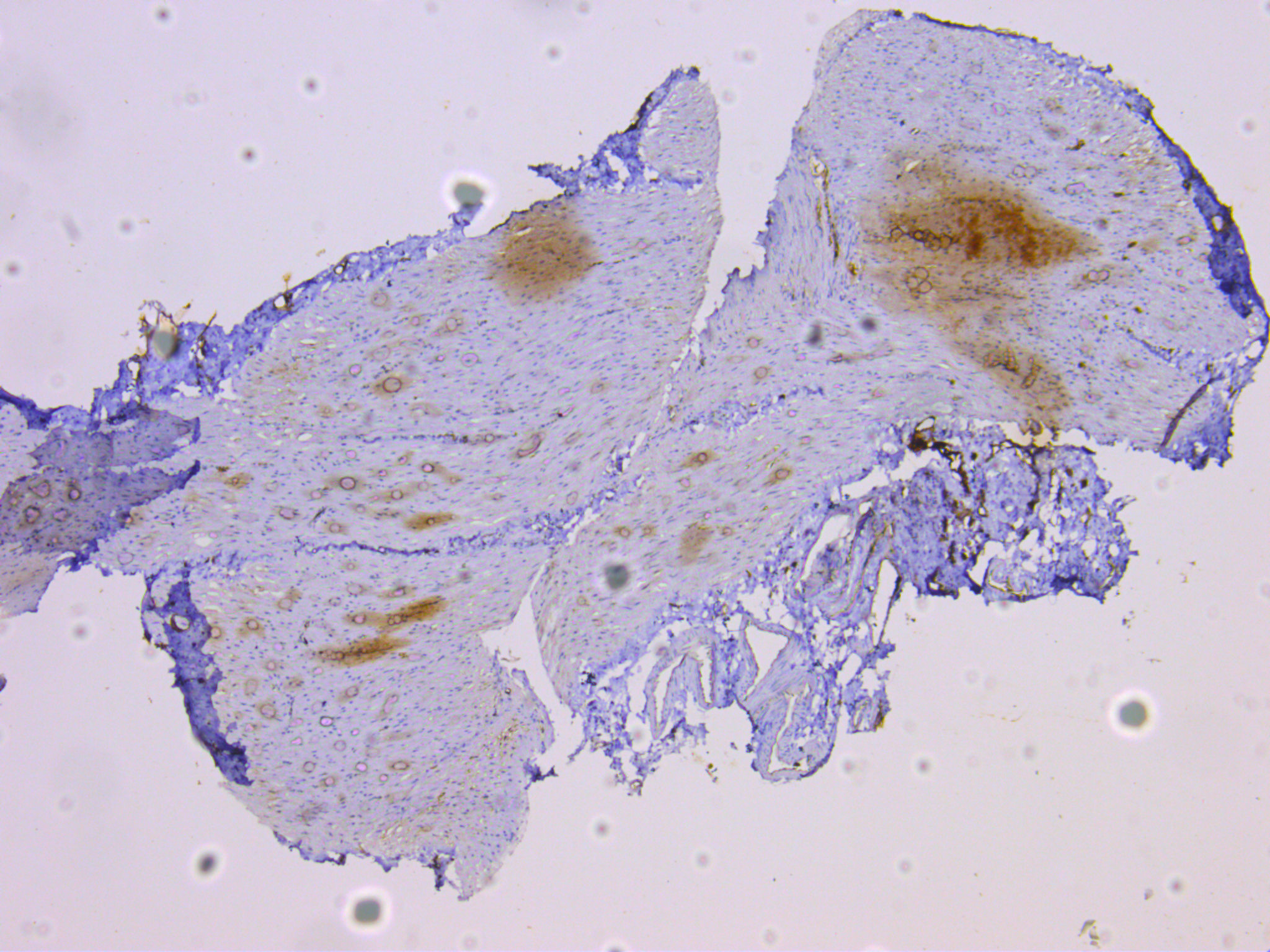

Supplement: Supplementary file 1 [file biomedicines-13-02564-s001.zip › Fig 5 F x5 .tif]

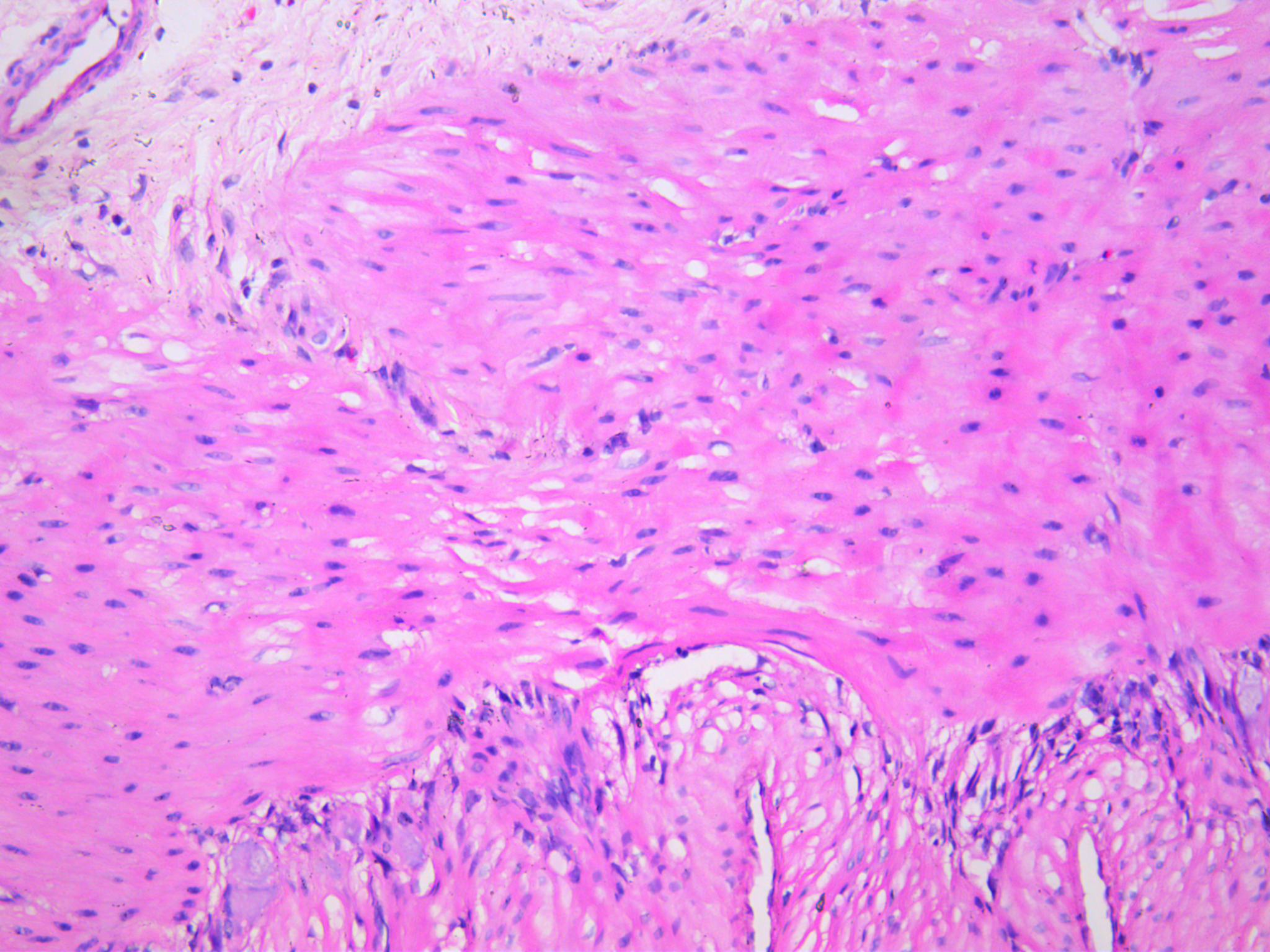

Supplement: Supplementary file 1 [file biomedicines-13-02564-s001.zip › Fig 5 G x20 .tif]

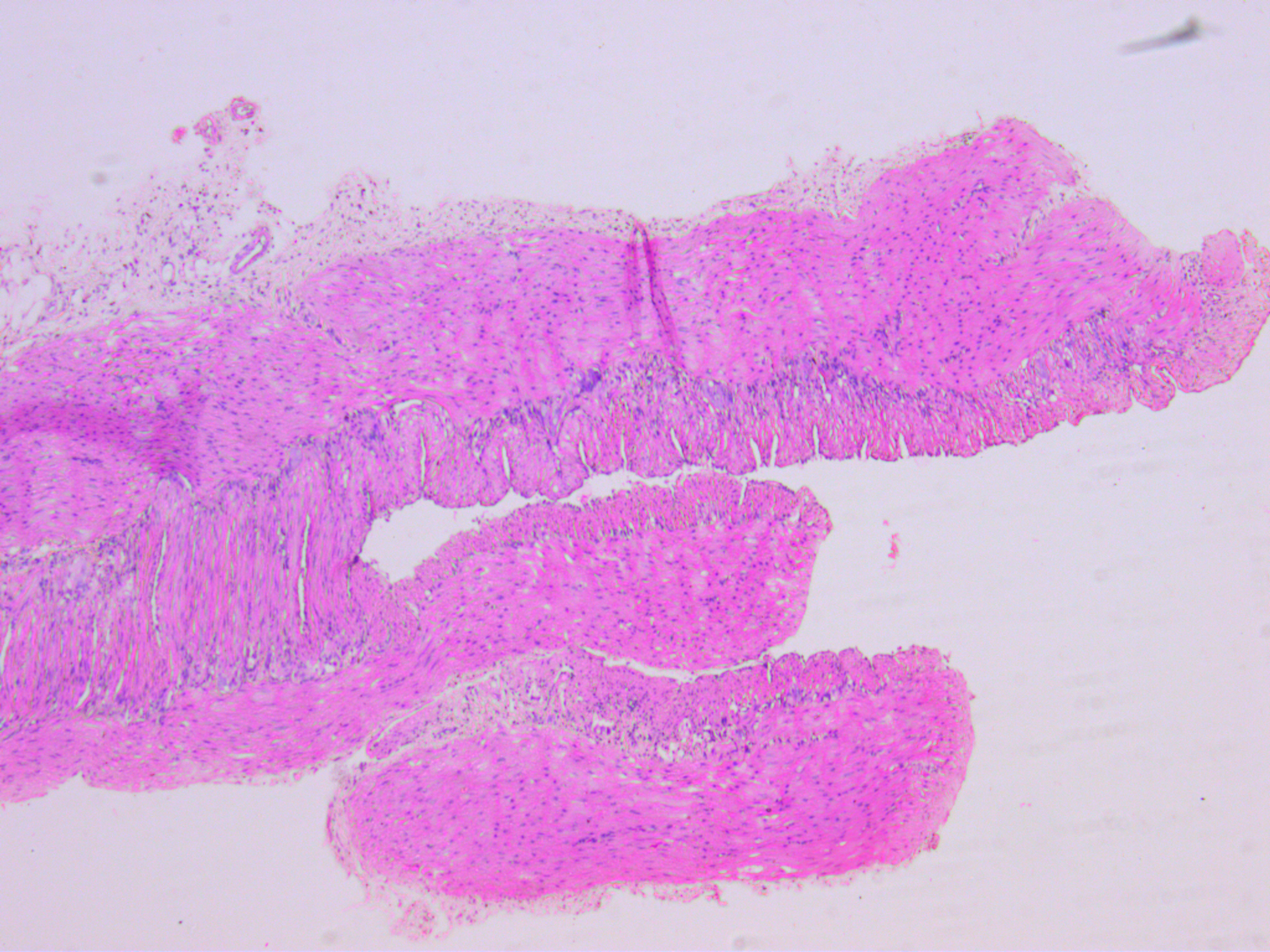

Supplement: Supplementary file 1 [file biomedicines-13-02564-s001.zip › Fig 5 G x5 .tif]

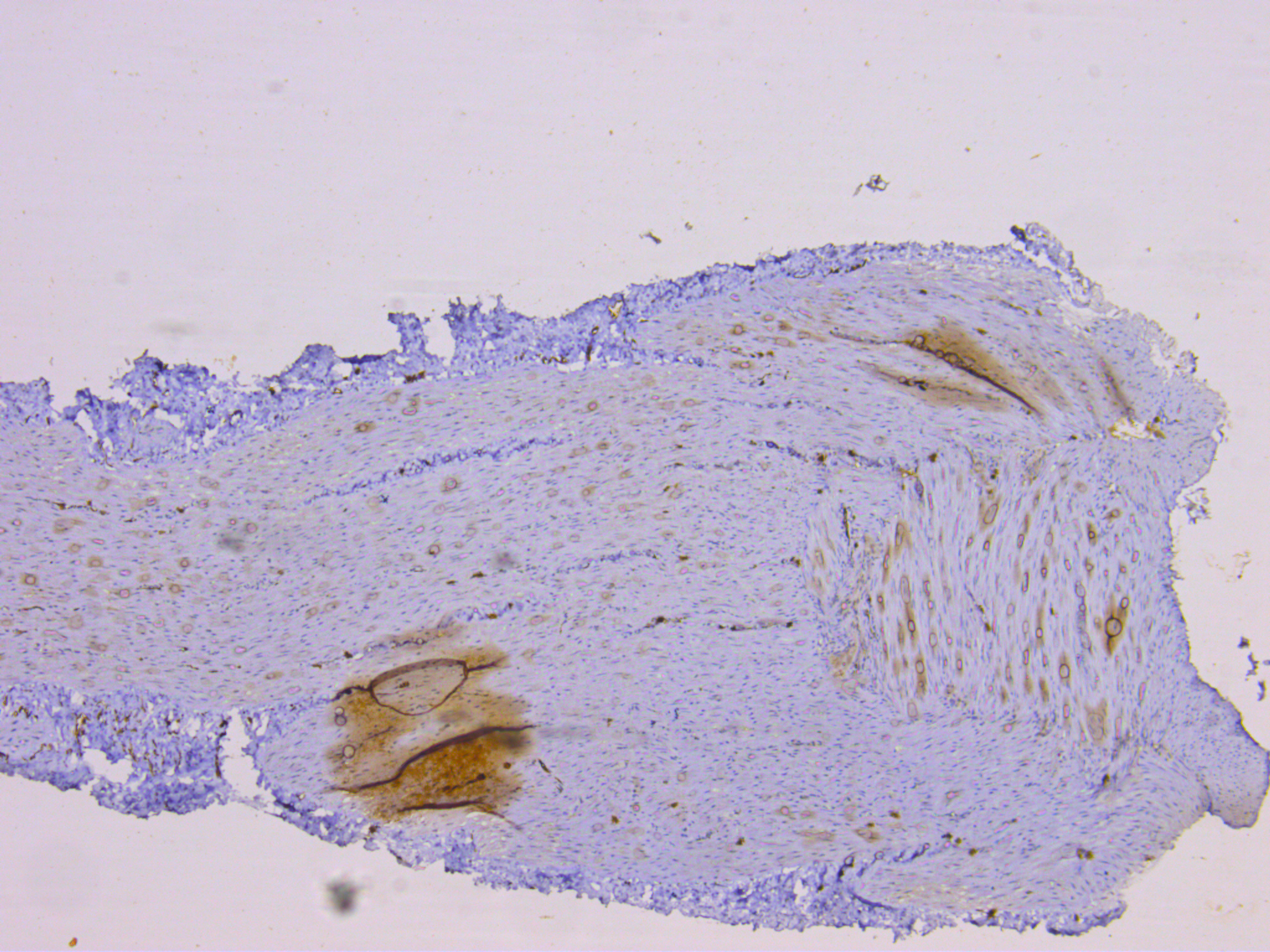

Supplement: Supplementary file 1 [file biomedicines-13-02564-s001.zip › Fig 5 H x5 .tif]

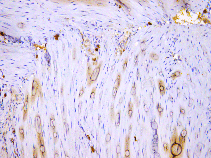

Supplement: Supplementary file 1 [file biomedicines-13-02564-s001.zip › Fig 5 Hx20.png]

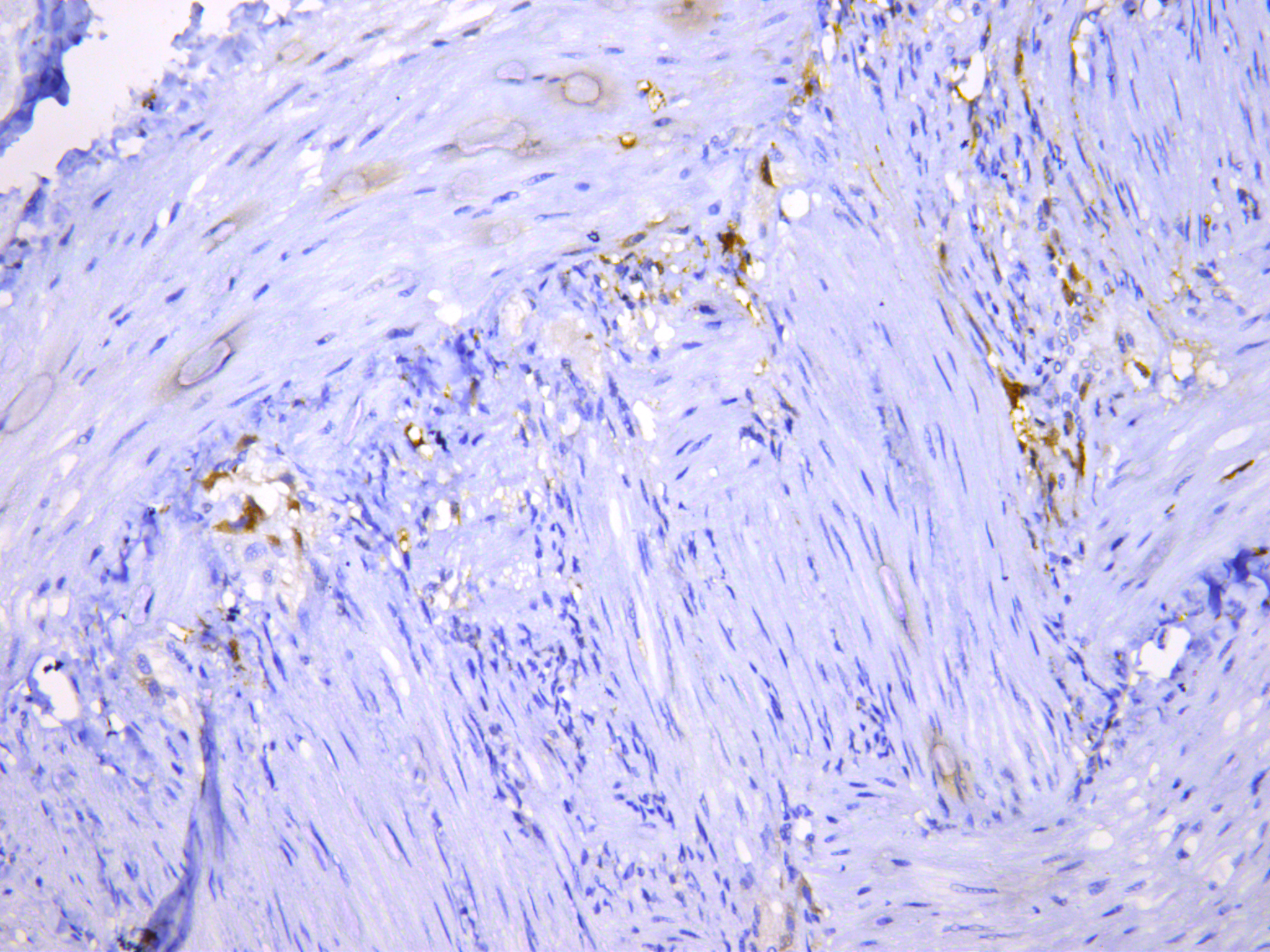

Supplement: Supplementary file 1 [file biomedicines-13-02564-s001.zip › Fig 5 I x20 .tif]

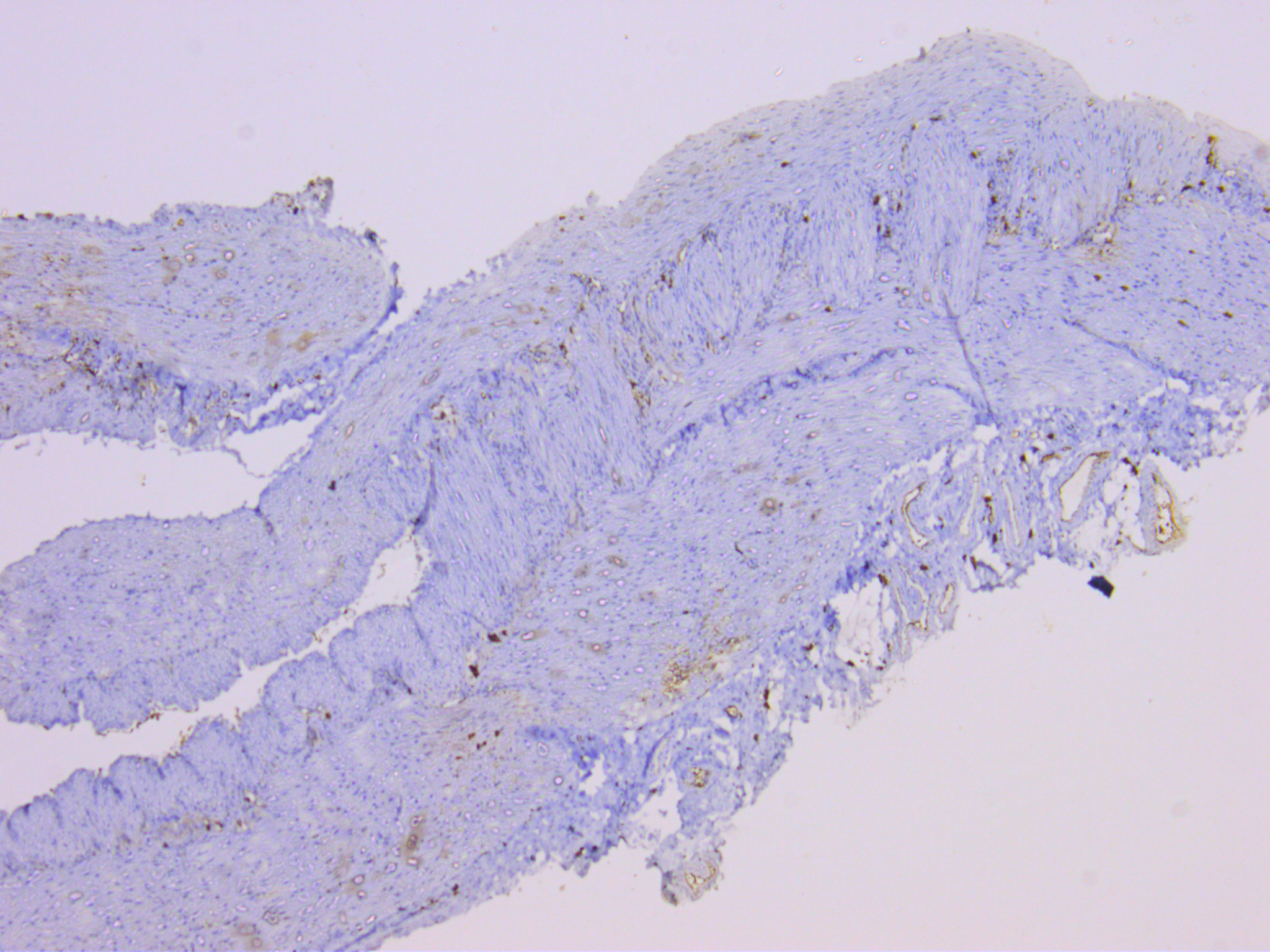

Supplement: Supplementary file 1 [file biomedicines-13-02564-s001.zip › Fig 5 I x5 .tif]

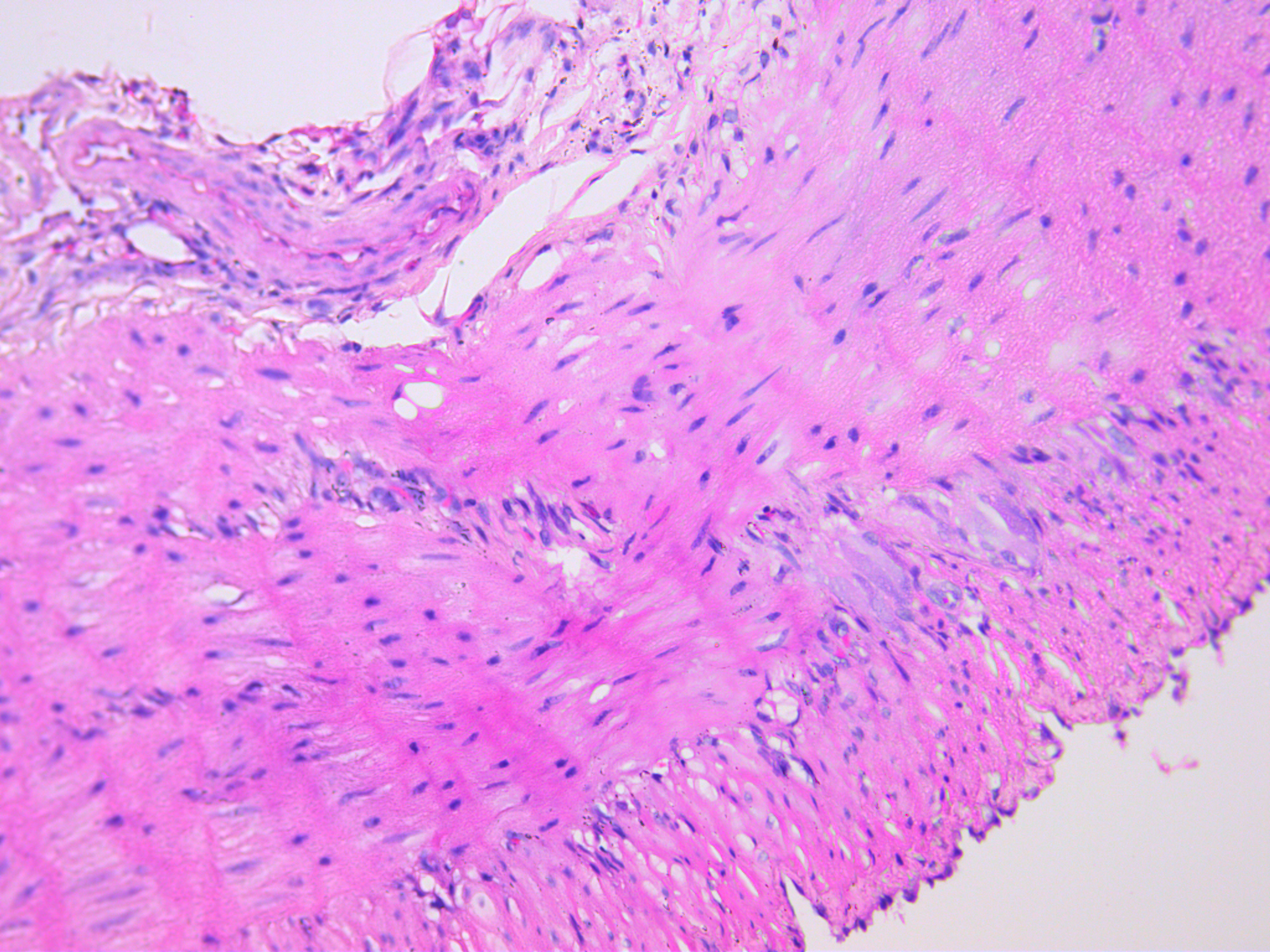

Supplement: Supplementary file 1 [file biomedicines-13-02564-s001.zip › Fig 5 J x20 .tif]

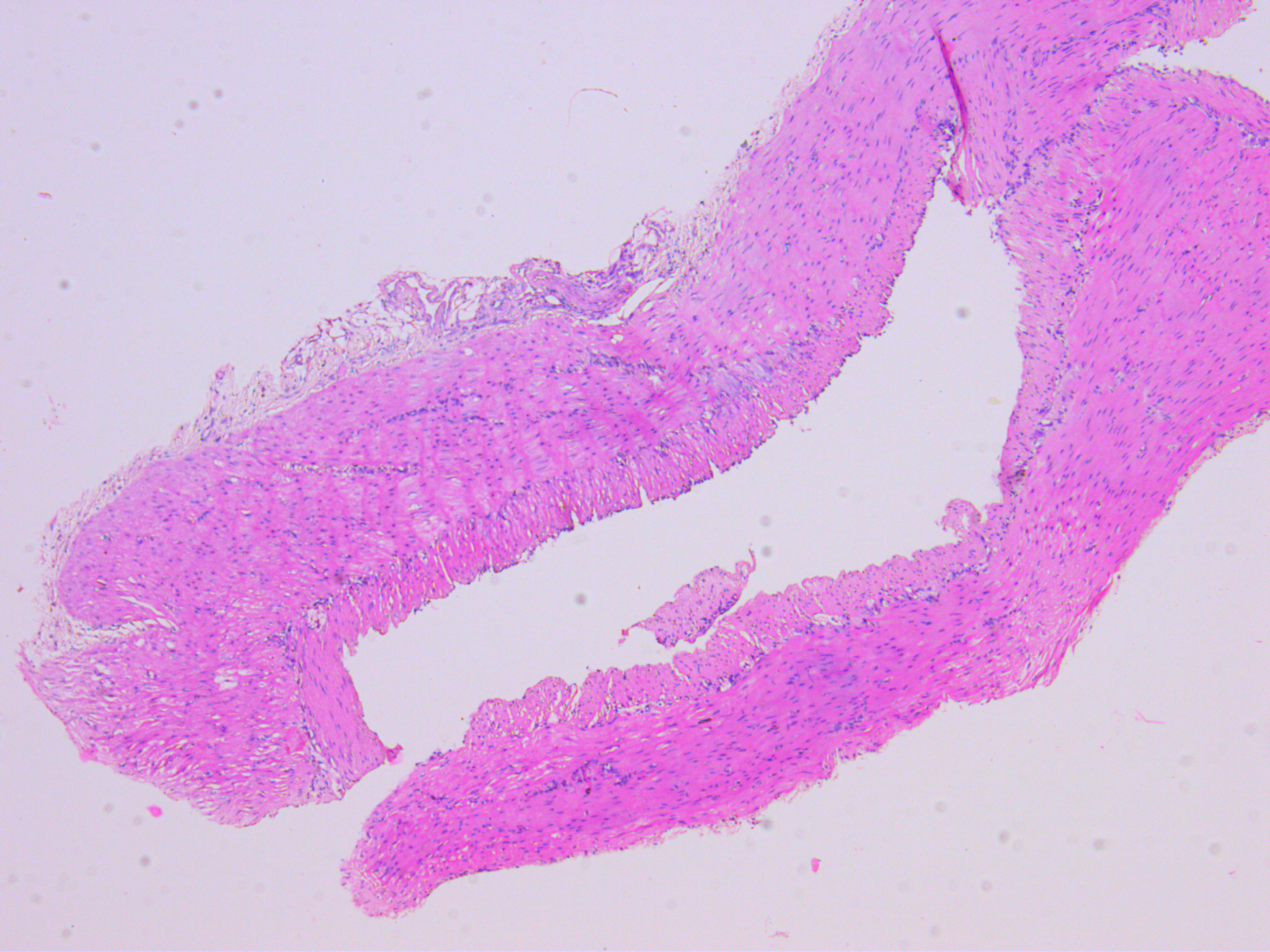

Supplement: Supplementary file 1 [file biomedicines-13-02564-s001.zip › Fig 5 J x5 .tif]

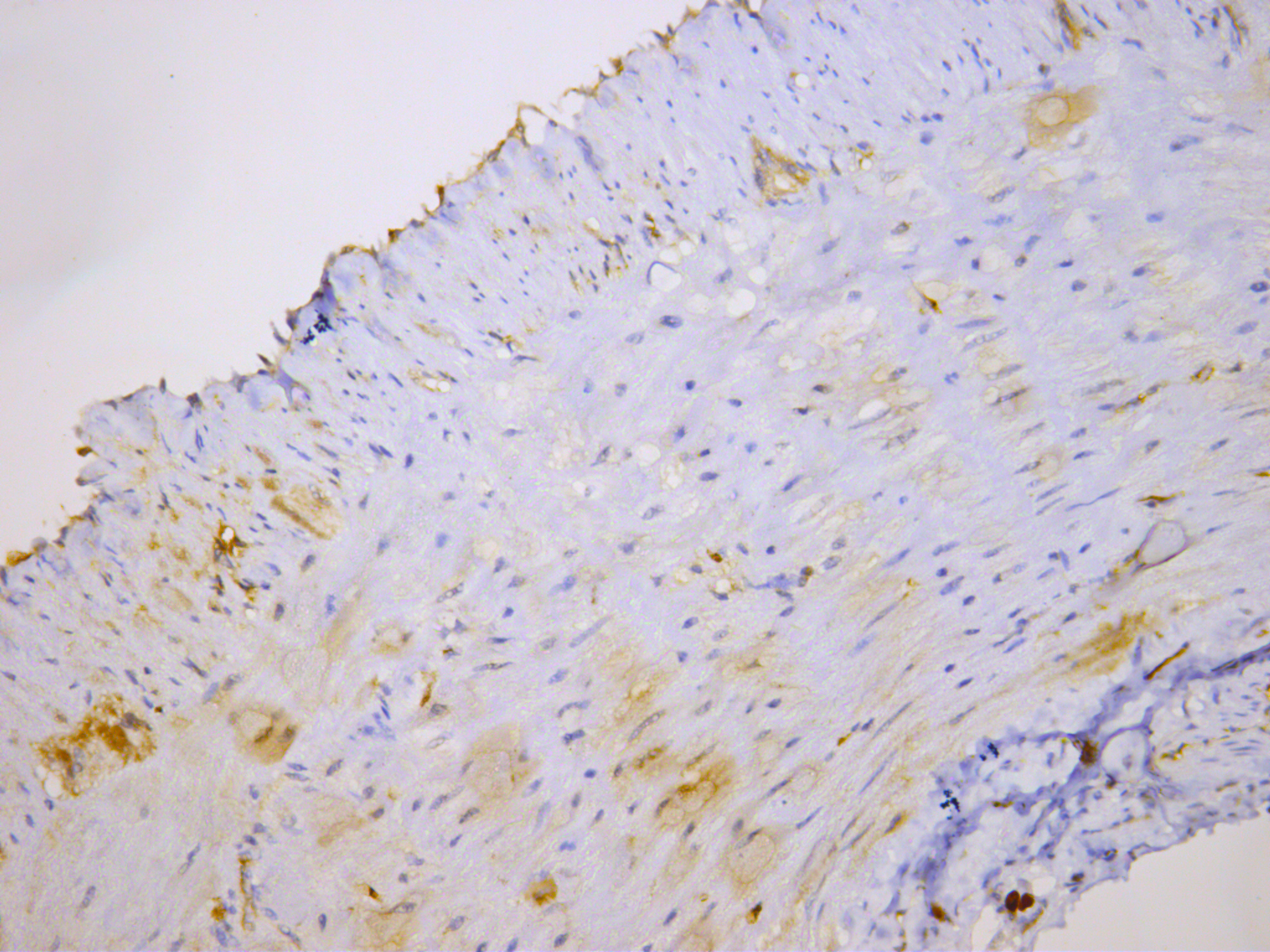

Supplement: Supplementary file 1 [file biomedicines-13-02564-s001.zip › Fig 5 K x20 .tif]

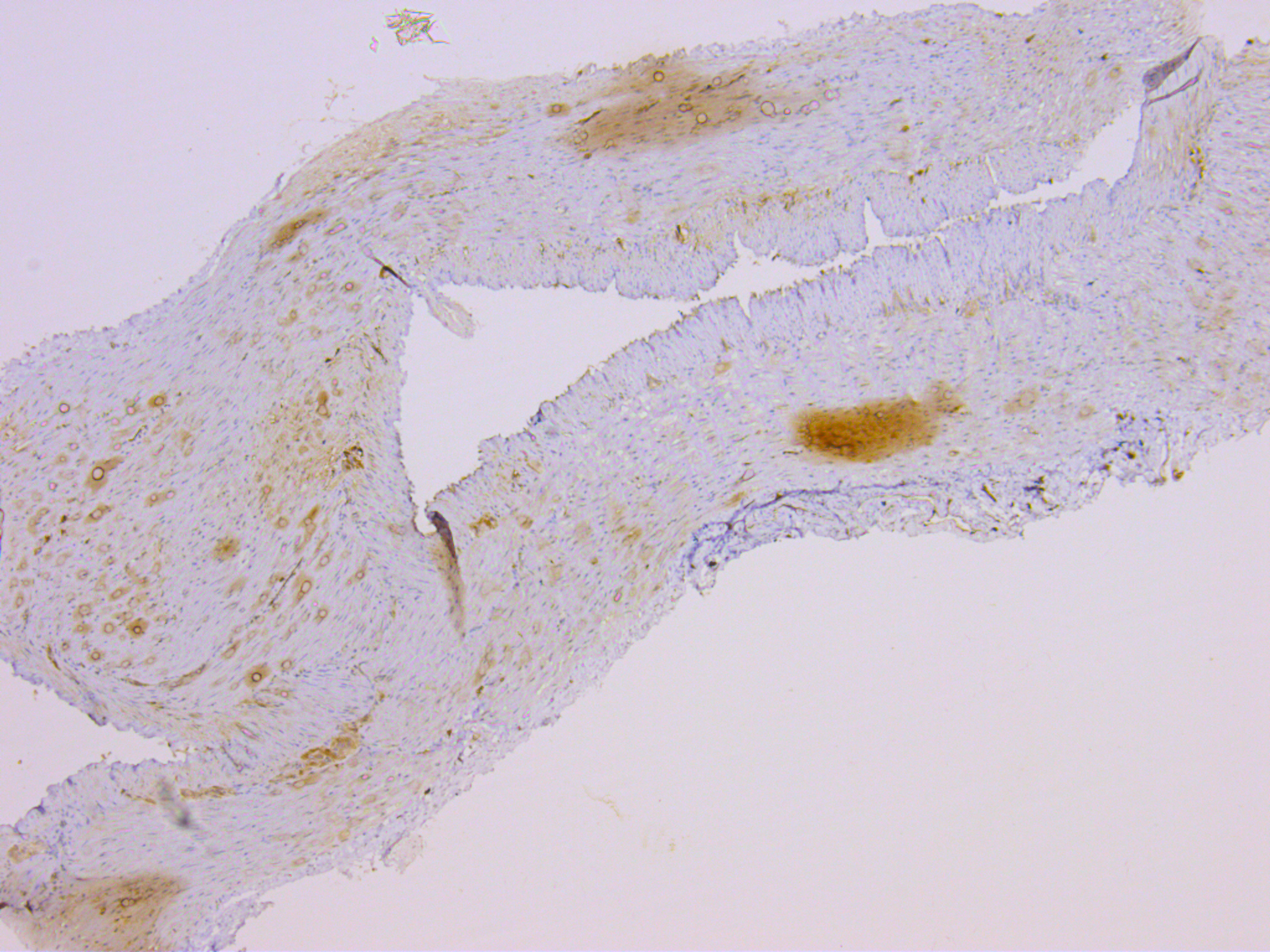

Supplement: Supplementary file 1 [file biomedicines-13-02564-s001.zip › Fig 5 K x5 .tif]

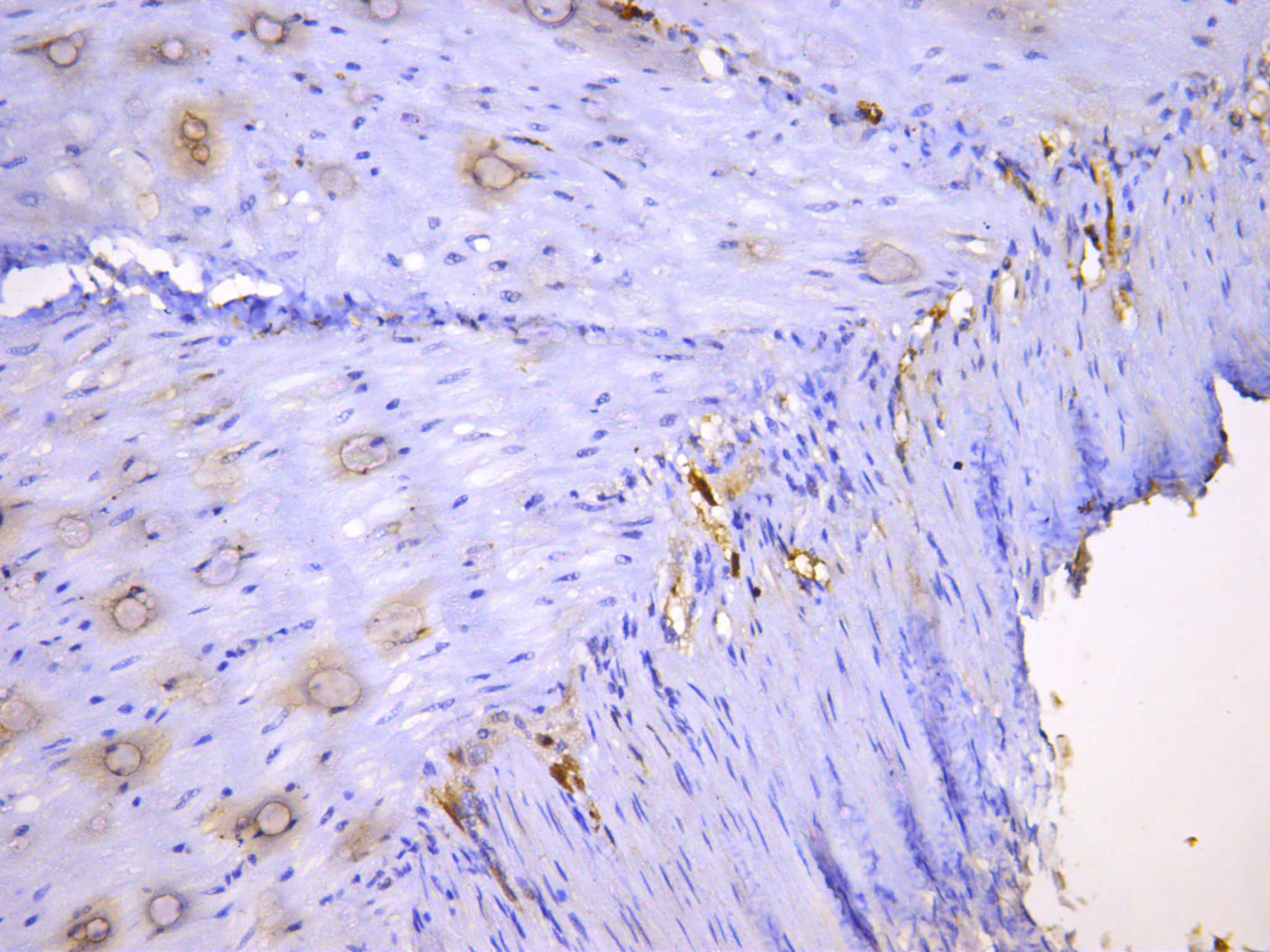

Supplement: Supplementary file 1 [file biomedicines-13-02564-s001.zip › Fig 5 L x20 .tif]

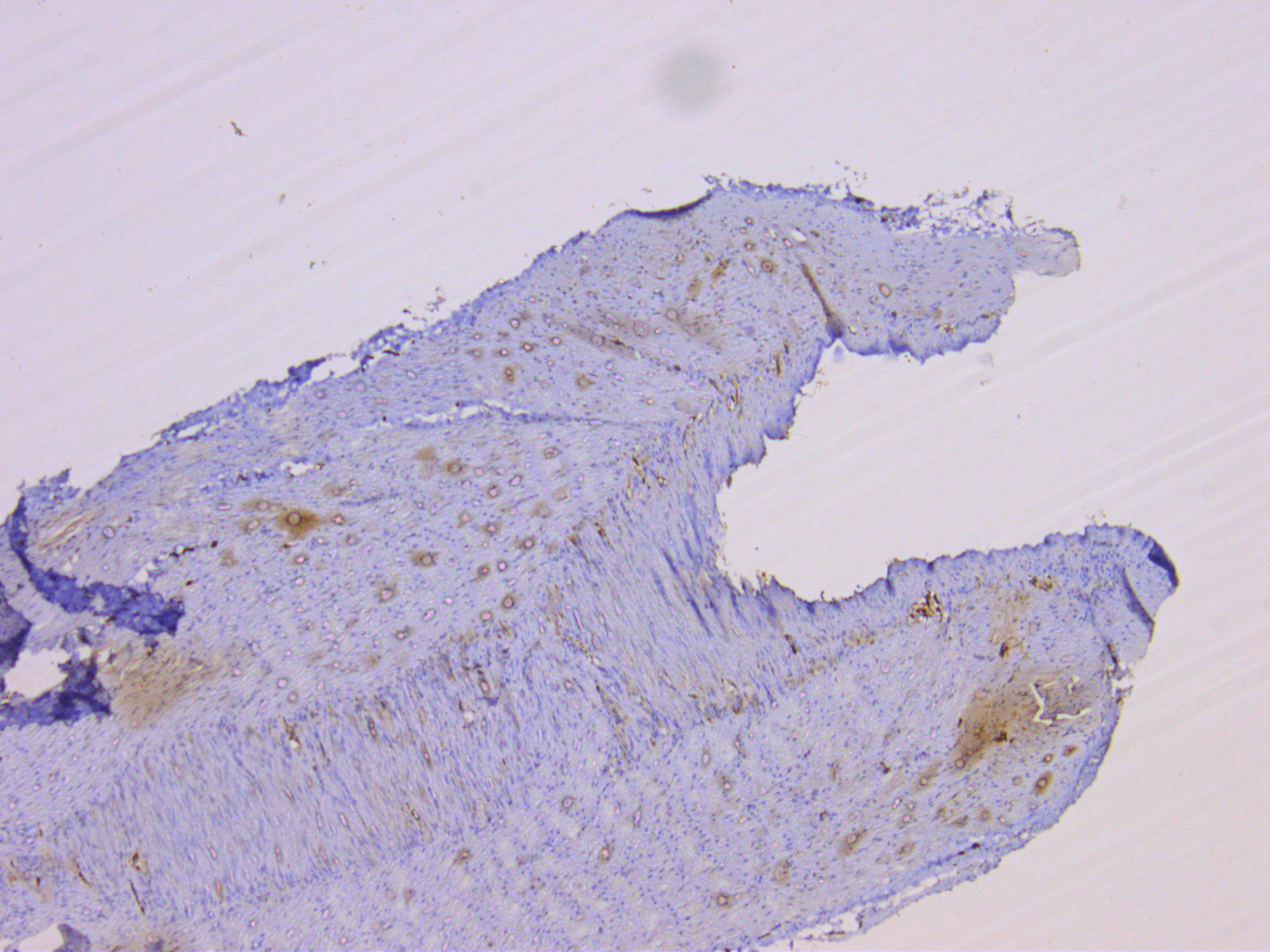

Supplement: Supplementary file 1 [file biomedicines-13-02564-s001.zip › Fig 5 L x5 .tif]

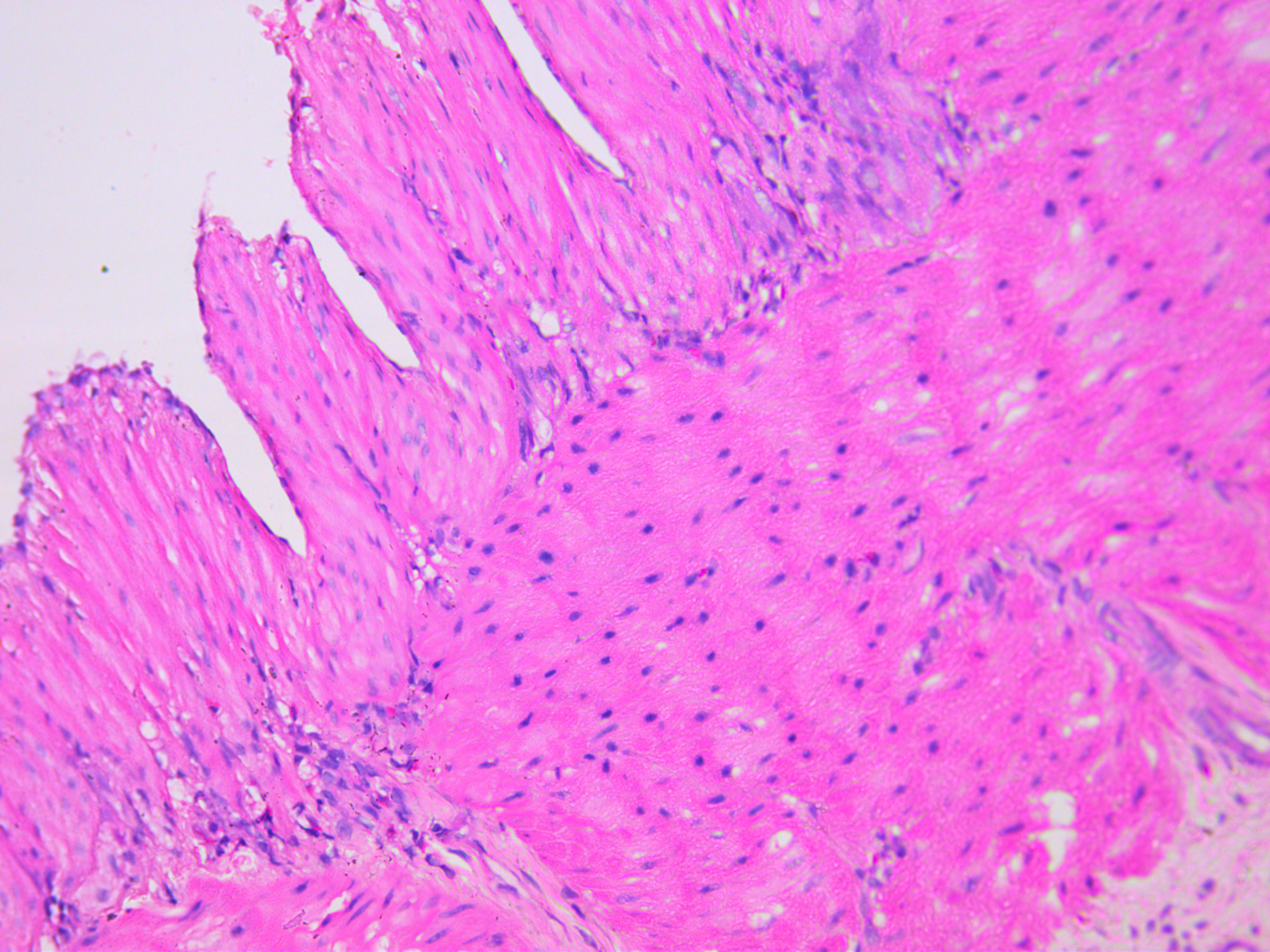

Supplement: Supplementary file 1 [file biomedicines-13-02564-s001.zip › Fig 5 M x20 .tif]

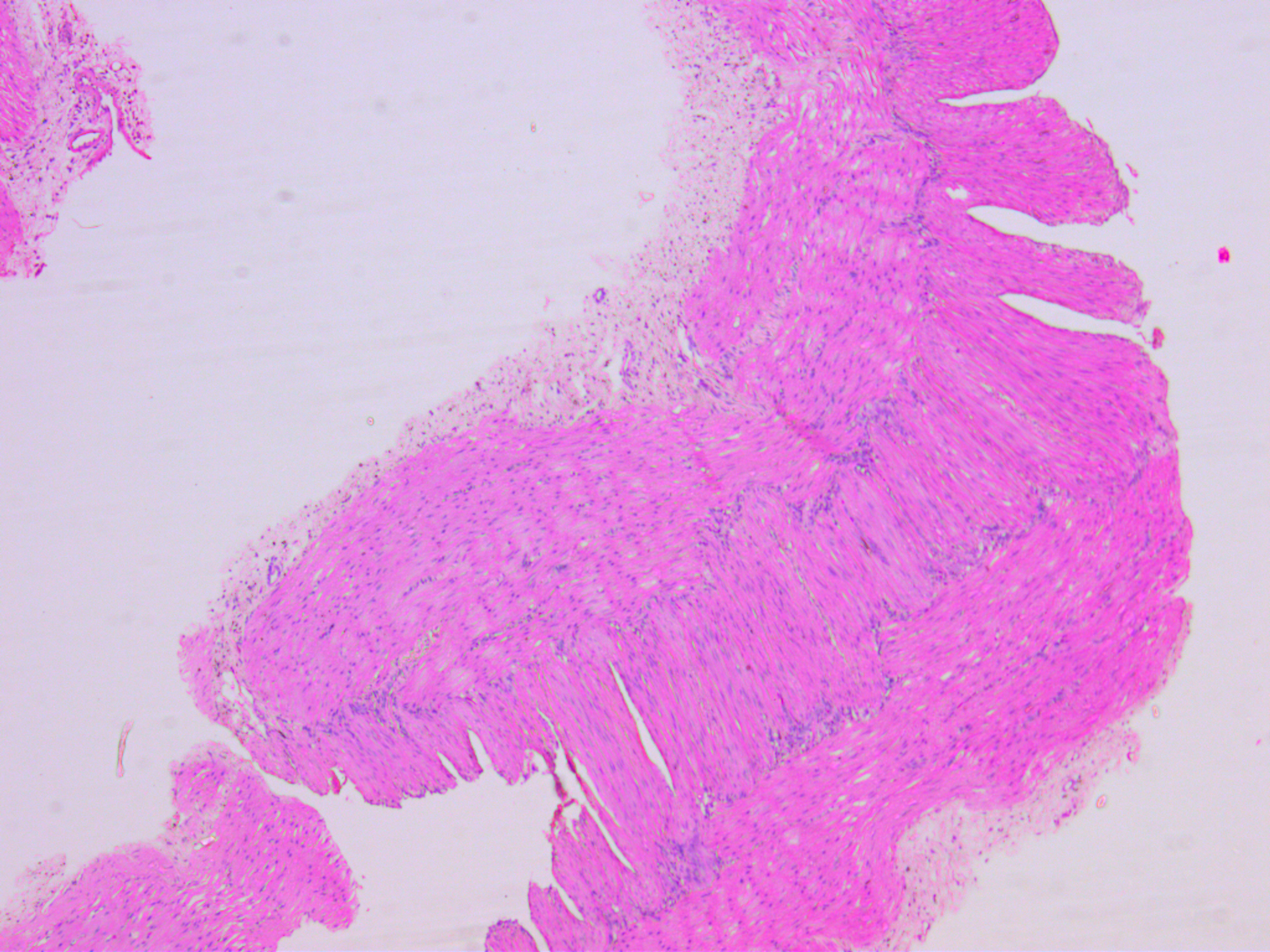

Supplement: Supplementary file 1 [file biomedicines-13-02564-s001.zip › Fig 5 M x5 .tif]

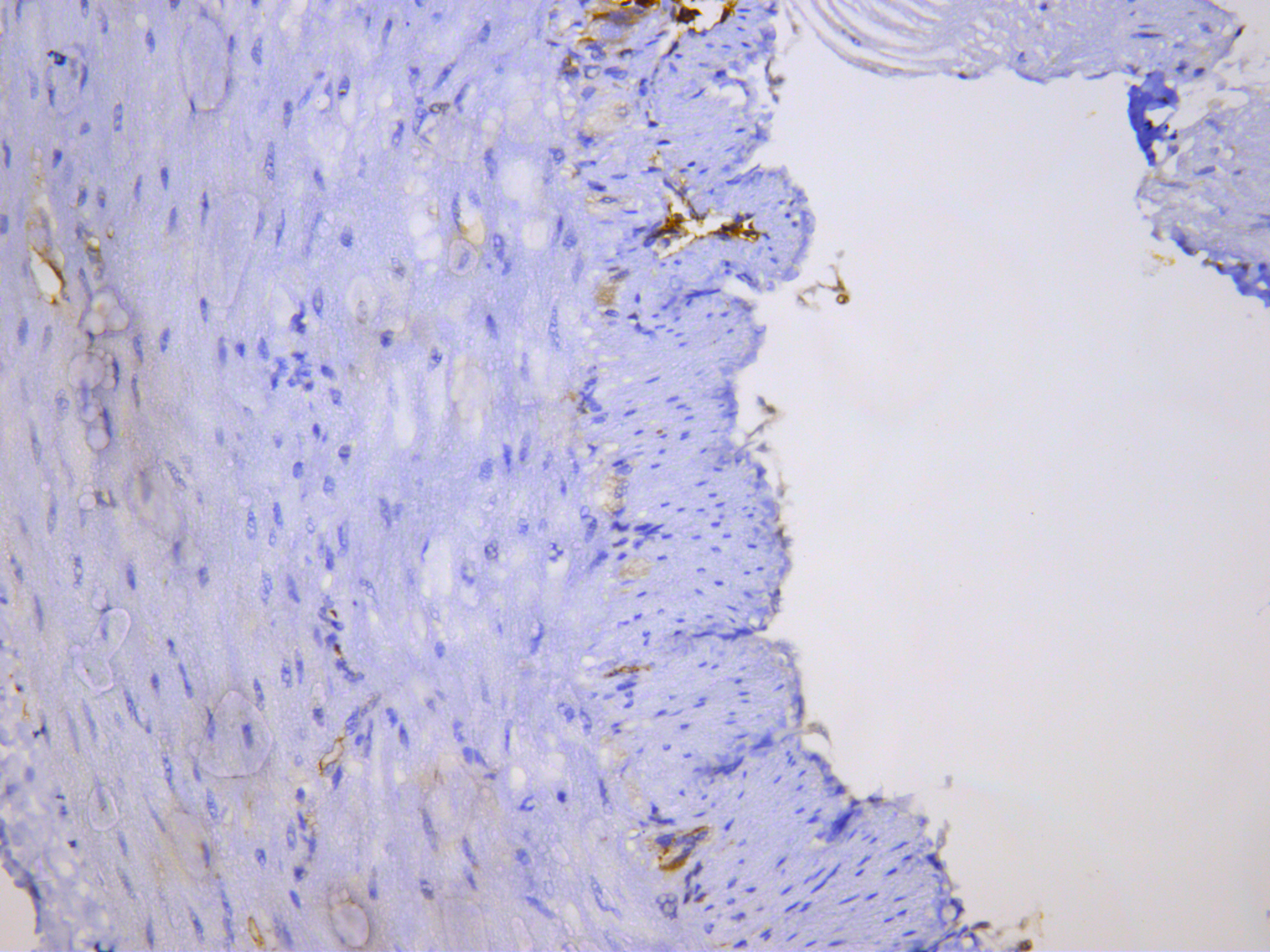

Supplement: Supplementary file 1 [file biomedicines-13-02564-s001.zip › Fig 5 N x20 .tif]

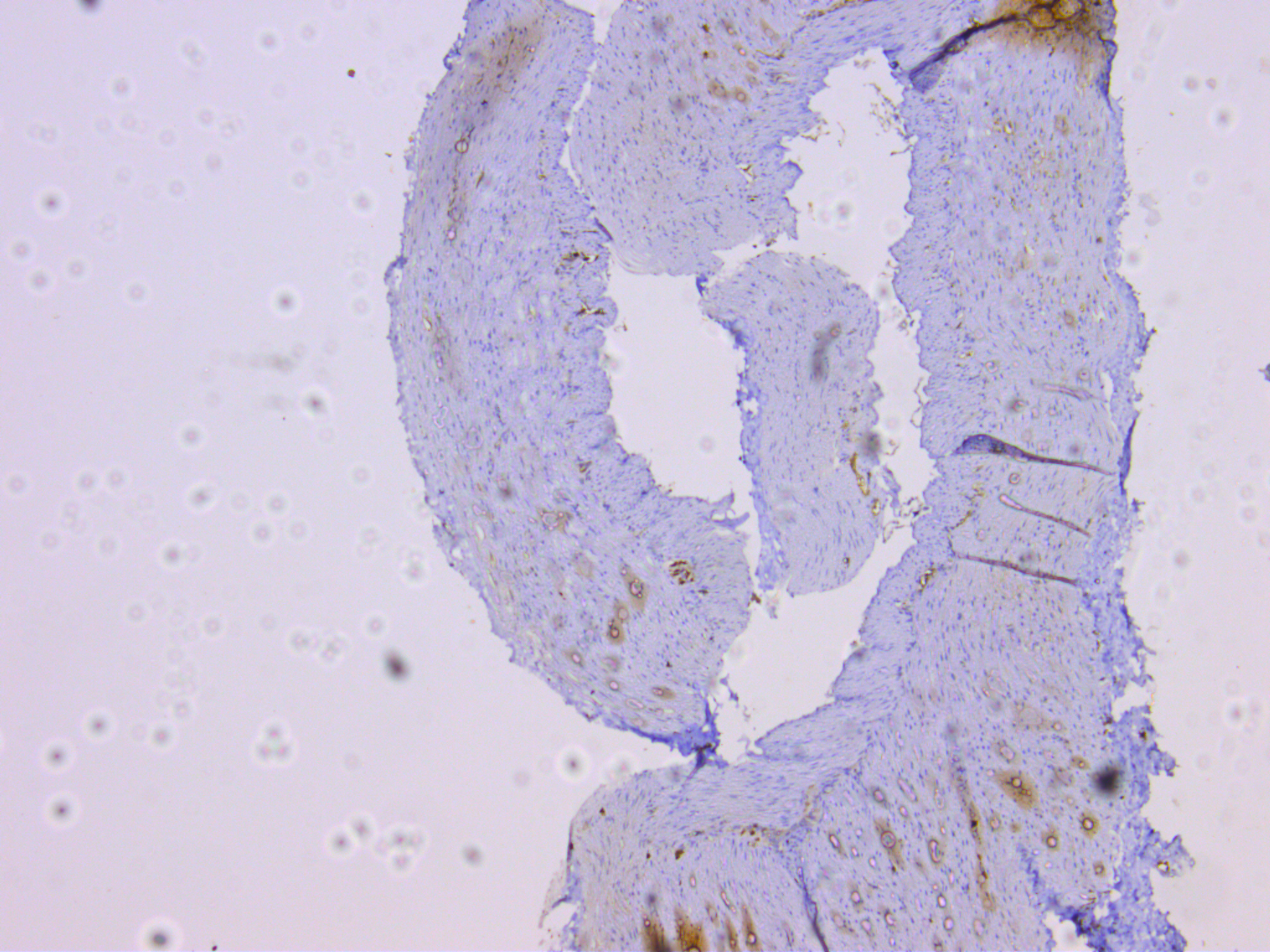

Supplement: Supplementary file 1 [file biomedicines-13-02564-s001.zip › Fig 5 N x5 .tif]

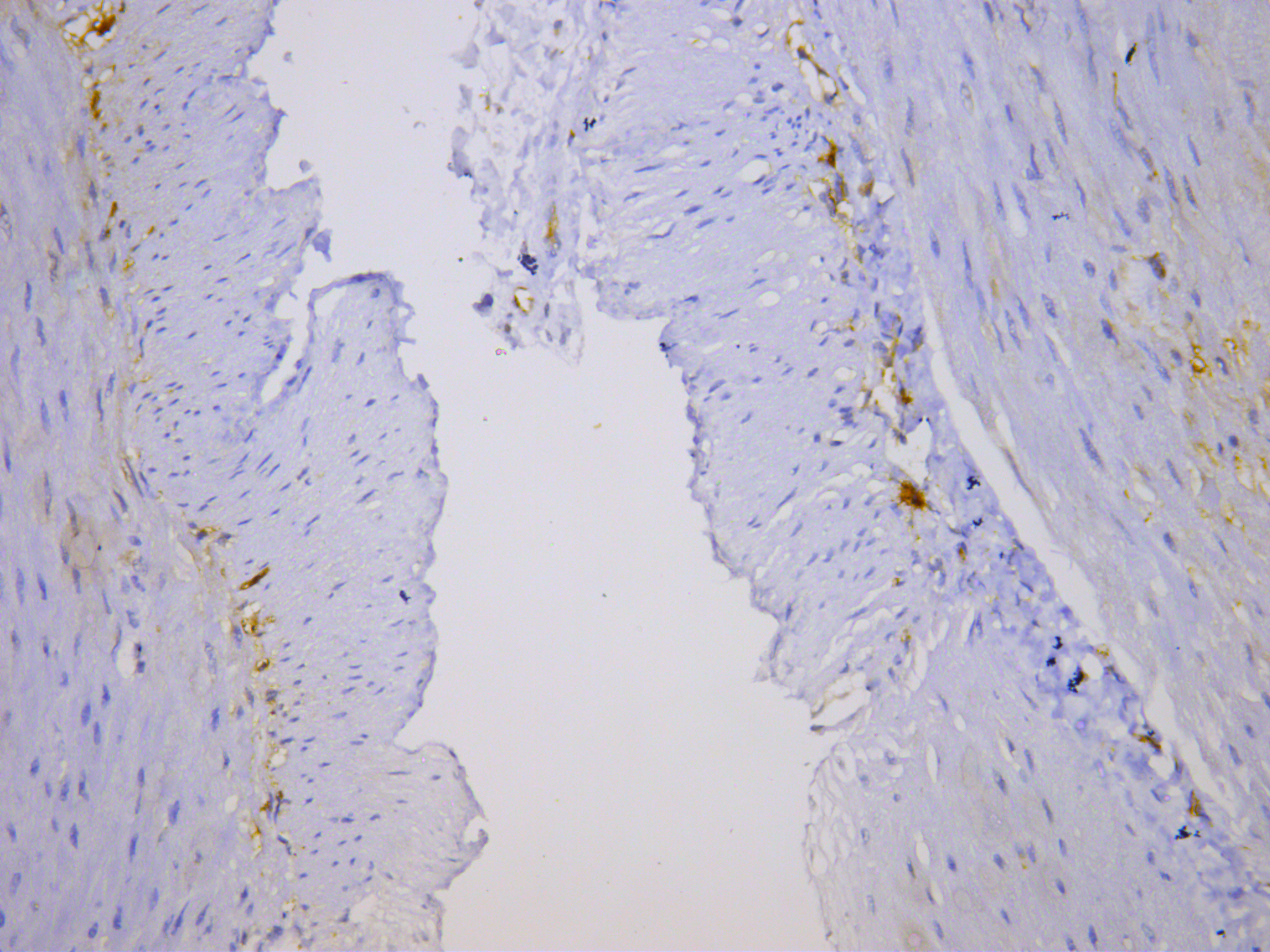

Supplement: Supplementary file 1 [file biomedicines-13-02564-s001.zip › Fig 5 O x20 .tif]

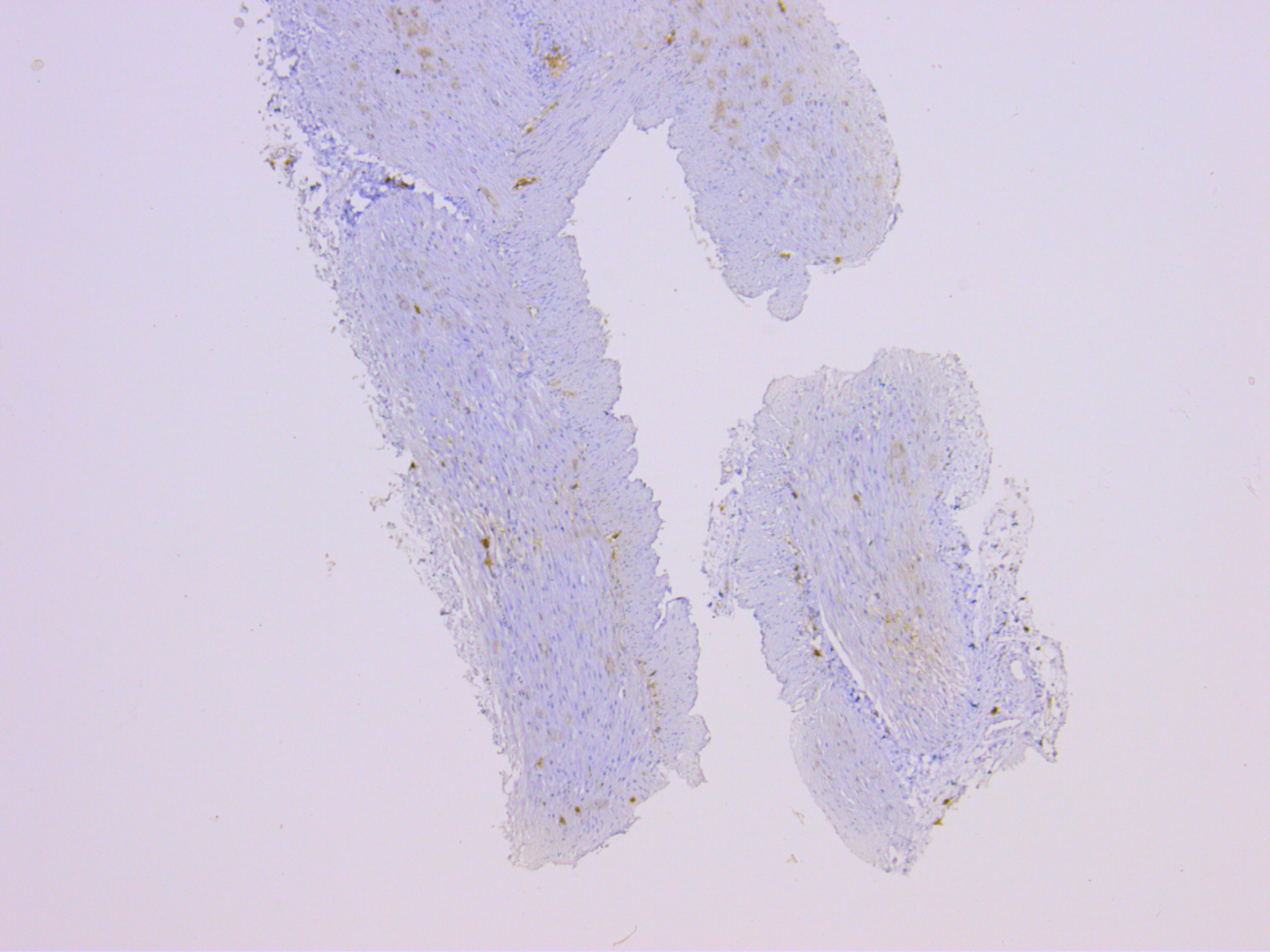

Supplement: Supplementary file 1 [file biomedicines-13-02564-s001.zip › Fig 5 O x5 .tif]

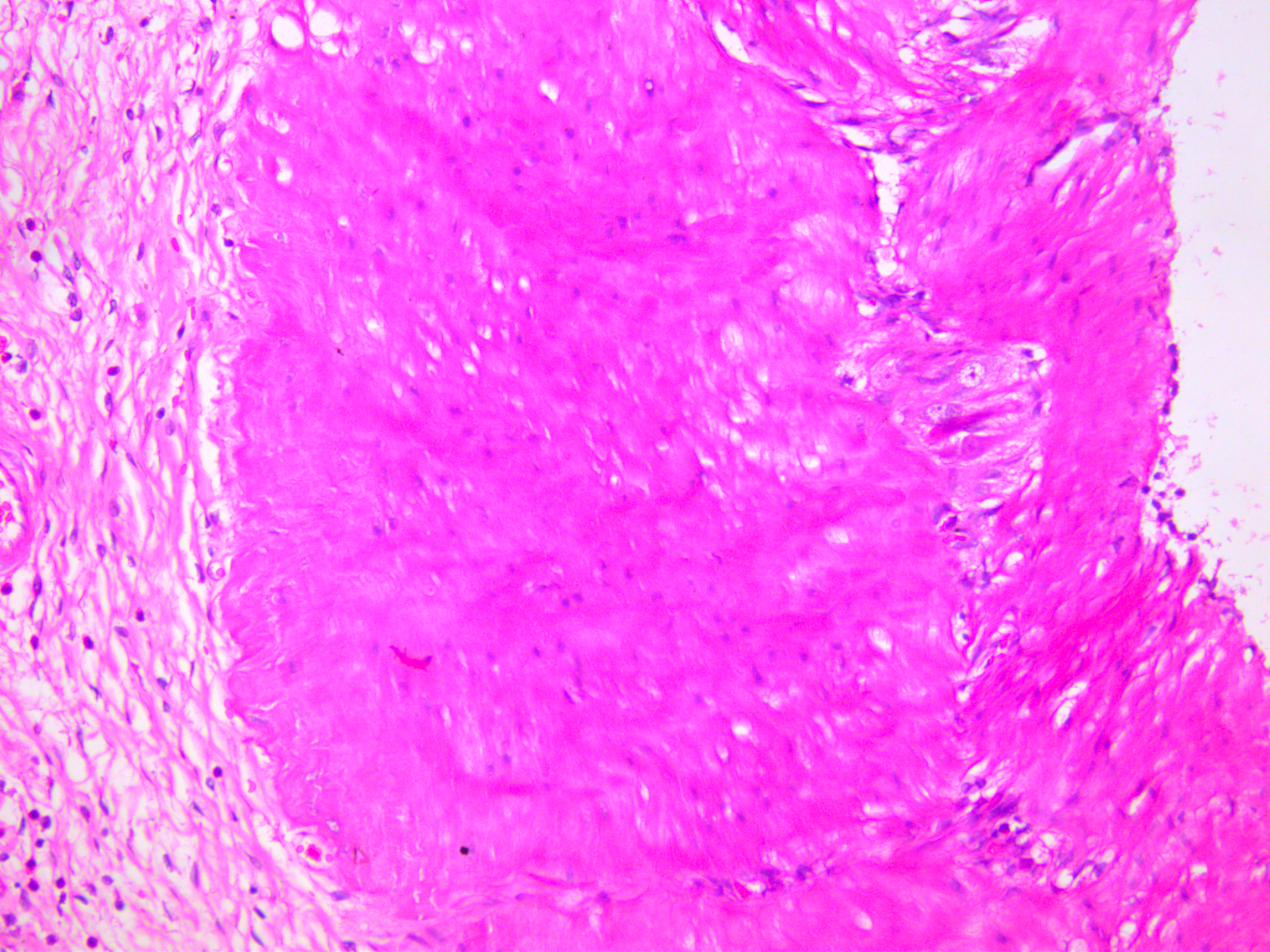

Supplement: Supplementary file 1 [file biomedicines-13-02564-s001.zip › Fig 5 P x20 .tif]

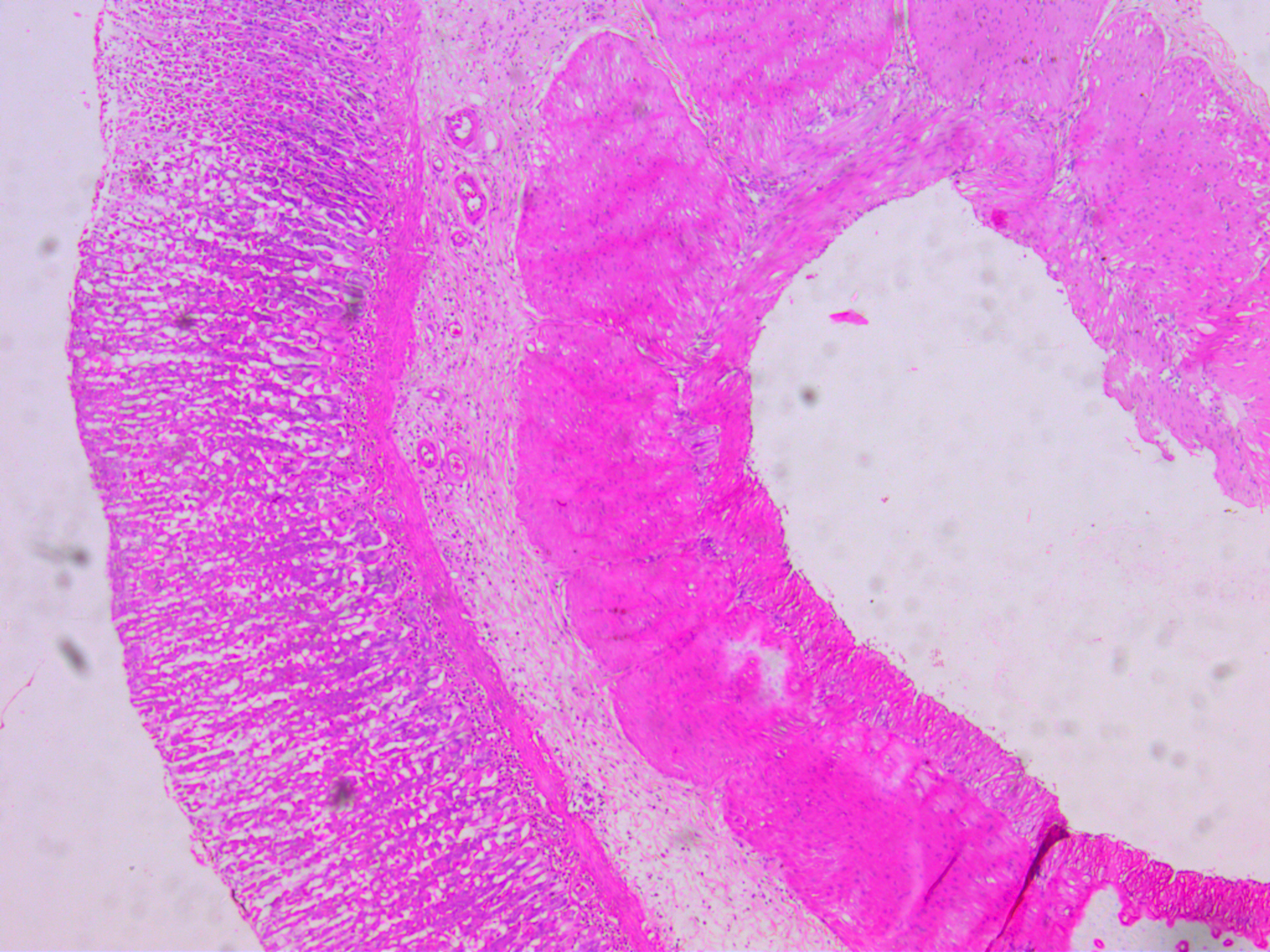

Supplement: Supplementary file 1 [file biomedicines-13-02564-s001.zip › Fig 5 P x5 .tif]

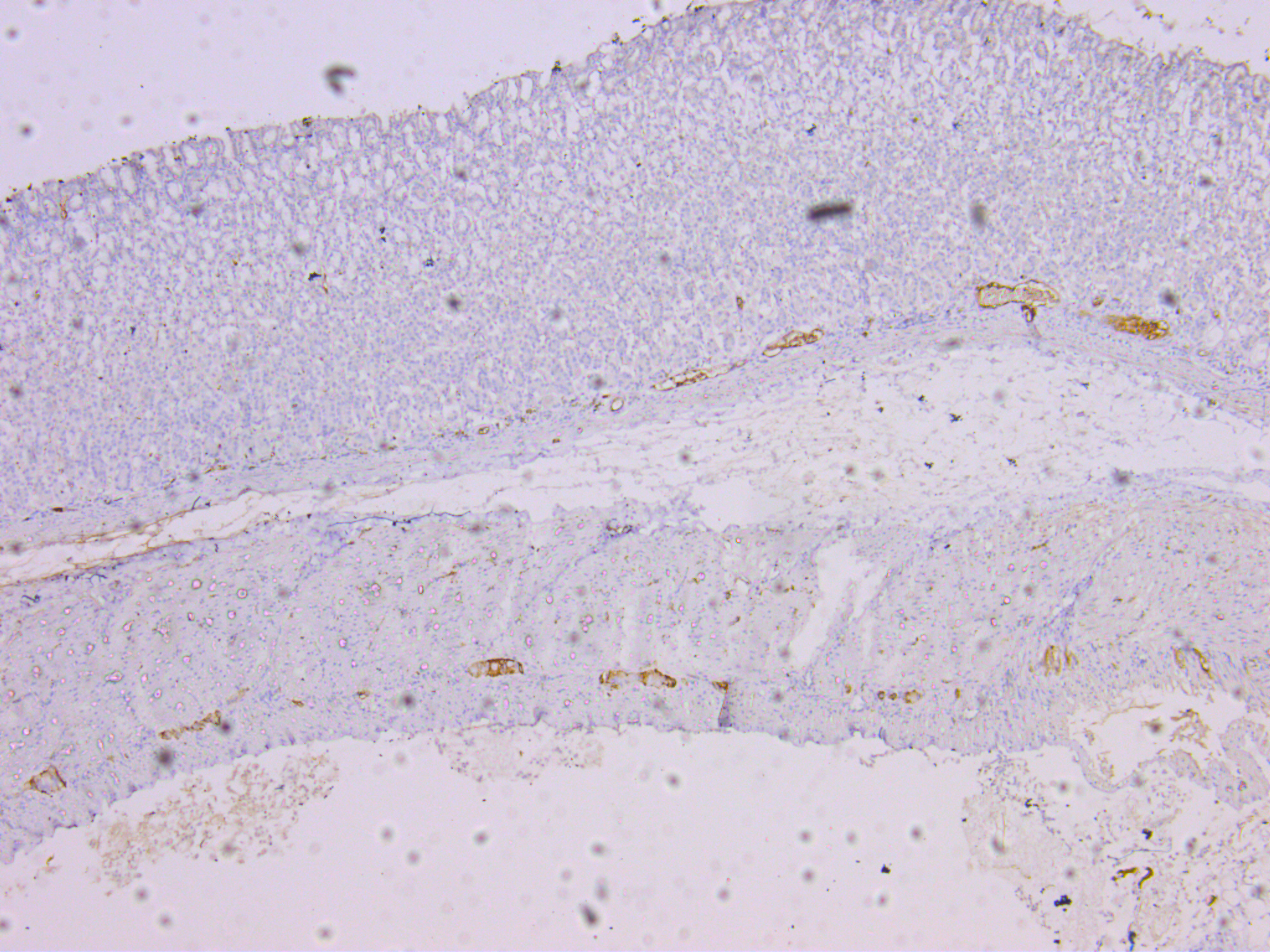

Supplement: Supplementary file 1 [file biomedicines-13-02564-s001.zip › Fig 5 Q x5 .tif]

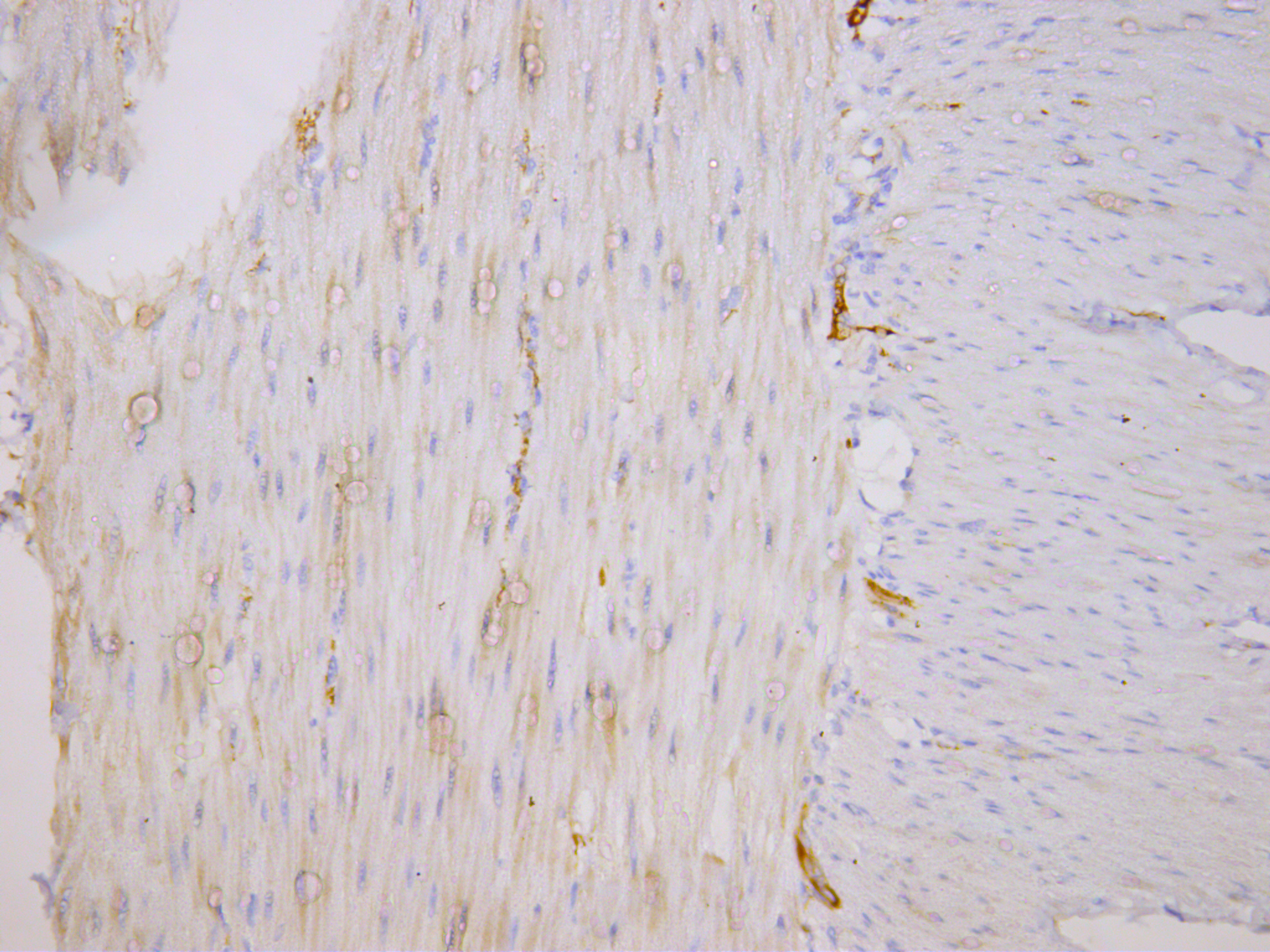

Supplement: Supplementary file 1 [file biomedicines-13-02564-s001.zip › Fig 5 R x20 .tif]

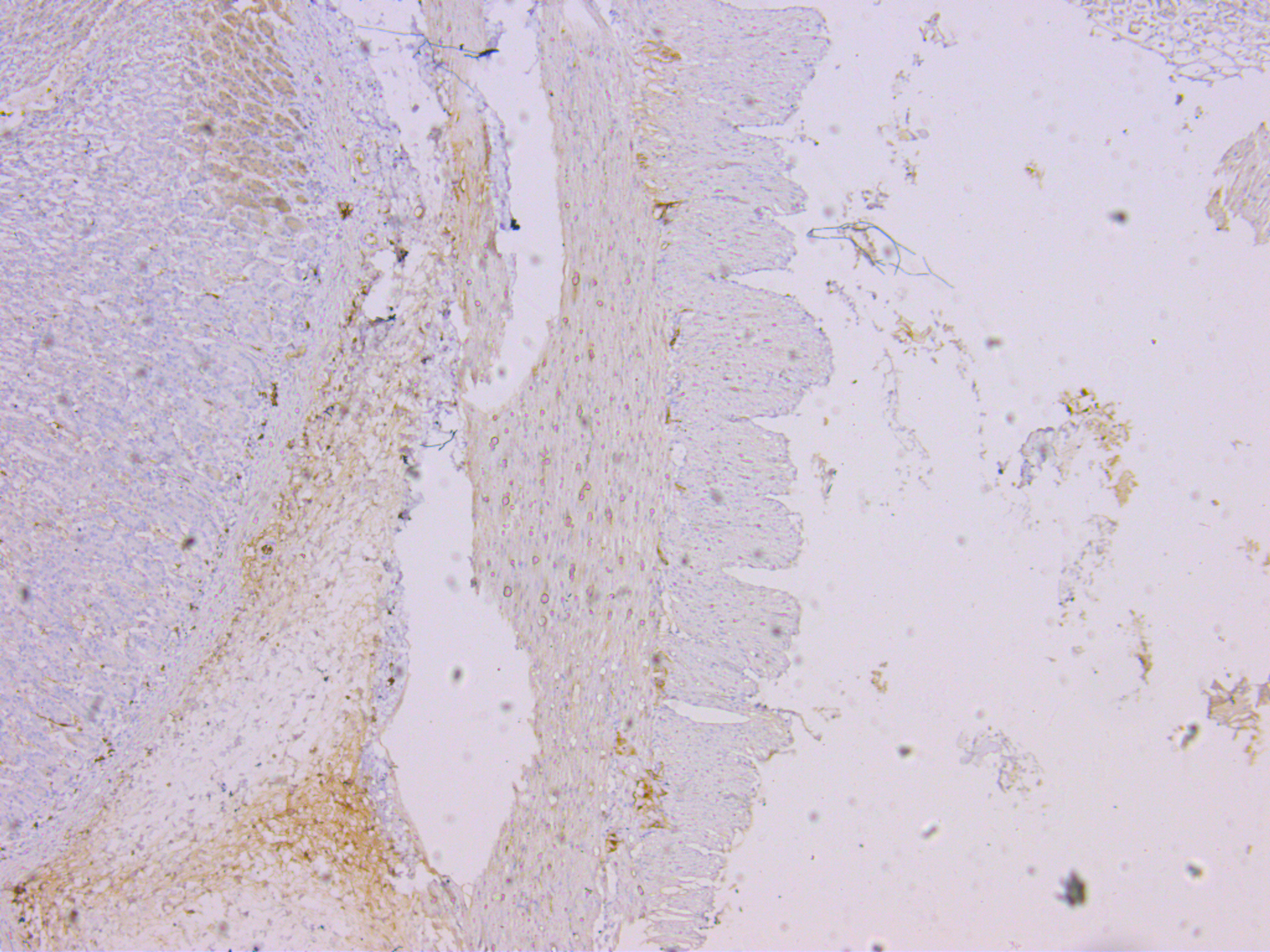

Supplement: Supplementary file 1 [file biomedicines-13-02564-s001.zip › Fig 5 R x5 .tif]
